# A noncanonical function of SKP1 regulates the switch between autophagy and unconventional secretion

**DOI:** 10.1126/sciadv.adh1134

**Published:** 2023-10-13

**Authors:** Jie Li, Gregory J. Krause, Qi Gui, Susmita Kaushik, Gergely Rona, Qingyue Zhang, Feng-Xia Liang, Avantika Dhabaria, Carlos Anerillas, Jennifer L. Martindale, Nikita Vasilyev, Manor Askenazi, Beatrix Ueberheide, Evgeny Nudler, Myriam Gorospe, Ana Maria Cuervo, Michele Pagano

**Affiliations:** ^1^ Department of Biochemistry and Molecular Pharmacology, New York University Grossman School of Medicine, New York, NY 10016, USA.; ^2^ Laura and Isaac Perlmutter NYU Cancer Center, New York University Grossman School of Medicine, New York, NY 10016, USA.; ^3^ Department of Developmental and Molecular Biology, Albert Einstein College of Medicine, Bronx, NY 10461, USA.; ^4^ Institute for Aging Research, Department of Medicine, Albert Einstein College of Medicine, Bronx, NY 10461, USA.; ^5^ Howard Hughes Medical Institute, New York University Grossman School of Medicine, New York, NY 10016, USA.; ^6^ Microscopy Laboratory, Division of Advanced Research Technologies, New York University Grossman School of Medicine, New York, NY 10016, USA.; ^7^ Proteomics Laboratory, Division of Advanced Research Technologies, New York University Grossman School of Medicine, New York, NY 10016, USA.; ^8^ Laboratory of Genetics and Genomics, National Institute on Aging Intramural Research Program, National Institutes of Health, Baltimore, MD 21224, USA.; ^9^ Biomedical Hosting LLC, 33 Lewis Avenue, Arlington, MA 02474, USA.

## Abstract

Intracellular degradation of proteins and organelles by the autophagy-lysosome system is essential for cellular quality control and energy homeostasis. Besides degradation, endolysosomal organelles can fuse with the plasma membrane and contribute to unconventional secretion. Here, we identify a function for mammalian SKP1 in endolysosomes that is independent of its established role as an essential component of the family of SCF/CRL1 ubiquitin ligases. We found that, under nutrient-poor conditions, SKP1 is phosphorylated on Thr^131^, allowing its interaction with V_1_ subunits of the vacuolar ATPase (V-ATPase). This event, in turn, promotes V-ATPase assembly to acidify late endosomes and enhance endolysosomal degradation. Under nutrient-rich conditions, SUMOylation of phosphorylated SKP1 allows its binding to and dephosphorylation by the PPM1B phosphatase. Dephosphorylated SKP1 interacts with SEC22B to promote unconventional secretion of the content of less acidified hybrid endosomal/autophagic compartments. Collectively, our study implicates SKP1 phosphorylation as a switch between autophagy and unconventional secretion in a manner dependent on cellular nutrient status.

## Teaser

Starvation-induced phosphorylation of SKP1 triggers a switch between protein secretion to degradation by autophagy.

## INTRODUCTION

Proteostasis is maintained by the coordinated function of chaperones and proteolytic systems (*1*). The timely and efficient degradation of proteins is essential for the maintenance of cellular function. Impaired protein degradation has been associated with a variety of conditions, including cancer (*2*) and neurodegenerative diseases (*3*, *4*). The two primary proteolytic systems in mammalian cells are the ubiquitin-proteasome system (UPS) and autophagy. In the UPS, substrate proteins are targeted for proteasomal degradation via ubiquitylation, which is mediated by a series of enzymes including the E3 ubiquitin ligases (*5*).

Several types of autophagy coexist in mammalian cells. The best described is macroautophagy, where cellular components, including proteins and organelles, are sequestered in double-membrane vesicles called autophagosomes (APGs) and then degraded upon APG fusion with lysosomes (*6*). Macroautophagy can be regulated at several steps, including APG formation, trafficking, and fusion with lysosomes, and it requires lysosome acidification for full maturation of the luminal proteases and to facilitate cargo unfolding (*7*). There is growing evidence that part of the autophagic machinery participates in secretion of sequestered cytosolic materials outside of the cell via unconventional [endoplasmic reticulum (ER)/Golgi-independent] secretion that involves fusing degradative compartments with the plasma membrane (*8*). This type of secretory-related fusion has been described for APGs (*9*, *10*), lysosomes (*11*), and late endosome/multivesicular bodies (LE/MVBs) (source of exosomes) (*12*), but the molecular underpinnings of a switch between degradation and secretion of autophagic cargo are poorly understood.

SKP1 is the assembly factor of SKP1–CUL1–F-box protein (SCF) complexes, also known as CUL1-RING ubiquitin ligases (CRL1s), a family of E3 ligases present in all eukaryotes (>70 members in mammals), which mediates the proteasomal degradation of hundreds of cellular regulatory proteins (*5*, *13*, *14*). Similar to ubiquitin, the ubiquitin-like protein SUMO can also be conjugated to lysine residues on target proteins (SUMOylation) through enzymatic steps involving E1-activating enzymes, E2-conjugating enzymes, and E3-ligases (*15*). Both conjugation of ubiquitin and of any of the three SUMO paralogs (SUMO-1, SUMO-2, and SUMO-3 in humans) (*16*) can alter the function or fate of the conjugated protein (*17*, *18*). Although SUMOylation regulates a plethora of biological functions, with the exception of RANGAP1, only a minor subpopulation of a SUMO target protein is SUMOylated at any given time (*19*). This paradoxical phenomenon has been termed the “SUMO enigma” (*19*–*21*). To shed light on this enigma, we used SKP1 as a model of a SUMO target protein since only a small fraction of endogenous, mammalian SKP1 has been reported to be SUMOylated (*22*, *23*), and the biological function of SKP1 SUMOylation remains unknown. In studying the biological impact of SKP1 SUMOylation, we identified a noncanonical role for SKP1 at LE/MVBs and show that posttranslational modifications of this protein regulate a nutrient-dependent interplay between macroautophagy and unconventional secretion independently of its well-established role in assembling SCF/CRL1 complexes. These studies reveal a previously unknown ability of SKP1 to regulate the switch between degradative and secretory functions of endolysosomal compartments.

## RESULTS

### SUMOylated SKP1 binds PPM1B to promote dephosphorylation of pThr^131^ in SKP1

**Fig. 1.**
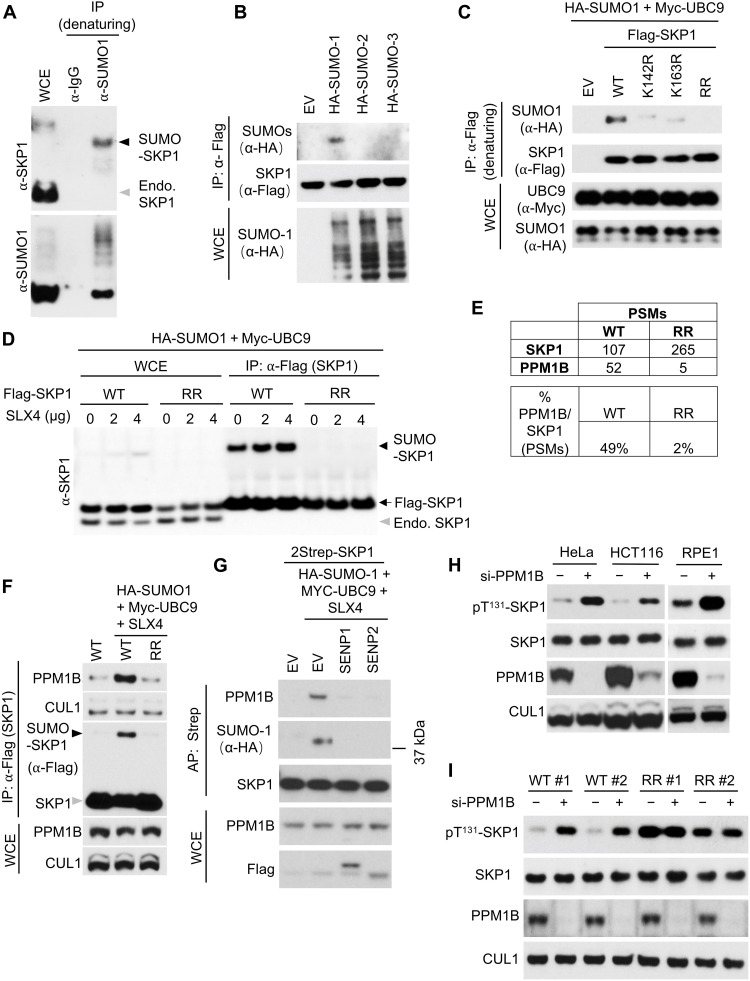
Binding of SUMOylated SKP1 to PPM1B decreases SKP1 phosphorylation on Thr^131^. (**A**) Immunoblot of denaturing IP with a control immunoglobulin G (IgG) or an antibody against SUMO1 in HCT116 cells. Arrowheads, SUMO-SKP1 (black) and endogenous SKP1 (Endo. SKP1) (gray). (**B**) Immunoblot after IP with Flag tag from HEK293T cells transfected with Flag-SKP1, Myc-UBC9, SLX4, and hemagglutinin (HA)–tagged SUMO-1, SUMO-2, or SUMO-3. Control without transfection of SUMO plasmids is shown in the left lane. Whole-cell extract (WCE) controls are shown at the bottom. (**C** and **D**) Immunoblot for indicated proteins after denaturing IP from lysates of HEK293T cells expressing Flag-tagged SKP1^WT^, SKP1^K142R^, SKP1^K163R^, or SKP1^K142R/K163R^ (SKP1^RR^). All cells expressed HA-SUMO1 and Myc-UBC9 plasmids with indicated amount of SLX4 plasmid. WCE controls are shown at the bottom (C). (**E** and **F**) Table of PSMs to SKP1 and PPM1B detected in the MS analysis (E) or immunoblot (F) for the proteins of Flag co-IP from HEK293T cells transfected with SUMOylation-enriching plasmids (HA-SUMO1, Myc-UBC9, and SLX4) and Flag-tagged SKP1^WT^ or SKP1^RR^. Control co-IP without transfection of SUMOylation-enriching plasmids is shown in the left lane in (F). WCE controls are shown at the bottom (F). (**G**) Immunoblot for proteins of affinity precipitation with Strep tag from HEK293T cells transfected with SUMOylation-enriching plasmids (HA-SUMO1, Myc-UBC9, and SLX4), 2Strep-SKP1, and Flag-tagged SENP1 or SENP2. Control without transfection of SUMOylation-enriching plasmids is shown in the left lane. WCE controls are shown at the bottom. (**H**) Immunoblot in HeLa, HCT116, and RPE1 cells after treatment with a control siRNA (−) or siRNA against PPM1B (si-PPM1B) (+). (**I**) Immunoblot of SKP1^WT^ and SKP1^RR^ clones after treatment with a control siRNA (−) or si-PPM1B (+). Each experiment was performed a minimum of three times with similar results.

As a first step to study SKP1 SUMOylation, we confirmed that endogenous SKP1 is SUMOylated in human cells by performing immunoprecipitation (IP) of endogenous SUMO-1 under denaturing conditions followed by Western blot with an anti-SKP1 antibody (Fig. 1A). Overexpression of SUMO-1, but not of SUMO-2 or SUMO-3, promoted SKP1 SUMOylation, suggesting a preference for SUMO-1 in SKP1 SUMOylation (Fig. 1B). SKP1 contains two potential SUMOylation motifs (*24*). Generation of a mutant in which K142 and K163 in these two motifs are converted to arginine (SKP1^RR^) completely abolished SUMOylation of SKP1 in human embryonic kidney (HEK) 293T cells, in the absence and presence of either SLX4 or PIAS3, two SUMO E3 ligases that enhance SKP1 SUMOylation (Fig. 1, C and D, and fig. S1, A and B).

One possible explanation for the SUMO enigma is that SUMOylation transiently recruits enzymes that, in turn, modify SUMO substrates in a more stable fashion, promoting additional modifications that remain after SUMO is itself removed from the substrate. To identify binding partners of SUMOylated SKP1, we expressed SKP1^WT^ or SKP1^RR^ in HEK293T cells under the condition that promotes maximal SKP1 SUMOylation shown in Fig. 1D. We then immunoprecipitated SKP1^WT^ and SKP1^RR^ and analyzed copurified proteins via mass spectrometry (MS) analysis. We found 52 PSMs (peptide-spectrum matches) corresponding to the phosphatase PPM1B in the SKP1^WT^ IP compared to only 5 PPM1B PSMs in the SKP1^RR^ IP (Fig. 1E and table S1). Normalization based on the number of SKP1 peptides showed that the PPM1B/SKP1 PSM ratio was 49% for SKP1^WT^ and 2% for SKP1^RR^ (Fig. 1E). This difference was confirmed via Western blot (Fig. 1F). To further investigate whether SKP1 interacts with PPM1B in a SUMO-dependent manner, we overexpressed the SUMO-specific proteases SENP1 and SENP2 (*25*) that remove SUMO conjugated to proteins, and found this to be sufficient to abolish the interaction between SKP1 and PPM1B (Fig. 1G). These data indicate that SKP1 SUMOylation promotes the interaction of SKP1 with PPM1B.

We next identified the protein region involved in the SUMO-dependent interaction of SKP1 with PPM1B. SUMOylation can promote protein-protein interaction of the SUMOylated protein through a SUMO-interacting motif (SIM) in the interacting partner (*26*). This SIM is typically four amino acid residues in length and often matches either an hXhh consensus or an hhXh consensus, in which h is an amino acid with a large nonpolar aliphatic side chain (Ile, Val, or Leu) and X is any amino acid. We found that PPM1B contains a conserved SIM (positions 290 to 293 in humans) (fig. S1C) and investigated its involvement in the SKP1-PPM1B interaction by generating a SIM mutant of PPM1B (PPM1B^SIM-4A^) in which residues 290 to 293 were substituted with alanine. We expressed PPM1B^WT^ or PPM1B^SIM-4A^ with SKP1 in HEK293T cells under conditions that promote SKP1 SUMOylation and found that SUMOylated SKP1 coimmunoprecipitated with PPM1B^WT^ but not with PPM1B^SIM-4A^ (fig. S1D), in support of the interaction occurring through the PPM1B SIM region.

We then asked whether SKP1 is a substrate of PPM1B, and to this end, we analyzed SKP1 phosphorylation sites by MS and found that Thr^131^ was the only site to be phosphorylated (fig. S2A). This phosphorylation site is conserved from yeast to humans (fig. S2B), and PhosphoSitePlus (*27*) reports 34 high-throughput studies that identified phosphorylation at this site, supporting our finding. We generated a phospho-specific antibody that recognizes SKP1 phosphorylated on T131 (pT^131^-SKP1), but not SKP1^T131A^, SKP1^T131D^, and SKP1^T131E^ mutants (fig. S2C). Using this antibody, we found that SKP1^RR^, the mutant that cannot be SUMOylated, is phosphorylated approximately threefold more than SKP1^WT^, as shown by both Western blot and MS analysis of the IPs (fig. S2, D and E). Silencing of PPM1B increased the abundance of pT^131^-SKP1 in HeLa, HCT116, and RPE1 cells (Fig. 1H).

Next, we expressed at near-physiologic levels either Flag-tagged SKP1^WT^ or Flag-tagged SKP1^RR^ in HeLa cells. Subsequently, we knocked out all five genomic *SKP1* alleles using CRISPR-Cas9 technology and isolated two independent clones (fig. S2F). Silencing PPM1B in two different clones expressing SKP1^WT^ induced an increase in pT^131^-SKP1 levels (Fig. 1I) similar to what was observed in other cell lines (Fig. 1H). In contrast, PPM1B silencing in two clones expressing SKP1^RR^ did not show this increase (Fig. 1I), supporting our hypothesis that SUMOylation of a subpopulation of SKP1 regulates its PPM1B-mediated dephosphorylation.

### SKP1 phosphorylation on Thr^131^ promotes its interaction with the V-ATPase, independently of its binding to F-box proteins

SKP1 is an essential subunit of the SCF/CRL1 family of ubiquitin ligases (*5*, *14*). The increase in the levels of pT^131^-SKP1 in response to PPM1B silencing did not affect the binding between SKP1 and CUL1 or any of the tested F-box proteins (FBXO1, FBXO11, FBXO18, and FBXW1) (fig. S3A). Similarly, it did not modify the binding between CUL1 and F-box proteins, as detected by IP followed by either Western blot (fig. S3B) or MS analysis (fig. S3, C and D, and table S2). Moreover, during a release from a 4-hour incubation with CSN5i, a CUL1 deneddylation inhibitor, PPM1B silencing did not affect the dynamics of CUL1 neddylation, which is a proxy for the activity of cellular CRL1s (fig. S3E). Similarly, when PPM1B was silenced, we observed no changes in the abundance of F-box proteins coimmunoprecipitated with SKP1 (fig. S3F).

We then generated two independent clones of HeLa cells expressing Flag-tagged SKP1^T131A^ at near-physiologic levels (fig. S3G), as done for the clones shown in fig. S2F. MS analysis of proteins pulled down with either SKP1^WT^, SKP1^RR^, or SKP1^T131A^ did not show any substantial changes in the abundance of coimmunoprecipitated F-box proteins (fig. S3, H and I, and tables S3 and S4).

**Fig. 2.**
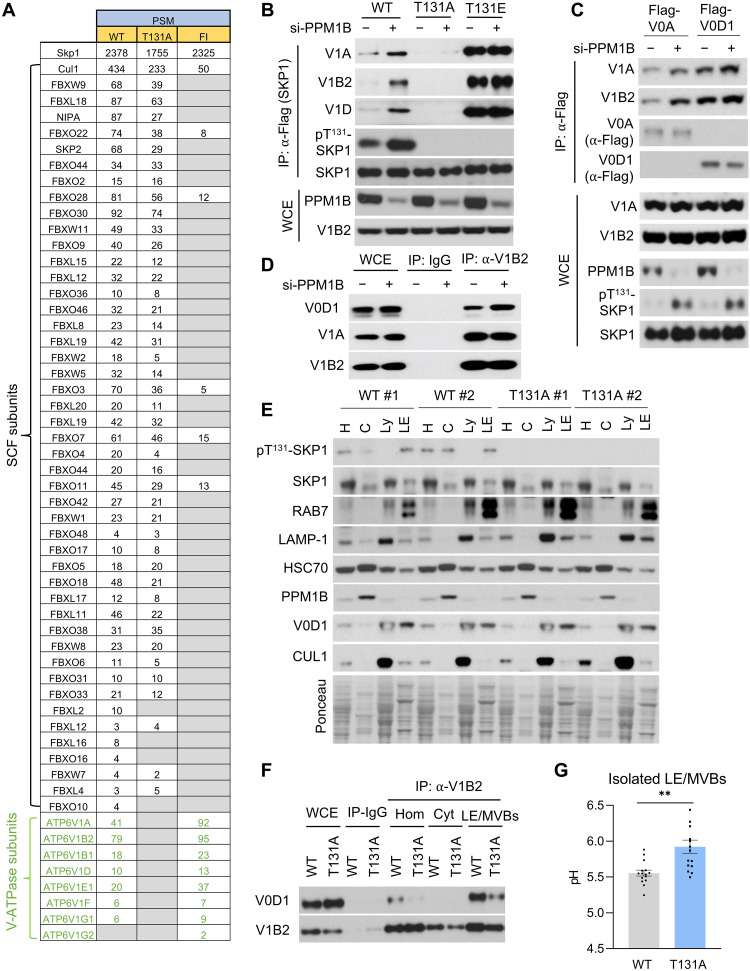
SKP1 phosphorylation on Thr^131^ promotes its interaction with the V-ATPase and V-ATPase assembly, independently of its binding to F-box proteins. (**A**) Table of PSMs to proteins coimmunoprecipitated (co-IPed) from HEK293T cells transfected with SKP1^WT^, SKP^T131A^ (phospho-mutant), and SKP1^F139A/I41A^ (SKP1^FI^; F-box binding mutant) detected in the MS analysis. (**B**) Immunoblot of Flag co-IP from HEK293T cells transfected with Flag-tagged SKP1^WT^, SKP1^T131A^, and SKP1^T131E^ (phospho-mimetic) after treatment without (−) or with (+) siRNA to PPM1B (si-PPM1B). WCE controls are shown at the bottom. (**C**) Immunoblot of proteins co-IPed from cells expressing Flag-tagged V_0_A or V_0_D1 after treatment with (+) or without (−) si-PPM1B. WCE controls are shown at the bottom. (**D**) Immunoblot of proteins co-IPed with endogenous V_1_B2 in cells treated without (−) or with (+) si-PPM1B. WCE controls are shown on the left, and IP with control rabbit IgG is in the middle lanes. (**E**) Immunoblot of proteins in homogenate (H), cytosol (C), lysosomes (Ly), or LE/MVBs isolated from cells expressing SKP1^WT^ or SKP1^T131A^. (**F**) Immunoblot of proteins co-IPed with endogenous V_1_B2 in homogenate (Hom), cytosol (Cyt), or LE/MVBs isolated from cells expressing SKP1^WT^ or SKP1^T131A^. WCE and co-IPs with control IgGs (IP-IgG) are shown on the left lanes. (**G**) Measurement of pH in LE/MVBs isolated from SKP1^WT^ or SKP1^T131A^ cells using the ratiometric LysoSensor Yellow/Blue DND-160 probe. *n* = 15 measurements from three independent experiments. Each experiment was performed a minimum of three times with similar results. Data are means ± SEM and individual values. Unpaired *t* test (G) was used. Differences were significant for **P < 0.01.

To identify proteins that interact with SKP1 in a phosphorylation-dependent manner, but independently of SKP1 interaction with F-box proteins, we generated a SKP1 mutant that does not bind F-box proteins but is still phosphorylated in human cells. We performed an in-depth alanine scanning mutagenesis of the SKP1 region that is involved in binding the F-box domain as shown by the crystal structure of the SKP1-SKP2 complex (*28*). Pull-down experiments revealed that the SKP1^F139A/I141A^ mutant (SKP1^FI^) prevented F-box protein binding while maintaining SKP1 phosphorylation (fig. S4, A and B). MS analysis of proteins pulled down with transiently expressed Flag-tagged SKP1^WT^, SKP1^T131A^ (phospho-mutant), or SKP1^FI^ (mutant that does not bind F-box proteins but is still phosphorylated on Thr^131^) confirmed that F-box proteins abundantly coimmunoprecipitated with both SKP1^WT^ and SKP1^T131A^, but not SKP1^FI^ (Fig. 2A and tables S5 and S6). Moreover, MS analysis revealed that eight subunits of the V_1_ domain of the vacuolar adenosine triphosphatase (ATPase) (V-ATPase) coimmunoprecipitated with SKP1^WT^ and SKP1^FI^, but were completely absent in the SKP1^T131A^ pull-downs (Fig. 2A and table S6). This finding indicates that binding of SKP1 to V-ATPase, which has also been reported in yeast (*29*), is phosphorylation-dependent and occurs independently of F-box proteins. A genetic interaction between SKP1 and the V-ATPase was confirmed using the Depmap portal (https://depmap.org/), which shows that *SKP1* dependencies, but not *CUL1* dependencies, directly correlated with *ATP6V1C1,* which encodes the V_1_C regulatory subunit of V1 (*30*). V-ATPase subunits and F-box proteins did not appear to compete with each other, as overexpression of the F-box proteins SKP2 or β-TrCP did not decrease the binding between SKP1 and V-ATPase components (fig. S4C). Co-IP from cells expressing Flag-tagged SKP1 mutants confirmed lack of interaction between SKP1^T131A^ and V_1_ V-ATPase subunits and showed increased binding of the V_1_ V-ATPase subunits with the phospho-mimetic SKP1^T131E^ protein (Fig. 2B). Accordingly, the SKP1^RR^ mutant, which is unable to be SUMOylated (Fig. 1C), binds less PPM1B (Fig. 1, E and F), is more phosphorylated (Fig. 1I), and displayed enhanced interaction with V-ATPase subunits (fig. S4D). Last, knockdown of PPM1B increased SKP1 phosphorylation, as well as enhanced interaction of SKP1^WT^ with the V_1_ V-ATPase components, while displaying no effect on the SKP1^T131E^ interaction with V_1_ subunits (Fig. 2B), consistent with the interaction being driven by SKP1 phosphorylation.

### SKP1 phosphorylation promotes assembly of the V-ATPase

V-ATPase activity can be regulated by modulating assembly of the V_1_ and V_0_ domains of the V-ATPase (*31*). We transduced HEK293T cells with Flag-tagged versions of V_0_A or V_0_D1 and observed that higher levels of endogenous V_1_A and V_1_B2 were pulled down with both V_0_ subunits when PPM1B was silenced to increase levels of SKP1 phosphorylation (Fig. 2C). Similarly, PPM1B silencing also increased the amount of endogenous V_0_D1 coimmunoprecipitated with endogenous V_1_B2 (Fig. 2D), suggesting enhanced assembly of the full V-ATPase when SKP1 is phosphorylated.

To determine in which cellular acidic compartment the interaction between p^T131^-SKP1 and V-ATPase subunits takes place, we compared the subcellular distribution of SKP1 in lysosome- and LE/MVB-enriched fractions isolated from cells expressing either SKP1^WT^ or SKP1^T131A^. We found that p^T131^-SKP1 is predominantly detected in LE/MVBs, despite total SKP1 being more abundant in lysosomes compared to LE/MVBs (Fig. 2E). Moreover, we found that the lysosome fractions contain much more CUL1 (a subunit of SCF/CRL1 ubiquitin ligase complexes) than the LE/MVB fractions (Fig. 2E). Incubation of LE/MVBs with increasing concentrations of trypsin to degrade surface proteins, but not those protected by the LE/MVB membrane (unless disrupted with a detergent), revealed that most p^T131^-SKP1 was located on the surface of LE/MVBs (fig. S5A). IP of endogenous V_1_B2 revealed higher interaction of this subunit with endogenous V_0_D1 in LE/MVBs isolated from cells expressing SKP1^WT^ compared to those expressing SKP1^T131A^ (Fig. 2F; inputs in fig. S5B). This finding is consistent with p^T131^-SKP1 positively regulating V-ATPase assembly in the LE/MVB compartment.

### Nutrient starvation induces SKP1 phosphorylation, increasing LE/MVB acidification

To investigate whether the enhanced V-ATPase assembly mediated by p^T131^-SKP1 affects the acidification of LE/MVBs, we used the ratiometric probe LysoSensor Yellow/Blue DND-160 to measure the pH of isolated LE/MVBs. We found that LE/MVBs isolated from SKP1^T131A^ cells had a higher pH (6.1) compared to those isolated from SKP1^WT^ cells (pH 5.6) (Fig. 2G), supporting our hypothesis that preferential localization of p^T131^-SKP1 at LE/MVBs promotes higher acidification of this compartment.

**Fig. 3.**
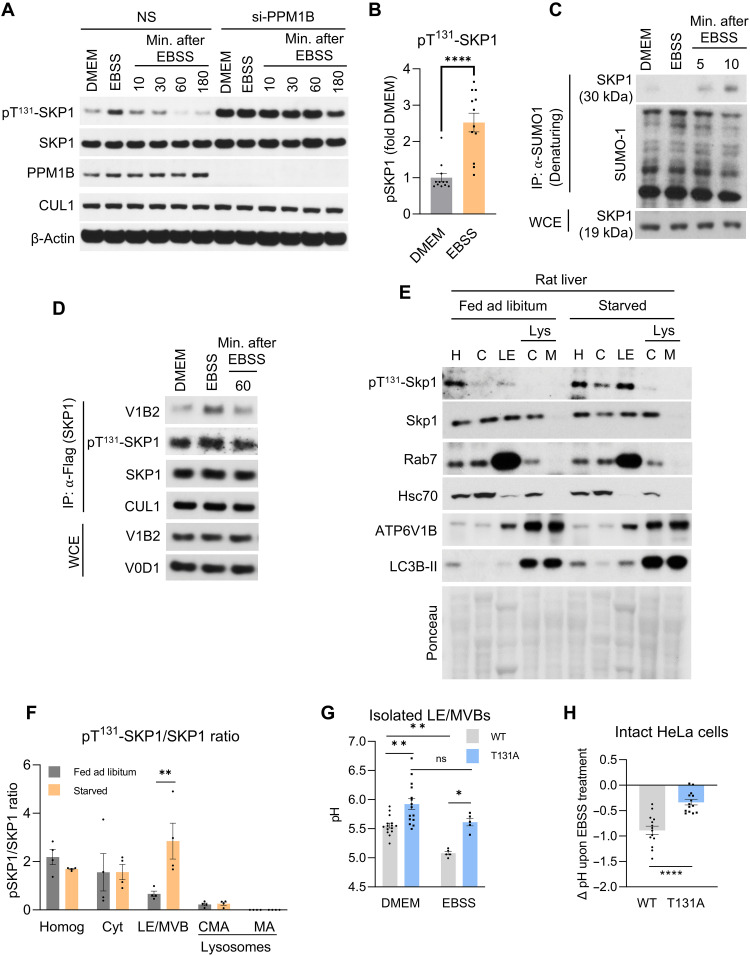
Nutrient deprivation induces SKP1 phosphorylation, enhancing LE/MVB acidification. (**A** and **B**) Representative immunoblot (A) of HeLa cells treated with a control siRNA (NS) or si-PPM1B and incubated in complete DMEM or at indicated times after 1-hour incubation in EBSS and subsequent reintroduction of full DMEM (Min. after EBSS). Quantification of change in pT^131^-SKP1 levels upon 1-hour EBSS incubation is shown in (B). *n* = 6 independent experiments. (**C** and **D**) Immunoblot of proteins co-IPed with endogenous SUMO-1 in HeLa cells (C) or with Flag-tagged SKP1 in cells expressing this protein (D) maintained in complete DMEM or at indicated times after 1-hour incubation in EBSS and subsequent reintroduction of full DMEM (Min. after EBSS). (**E** and **F**) Representative immunoblot (E) of the indicated fractions isolated from livers of rats fed ad libitum (Fed) or starved for 24 hours (Starved). Homogenate (H), cytosol (C), LE/MVBs (LE), and lysosomes active for chaperone-mediated autophagy (Lys C) or macroautophagy (Lys M). Quantification (F) of pT^131^-SKP1 levels relative to total SKP1 levels in each fraction. *n* = 5 rats. (**G** and **H**) pH measured with ratiometric LysoSensor Yellow/Blue DND-160 probe in isolated LE/MVBs from SKP1^WT^ or SKP1^T131A^ cells maintained in DMEM or EBSS (G) and change (Δ) in pH in HeLa cells expressing SKP1^WT^ or SKP1^T131A^ upon incubation in EBSS for 1 hour (H). *n* = 5 or 15 independent experiments (G) or 7 independent experiments (H). Each experiment was performed a minimum of three times with similar results. Data are means ± SEM and individual values. Unpaired *t* test [(B) and (H)] and two-way ANOVA with Bonferroni’s multiple comparisons post hoc test [(F) and (G)] were used. Differences were significant for *P < 0.05, **P < 0.01, ****P < 0.0001. ns, not significant.

Enhanced acidification of LE/MVBs promotes maturation of resident hydrolases and increases the overall degradative capacity of these compartments (*32*). To start elucidating the physiological relevance of the effect of p^T131^-SKP1 on V-ATPase activity in LE/MVBs, we analyzed the phosphorylation state of SKP1 during starvation, a condition associated with increased endolysosomal protein degradation (*33*). Comparison of the fraction of SKP1 phosphorylated in HeLa cells grown in complete medium with HeLa cells maintained for 1 hour in Earle’s balanced salt solution (EBSS), which lacks any nutrients, revealed a ~2.5-fold increase in p^T131^-SKP1 levels during starvation (Fig. 3, A and B). Upon replacement of EBSS with complete medium, the levels of p^T131^-SKP1 steadily returned to basal levels within 30 min (Fig. 3A). Nutrient-dependent changes in SKP1 phosphorylation seem to be mostly mediated by active dephosphorylation, since starvation did not further increase levels of p^T131^-SKP1 in PPM1B knockdown cells (Fig. 3A). This pattern of endogenous SKP1 phosphorylation was also observed in noncancerous RPE1 cells (fig. S6A). As expected, upon nutrient repletion, the decline in SKP1 phosphorylation was matched by an increase in endogenous SUMOylation of SKP1 (Fig. 3C), which we have shown to facilitate SKP1 dephosphorylation by promoting binding to the PPM1B phosphatase (Fig. 1, E to H). The sensitivity of SKP1 phosphorylation to nutrient status is reminiscent of the mammalian target of rapamycin complex 1 (mTORC1), known to regulate endolysosomal properties, including acidification, in a nutrient-dependent manner (*34*). To test whether mTORC1 signaling regulates the phosphorylation state of SKP1, we treated HeLa and RPE1 cells with the mTORC1 inhibitor Torin 1 (fig. S6, B and C; reduction in phosphorylation of the mTOR substrate S6K is used as a positive control of Torin 1 efficacy). We found that Torin 1 did not enhance the levels of SKP1 phosphorylation, suggesting that the nutrient starvation–induced increase in SKP1 phosphorylation is independent of mTORC1 activity.

The interaction between p^T131^-SKP1 and V_1_B2 was enhanced after EBSS starvation and decreased after restoration of complete medium (Fig. 3D). We obtained similar results in vivo using subcellular fractionation of livers from rats fed ad libitum or starved for 24 hours, where we also observed an increase in Skp1 phosphorylation during starvation that occurred predominantly in LE/MVBs (Fig. 3, E and F). In contrast, we found low levels of p^T131^-Skp1 that did not change with nutrient status in two lysosomal populations that participate in macroautophagy or chaperone-mediated autophagy (*35*), despite both processes being activated by starvation (Fig. 3, E and F). These results further reinforce the notion that the starvation-induced increase in SKP1 phosphorylation mainly affects SKP1 at LE/MVBs.

Next, we measured the pH of LE/MVBs isolated from cells expressing SKP1^WT^ or SKP1^T131A^ in response to starvation with EBSS. While SKP1^WT^ cells were able to acidify LE/MVBs during nutrient deprivation (pH of 5.6 to 5.3), LE/MVBs from SKP1^T131A^ cells displayed a more basic pH that did not acidify in response to nutrient removal (Fig. 3G). The inability of SKP1^T131A^ cells to further acidify the endolysosomal compartment during nutrient deprivation was also observed when measuring pH in intact HeLa cells (Fig. 3H). Last, we analyzed whether this relation of SKP1 phosphorylation with LE/MVB acidification was bidirectional. We used a variety of agents known to reduce endolysosomal acidification, including bafilomycin A1, concanamycin A, KM91104, apillimod, and chloroquine, and found that none of them affected SKP1 phosphorylation levels (fig. S6D). These findings suggest that while SKP1 phosphorylation can affect LE/MVB acidification, the degree of LE/MVB acidification does not influence SKP1 phosphorylation. Overall, these findings support that, upon nutritional deprivation, phosphorylation of SKP1 at LE/MVBs promotes assembly of the V-ATPase complex and leads to further acidification of this compartment.

### SKP1 phosphorylation enhances endolysosomal degradative capacity during starvation

**Fig. 4.**
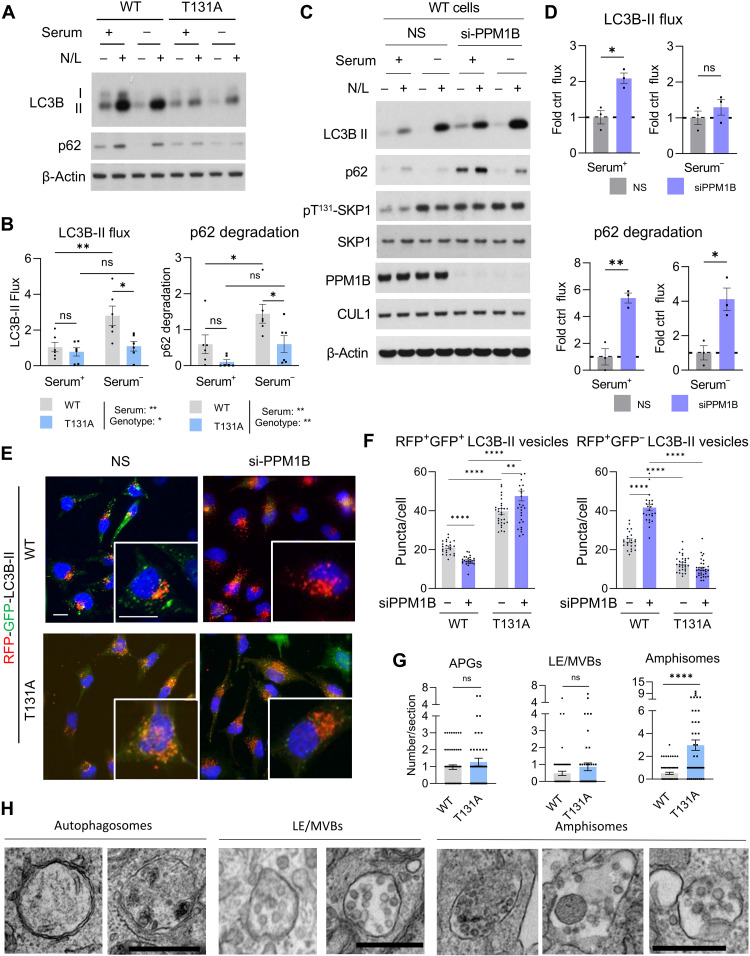
SKP1 phosphorylation enhances amphisome degradative capacity during starvation. (**A** and **B**) Immunoblot (A) of SKP1^WT^ or SKP1^T131A^ HeLa cells grown in DMEM supplemented with (+) or without (−) serum for 8 hours. Where indicated, cells were treated with ammonium chloride and leupeptin (N/L) to inhibit endolysosomal proteolysis. Quantification (B) of LC3B-II flux (left) and p62 degradation (right). *n* = 6 independent experiments. (**C** and **D**) Immunoblot (C) of HeLa cells treated without (−) or with (+) si-PPM1B after incubation in DMEM supplemented with (+) or without (−) serum for 8 hours. Where indicated, cells were treated with N/L. Quantification (D) of LC3B-II flux (top) and p62 degradation (bottom). *n* = 3 to 6 independent experiments. (**E** and **F**) Macroautophagy activity in cells expressing SKP1^WT^ and SKP1^T131A^ transduced with the GFP-RFP-LC3B tandem fluorescent reporter and with si-PPM1B or a control (NS). Representative images (E) and quantification (F) of neutral pH LC3B^+^ compartments (GFP^+^; RFP^+^ puncta) (left) and LC3B^+^ acidic compartments (GFP^−^; RFP^+^ puncta) (right). *n* > 250 cells for two cell lines each. Nuclei are highlighted with 4′,6-diamidino-2-phenylindole (DAPI). (**G**) Morphometric quantification from electron micrographs of the abundance of APGs, LE/MVBs, and amphisomes in HeLa cells expressing SKP1^WT^ or SKP1^T131A^. Images were independently quantified by two experts blinded to the cell line genotype. *n* ≥ 20 fields per cell line, two cell lines each. (**H**) Representative electron micrographs of APGs, LE/MVBs, and amphisomes in SKP1^T131A^ cells. Scale bar, 0.5 μm. Data are means ± SEM and individual values. Two-way ANOVA with Tukey’s multiple comparisons post hoc test [(B) and (F)] and unpaired *t* test [(D) and (G)] were used. Two-way ANOVA with Tukey’s multiple comparisons post hoc test for (D) is shown in the raw and statistic data file to show significant increase for both conditions upon serum removal. Differences were significant for *P < 0.05, **P < 0.01, ***P < 0.001, ****P < 0.0001.

Given the effect of SKP1 phosphorylation on LE/MVB acidification, we investigated possible changes in endolysosomal degradation, focusing on the impact on macroautophagy. We monitored autophagy flux (formation and degradation of autophagic compartments) as changes in the abundance of LC3B-II (integral component of autophagic vesicles) upon blocking endolysosomal proteolysis by treatment of cells with a combination of ammonium chloride (to neutralize lysosomal acidification) and leupeptin (to inhibit endolysosomal proteases) (*36*). To obtain information on the amount of degradation occurring through macroautophagy, we also measured the changes in levels of the macroautophagy receptor p62 (which undergoes degradation with autophagic cargo) (*37*). In agreement with the observed changes in acidification, we found that preventing phosphorylation of SKP1 (SKP1^T131A^ cells) ablated the expected up-regulation of macroautophagy flux upon serum removal, detected as a failure to increase the amount of LC3B-II and, to some extent, p62 degradation (Fig. 4, A and B, note that the LC3B antibody used displays higher affinity for LC3B-II than LC3B-I, which is barely detected in these cells). Conversely, enhancing SKP1 phosphorylation by treatment of cells with small interfering RNA (siRNA) to PPM1B was sufficient to promote increased basal autophagic activity, as measured by LC3B-II flux and p62 degradation (Fig. 4, C and D). As expected, knockdown of PPM1B had no effect on LC3B-II flux or p62 degradation in cells expressing an SKP1 mutant that cannot be phosphorylated (fig. S7, A and C).

We also measured macroautophagy activity using a tandem fluorescent reporter [red fluorescent protein (RFP)–green fluorescent protein (GFP)–LC3B] that provides information on the pH of LC3B-positive compartments (LC3B^+^) (*38*). In LC3B^+^ vesicles with a neutral pH, both fluorophores maintain their fluorescence (GFP^+^/RFP^+^ puncta), whereas in LC3B^+^ compartments with an acidic pH, GFP fluorescence is quenched and only RFP fluorescence remains visible (GFP^−^/RFP^+^ puncta) (*38*). Under nutrient-rich conditions, PPM1B knockdown (which increases SKP1 phosphorylation) in cells stably expressing this reporter resulted in an increase in LC3B^+^ acidic compartments (fig. S7, D and E, right) without increasing the number of neutral pH LC3B^+^ vesicles (fig. S7, D and E, left), compatible with increased acidification of LC3B^+^ compartments. Nutrient removal in PPM1B-silenced cells also induced an increase in LC3B^+^ acidic compartments that, in this case, was also associated with a higher number of neutral pH LC3B^+^ vesicles (fig. S7, D and E). The increase in the overall number of LC3B^+^ compartments in PPM1B knockdown cells upon nutrient removal (fig. S7, D and E, bottom) indicates some level of autophagy induction along with the observed enhanced clearance of autophagic compartments. We then repeated similar experiments in cells expressing either SKP1^WT^ or SKP1^T131A^ under nutrient-rich conditions and found that in cells where phosphorylation of SKP1 was not possible, there was a significant reduction in the number of LC3B^+^ acidic compartments that associated with a pronounced increase in neutral pH LC3B^+^ vesicles (Fig. 4, E and F, right). Furthermore, the increase in acidification of LC3B^+^ vesicles observed in cells expressing SKP1^WT^ upon PPM1B silencing was not detected in SKP1^T131A^-expressing cells (Fig. 4, E and F, right).

Degradation of APG contents can occur after direct APG fusion with lysosomes or by an initial fusion of APGs with LE/MVBs to form intermediary compartments termed amphisomes (*39*). We studied the effect of SKP1 phosphorylation on the different autophagic compartments, but because amphisomes are difficult to isolate, we instead used transmission electron microscopy (EM) and morphometric analysis. Following standard criteria (*40*), APGs are identified as double-membrane vesicles with density of contents and appearance comparable to the surrounding cytoplasm, LE/MVBs as single-membrane vesicles containing only uniform small vesicles in the lumen of an approximate diameter of 100 nm, and amphisomes as single- or partially double-membrane vesicles containing in their lumen a combination of vesicles of similar characteristics to those in LE/MVBs and additional cytosolic materials. Analysis of the number of each of these autophagy-related compartments per field revealed that SKP1^T131A^-expressing cells did not display a significant increase in APGs (Fig. 4G), one of the possible origins of the neutral pH LC3B^+^ vesicles that we found accumulated in these cells (Fig. 4F, left), suggesting that the LC3B^+^ compartments that failed to mature in SKP1^T131A^ cells were not APGs. Since SKP1 phosphorylation seems to occur primarily in LE/MVBs, we also analyzed the abundance of LE/MVBs and LE/MVB-related autophagic compartments. The number of LE/MVBs in SKP1^T131A^ cells showed a discrete, albeit not significant, increase, but the most pronounced difference was the almost sixfold increase in the number of amphisomes (Fig. 4G; examples of APGs, LE/MVBs, and amphisomes in SKP1^T131A^ cells are shown in Fig. 4H). The increase in amphisomes in SKP1^T131A^ cells was further confirmed upon expression of RFP-GFP-LC3B and incubation with Alexa Fluor 647–bovine serum albumin (BSA) to label the endocytic compartment. As shown in fig. S8 (A and B), SKP1^T131A^ cells show a significant increase in LC3B^+^ compartments also labeled for the endocytic cargo.

These findings suggest that during starvation, the phosphorylation of SKP1 at LE/MVBs and the subsequent acidification of this compartment promote enhanced autophagic cargo degradation. In agreement with this conclusion, analysis of long-lived protein degradation upon metabolic labeling confirmed reduced endolysosomal proteolysis in SKP1^T131A^-expressing cells upon serum removal (fig. S8C).

### SKP1 dephosphorylation leads to unconventional secretion of amphisome cargo

**Fig. 5.**
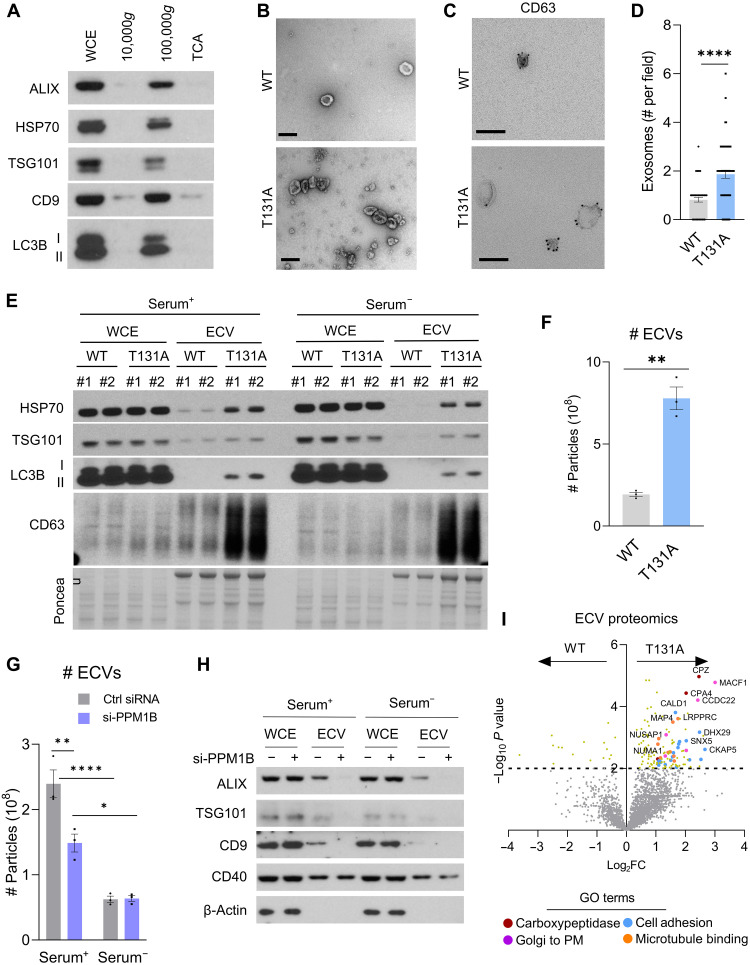
SKP1 dephosphorylation promotes exosome secretion. (**A**) Immunoblot of cell culture medium subjected to differential ultracentrifugation to recover large ECVs (10,000*g*), small ECVs (100,000*g*), and precipitated free soluble protein (TCA). (**B**) Representative transmission electron micrographs of isolated ECVs. Scale bar, 200 nm. (**C** and **D**) Representative images from immune-EM with an antibody against CD63 (C) and quantification (D) of CD63^+^ vesicles in the small ECV fraction from SKP1^WT^ and SKP1^T131A^ HeLa cells. Scale bars, 200 μm; *n* = 60 images. (**E**) Immunoblot of WCE and ECVs from SKP1^WT^ and SKP1^T131A^ HeLa cells cultured in DMEM with (+) or without (−) serum for 16 hours. Ponceau shows different electrophoretic patterns. (**F** and **G**) Number of vesicles by NanoFCM analysis in ECV fractions isolated from culture medium of SKP1^WT^ and SKP1^T131A^ cells (F) or SKP1^WT^ cells treated with control siRNA (Ctrl) or si-PPM1B in Serum^+^ or Serum^−^ conditions for 16 hours. (G). *n* = 3 independent experiments. (**H**) Immunoblot of WCE and ECVs from HeLa cells expressing SKP1^WT^ and treated without (−) or with (+) si-PPM1B cultured in DMEM supplemented (+) or not (−) with serum for 16 hours. (**I**) Log_2_ fold change (Log_2_FC) in protein abundance between ECVs isolated from SKP1^WT^ or SKP1^T131A^ HeLa cells. Gray, nonsignificant proteins. Enriched GO terms are color-labeled, and representative proteins for each pathway are shown. Each experiment was performed a minimum of three times with similar results. Data are means ± SEM and individual values. Unpaired *t* test [(D) and (F)] or two-way ANOVA with Bonferroni’s multiple comparisons post hoc test (G) was used. Differences were significant for *P < 0.05, **P < 0.01, ****P < 0.0001.

Multiple studies support a close relationship between protein degradation and protein secretion pathways as important to the maintenance of cellular proteostasis (*41*, *42*). In addition to the conventional secretory pathway, whereby proteins are trafficked through the ER-Golgi pathway and then released extracellularly through plasma membrane docking of secretory vesicles that originate in the Golgi (*43*), different types of unconventional secretion have also been described recently. The term unconventional secretion is used to describe extracellular release of proteins, independently of ER-Golgi trafficking, through plasma membrane docking of different types of cytosolic organelles (i.e., LE/MVBs, lysosomes, and APGs) (*44*). Growing evidence supports that autophagic cargo can sometimes be released extracellularly either as part of unconventional secretion or to cope with defective intracellular degradation (*12*, *45*–*47*). To check whether secretion of extracellular vesicles (ECVs) was affected by changes in SKP1 phosphorylation, the culture media of SKP1^WT^ and SKP1^T131A^ cells were subjected to serial differential ultracentrifugation to recover large ECVs (at 10,000*g*), small ECVs (at 100,000*g*), and soluble proteins precipitated from the remaining fraction. The presence of small ECVs in the 100,000*g* fraction was validated by immunoblot with multiple antibodies to ECV markers (Fig. 5A) and transmission EM (Fig. 5B). One subclass of ECVs directly related to LE/MVBs is exosomes, which are generated from the extracellular release of LE/MVB intraluminal vesicles upon fusion of these organelles with the plasma membrane (*48*). To determine the contribution of exosomes to our isolated ECV fractions, we performed whole-mount immune-EM for the exosome marker CD63 and found that the number of exosomes (CD63^+^ ECVs) was increased in the medium from SKP1^T131A^ cells compared to SKP1^WT^ cells (Fig. 5, C and D). Immunoblotting for the exosome markers HSP70, TSG101, and CD63 demonstrated a reduced amount of ECVs in SKP1^WT^ cells when deprived of nutrients, whereas we detected a marked increase of these exosome markers in the medium from SKP1^T131A^ cells independently of nutrient status (Fig. 5E). This was not a consequence of elevated intracellular levels of these proteins, which remained unchanged (Fig. 5E). In our experimental conditions, LC3B-II was also detected in ECVs, but only in those collected from SKP1^T131A^-expressing cells (Fig. 5E), suggesting possible release of APG or amphisome cargo from these cells, which has been observed in other contexts (*49*, *50*).

To further characterize SKP1-induced changes in ECVs, we used nanoflow cytometry (NanoFCM) analysis, which allows for the precise measurement of ECV number and size. The average size of ECVs detected in both SKP1^WT^ and SKP1^T131A^ cells was consistent with that of exosomes (~30 to 100 nm) (fig. S9, A and B). Particle count confirmed a significant increase in the number of ECVs released by SKP1^T131A^ cells (Fig. 5F), with only a minor decrease in average particle size (fig. S9B). Conversely, when SKP1 phosphorylation was increased by treating SKP1^WT^ cells with siRNA to PPM1B (si-PPM1B) (Fig. 1I), NanoFCM analysis showed a significant reduction in the number of ECVs released (Fig. 5G and fig. S9C), with no significant changes in average ECV size (fig. S9D). Once serum was removed from the culture medium (a condition in which SKP1 becomes hyperphosphorylated), there were no differences in the quantity of ECVs released between control and si-PPM1B cells (Fig. 5G), supporting the notion that nutrient deprivation–induced phosphorylation of SKP1 instead promotes degradation. Similar results were obtained by immunoblot analysis, where treatment with si-PPM1B resulted in reduced ECV markers in ECVs isolated from SKP1^WT^ cells (Fig. 5H).

The differences in abundance of specific proteins between ECVs of SKP1^WT^ and SKP1^T131A^ cells (i.e., LC3B-II in Fig. 5E) motivated us to determine possible changes in ECV content depending on SKP1 phosphorylation. To this effect, we performed MS analysis and comparative proteomics of ECVs from SKP1^WT^ and SKP1^T131A^ cells (Fig. 5I and table S7). We found a higher number of proteins displaying increased abundance (≥0.321 log_2_ fold change) in SKP1^T131A^ cells (127 proteins, right on the volcano plot in Fig. 5I) compared to SKP1^WT^ cells (16 proteins, left on the volcano plot in Fig. 5I). STRING analysis and gene ontology (GO) of proteins more abundant or only present in ECVs from SKP1^T131A^ cells revealed enrichment of proteins involved in cell adhesion molecule binding, microtubule binding, Golgi to plasma membrane transport, and carboxypeptidases (Fig. 5I and table S7). These proteins may be of interest in further studies characterizing a possible cargo-sorting effect of SKP1 phosphorylation on ECV contents. Overall, these findings support a role for SKP1 dephosphorylation in unconventional secretion.

### p^T131^-SKP1 regulates the switch between autophagy and unconventional secretion

**Fig. 6.**
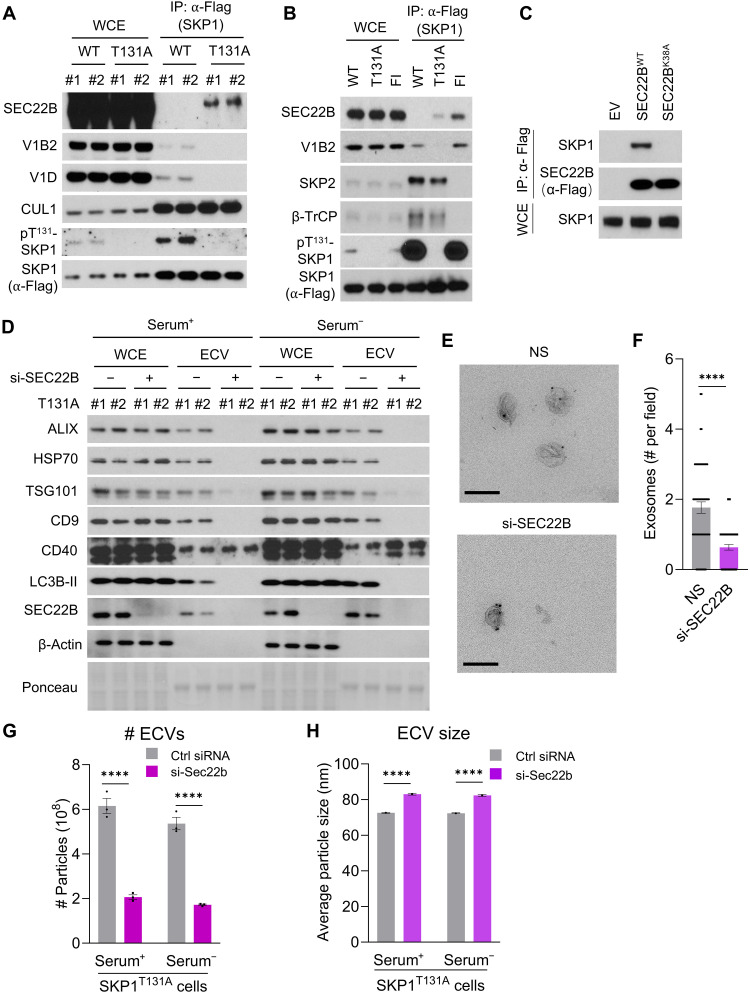
SEC22B mediates SKP1-triggered protein secretion. (**A** and **B**) Immunoblot of proteins from Flag co-IP in HeLa cells expressing Flag-tagged SKP1^WT^ or SKP1^T131A^ (A) or Expi293F cells transfected with Flag-tagged SKP1^WT^, SKP1^T131A^, or SKP1^F139A/I141A^ (FI) (B). WCE controls are shown in the left lanes. Co-IPs in (A) and (B) were performed after treatment with the DSS cross-linking agent (1 mM) for 1 hour. (**C**) Immunoblot of proteins from Flag co-IP in HeLa cells expressing empty vector control (EV) or Flag-tagged wild-type (WT) SEC22B (SEC22B^WT^) or a form of SEC22B that cannot bind SKP1 (SEC22B^K38A^). (**D**) Immunoblot of WCE and ECVs obtained from the culture medium of HeLa cells expressing SKP1^T131A^ after treatment with a control siRNA (−) or si-SEC22B (+), and cultured in DMEM supplemented (Serum^+^) or not (Serum^−^) with serum for 16 hours. Ponceau shows different electrophoretic pattern of WCE and ECV fractions. (**E** and **F**) Representative images from immune-EM with an antibody against CD63 (E) and quantification (F) of CD63^+^ vesicles in the small ECV fraction from HeLa cells expressing SKP1^T131A^ after treatment with a control siRNA (NS) or si-SEC22B. Scale bars, 200 μm; *n* = 60 images. (**G** and **H**) Number of secreted vesicles (G) and ECV size (H) based on NanoFCM analysis from ECVs isolated from culture medium of SKP1^T131A^ cells treated with control siRNA (Ctrl) or si-SEC22B cultured in DMEM supplemented (+) or not (−) with serum for 16 hours. *n* = 3 independent experiments with >4000 vesicles per condition. Representative histograms are shown in fig. S9E. Each experiment was performed a minimum of three times with similar results. Data are means ± SEM and individual values. Unpaired *t* test (F) or two-way ANOVA with Bonferroni’s multiple comparisons post hoc test [(G) and (H)] was used. Difference was significant for ****P < 0.0001.

To identify molecular mediators of the regulatory role of pT^131^-SKP1 on unconventional secretion, we set to copurify transient binding partners of SKP1 using cross-linking MS (XL-MS) in cells expressing Flag-SKP1^FI^. This mutant shows decreased binding to F-box proteins (Fig. 2A), facilitating detection of lower stoichiometric transient binding partners of SKP1 by XL-MS. After 1 hour of treatment with disuccinimidyl suberate (DSS), a noncleavable and membrane-permeable cross-linker, using IP followed by immunoblotting, we detected cross-linked complexes between SKP1^FI^ and V_1_D, but not SKP1^FI^ and the F-box protein SKP2 (fig. S10A). Moreover, XL-MS identified 13 cross-linked peptides of SKP1 and SEC22B (fig. S10B and table S8), a soluble *N*-ethylmaleimide–sensitive factor attachment protein receptor (SNARE) protein recently shown to play a role in the unconventional secretion of APG cargo (*51*, *52*). Co-IP experiments in cells expressing Flag-tagged forms of SKP1 revealed preferential binding of SEC22B to SKP1^T131A^ (Fig. 6, A and B), suggesting that the interaction between these two proteins occurs when SKP1 is not phosphorylated. This interaction does not require SKP1 interaction with F-box proteins, as SEC22B was still coimmunoprecipitated with Flag-tagged SKP1^FI^, which is unable to interact with F-box proteins. (Fig. 6B). The SKP1 peptide cross-linked to SEC22B includes Thr^131^ (fig. S10B), indicating that this region of SKP1 directly binds SEC22B. Since the interaction of SEC22B with SKP1 is mostly detected with SKP1^T131A^ (Fig. 6, A and B), phosphorylation at the Thr^131^ site may inhibit the interaction between SKP1 and SEC22B. In the case of SEC22B, the XL-MS experiment also yielded the amino acid sequence in this protein cross-linked to SKP1 (table S8), which we confirmed to be the region required for interaction, because a single amino acid mutation at this site (SEC22B^K38A^) resulted in failure to coimmunoprecipitate SKP1 with SEC22B (Fig. 6C).

SEC22B was found in the LE/MVB fraction independently of nutrient conditions [Dulbecco’s modified Eagle’s medium (DMEM) versus EBSS], and similar to p^T131^-SKP1, it was almost undetectable in the lysosome fraction (fig. S11A). Consistent with the described role of SEC22B in mediating unconventional secretion of LE/MVB and amphisome cargo, we found that LE/MVB-associated SEC22B was present on the surface of these organelles, as it was accessible to degradation by exogenously added trypsin (fig. S11B, top). In contrast, the small amount of SEC22B detected in lysosomes was resistant to trypsin degradation until the membrane of these organelles was disrupted by the addition of detergent (fig. S11B, bottom), suggesting that SEC22B is mostly present in the lysosome lumen and not on the surface. We confirmed that the presence of SEC22B in the lysosomal lumen was related to its degradation in this compartment, as the protein accumulated in lysosomes of mice upon injection with the protease inhibitor leupeptin (fig. S11C).

Since (i) impairment in SKP1 phosphorylation leads to an increase in exosome secretion (Fig. 5), (ii) SEC22B interacts with unphosphorylated SKP1 (Fig. 6, A and B), and (iii) both SEC22B and SKP1 are detected on the surface of LE/MVBs (fig. S11B), we propose that the role of SKP1 in unconventional secretion depends on SEC22B. In agreement with this hypothesis, we found that silencing of SEC22B notably decreases both exosome markers and LC3B in the medium from SKP1^T131A^ cells (Fig. 6D), the number of exosomes (CD63^+^ vesicles) secreted from SKP1^T131A^ cells (Fig. 6, E and F), and the number of total ECVs released by SKP1^T131A^ cells (Fig. 6, G and H, and fig. S9E).

The posttranslational modification status of SKP1 appears to play a substantial role in its ability to interact with SEC22B, as indicated by the fact that only unphosphorylated SKP1 interacted with SEC22B (Fig. 6A). However, for SKP1 to be dephosphorylated, it first becomes SUMOylated (Fig. 1). To determine whether SUMOylated SKP1 can interact with SEC22B, we performed affinity purification for streptavidin-tagged SKP1 and found that, while SUMOylated SKP1 could still interact with SEC22B, the interaction was notably weaker than in the absence of SUMOylation (fig. S12A). This suggests that SKP1 SUMOylation is an intermediate step needed for SKP1 dephosphorylation and that SUMOylated SKP1 is not the form of SKP1 preferentially interacting with SEC22B.

To further investigate the role of the SKP1-SEC22B interaction in the release of ECV cargo, we created cell lines with endogenous SEC22B knocked down and replaced with constructs containing either SEC22B^WT^ or SEC22B^K38A^ (fig. S12B), the latter of which lacks the ability to interact with SKP1 (Fig. 6C). We found that expressing the SEC22B^WT^ construct, but not the SEC22B^K38A^ construct, was sufficient to restore ECV secretion to levels comparable to cells with endogenous SEC22B expression in both nutrient-rich (Serum^+^) and nutrient-poor (Serum^−^) conditions (the abundance of ECV markers is shown in the immunoblots in fig. S12C). Levels of LC3B released in the ECV fraction were also restored by the expression of SEC22B^WT^, but not that of SEC22B^K38A^ (fig. S12C). The inability of SEC22B^K38A^ to rescue extracellular secretion in SEC22B knockdown cells suggests that the interaction with SKP1 is important in SEC22B-mediated secretion of ECVs.

Together, these results suggest that unphosphorylated SKP1 binds SEC22B to boost unconventional secretion of amphisome cargo. We propose that the dependence of SKP1 phosphorylation on the nutritional state allows this protein to control the degradation and secretory capacity of the cell in response to nutrient availability.

## DISCUSSION

**Fig. 7.**
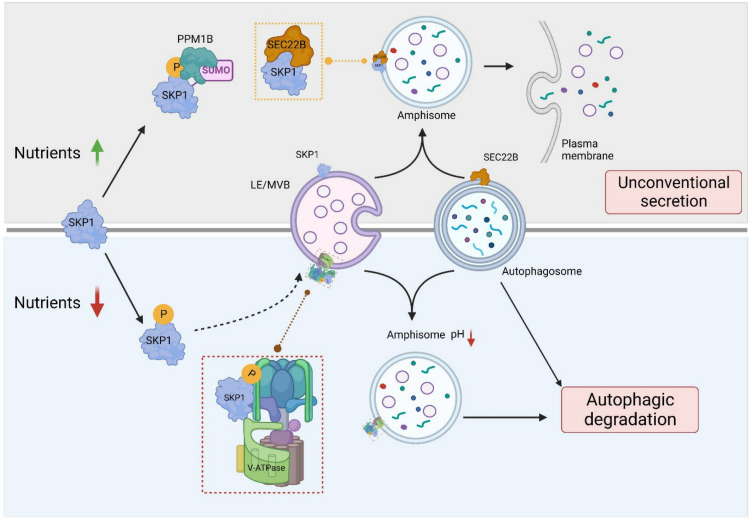
SKP1 phosphorylation regulates the starvation-induced switch between unconventional secretion and degradation by autophagy. Proposed working model for the effect of SKP1 phosphorylation on the fate of autophagic cargo. (**Top**) Under nutrient-rich conditions, SUMOylation of SKP1 promotes its association with and dephosphorylation by PPM1B. Unphosphorylated SKP1 binds SEC22B on the LE/MVB membrane and promotes extracellular release of autophagic content delivered to these compartments upon LE/MVB fusion with APGs to form amphisomes. (**Bottom**) Upon nutrient deprivation, phosphorylation of SKP1 present in LE/MVBs promotes its binding to and assembly of V-ATPase subunits to increase LE/MVB acidification. The lower pH of amphisomes contributes to increased autophagic degradation under these conditions.

Our work identifies a previously unknown, noncanonical function of SKP1, a well-studied assembly factor of >70 SCF/CRL1 ubiquitin ligases. Specifically, we provide evidence that changes in SKP1 phosphorylation modulate protein degradation in and secretion from the endolysosomal compartment (Fig. 7) in an SCF/CRL1-independent manner. In nutrient-poor conditions, a subpopulation of SKP1 becomes phosphorylated on Thr^131^ and localizes at LE/MVBs, augmenting their acidification by interacting with V_1_ domain subunits of the V-ATPase and promoting their assembly with V_0_ subunits. We propose that this acidification of LE/MVBs contributes to the well-characterized increase in macroautophagy rates upon nutrient deprivation (Fig. 7, bottom). Whereas the LE/MVB-associated and phosphorylated SKP1 promotes cargo degradation, the LE/MVB-associated and unphosphorylated SKP1 promotes secretion of the amphisome cargo (Fig. 7, top). We found that this secretion is mediated by SEC22B, a SNARE protein recently described as an effector in unconventional secretion (*51*, *52*), and that SEC22B only binds to SKP1 when unphosphorylated.

Although amphisomes have been described only as intermediate compartments in the maturation of APGs before full degradation in lysosomes (*39*), our work may support a role of amphisomes in autophagic flux during starvation due to the phospho-SKP1–dependent changes in the acidification of LE/MVBs. Whether cargo degradation necessarily requires the fusion of amphisomes with lysosomes or whether the SKP1-mediated increased assembly of the proton pump at LE/MVBs allows amphisomes to acquire an acidic enough pH to become efficient degradative compartments will require further study. Although the accumulation of amphisomes in cells expressing phosphorylation-incompetent SKP1 could originate in part because of reduced fusion of amphisomes with lysosomes for their clearance, we favor the hypothesis that amphisomes accumulate in these conditions due to their inability to acidify and degrade their cargo because (i) most phosphorylated SKP1 is present at the surface of LE/MVBs and not in lysosomes, (ii) p^T131^-SKP1 promotes the localization of an assembled V-ATPase at LE/MVBs, and (iii) LE/MVBs isolated from SKP1^T131A^-expressing cells display less acidic basal pH and inability to acidify in response to nutrient deprivation.

Our finding on the SKP1-dependent acidification of LE/MVBs also has a direct impact on the interpretation of results from the broadly used autophagy fluorescence tandem reporter assay. As proposed before, LC3B^+^ neutral pH compartments (RFP^+^GFP^+^ puncta) encompass both APGs and amphisomes (which contain LC3B contributed by the APG), which do not reach the acidification required for quenching the GFP fluorescence of the tandem reporter. However, in light of our findings, LC3B^+^ acidic compartments (RFP^+^GFP^−^ puncta), previously thought to result solely from APG or amphisome fusion with lysosomes, will also include amphisomes due to LE/MVB acidification. The up-regulating effect of SKP1 phosphorylation on autophagic activity is most noticeable during starvation, when APG biogenesis increases. As a side note, we noted that the effect of SKP1 phosphorylation on macroautophagy was more pronounced when using the tandem fluorescent reporter than by immunoblot for LC3B-II flux. The differences between both assays may point to differences in the overall amount of LC3B present in amphisomes when compared to APGs. If amphisomes have lower LC3B abundance, their fusion with lysosomes will result in less LC3B being degraded than when APGs fuse with lysosomes, thus attenuating genotype differences if the readout is LC3B abundance (immunoblot) but not with the fluorescent reporter that highlights amphisomes and APGs independently of their relative LC3B-II abundance.

The role of SEC22B in extracellular secretion has been described (*53*) and demonstrated to occur through interaction with SNARE proteins (*51*). Here, we found that SEC22B interaction with SKP1 modulates its role in extracellular secretion and identified the specific region in SEC22B interacting with SKP1. Further work will be required to characterize the interaction between SEC22B and SKP1 in the amphisome compartment in more detail. Current approaches are limited by the lack of reliable procedures to isolate a fraction of amphisomes of the purity required for this type of studies and by the need to use a large combination of fluorophores (three individual channels for amphisomes alone) when doing colocalization studies by immunofluorescence; development of additional techniques would be required before pursuing this line of inquiry.

This work also provides evidence about how SUMOylation of only a small fraction of a protein could have an outsized, and long-lasting, effect on the function of that protein, a phenomenon that has been defined as the SUMO enigma (*19*–*21*). We show here that SUMOylation of SKP1 promotes its binding to and dephosphorylation by the PPM1B phosphatase. Moreover, SKP1 is rapidly and transiently SUMOylated upon nutrient repletion, and this correlates with its dephosphorylation by PPM1B. The tight regulation of p^T131^-SKP1 seems necessary to the titration of macroautophagy degradation by regulating V-ATPase assembly. Although we have found that most p^T131^-SKP1 is associated with LE/MVBs, future studies will be needed to determine whether SUMOylation/dephosphorylation of SKP1 occurs at the surface of this organelle or, instead, whether SUMOylation promotes detachment of p^T131^-SKP1 from the LE/MVB and subsequent rapid dephosphorylation in the cytoplasm. Of note, we found that cells expressing either SKP1^RR^ (which does not bind PPM1B and therefore is more phosphorylated) or SKP1^T131E^ (which mimics phosphorylation) do not survive upon starvation. Thus, it appears that phosphorylation of SKP1 after nutrient deprivation needs to be transient and only in a subpopulation of cellular SKP1 (the fraction associated with LE/MVBs) to favor appropriate cellular fitness.

Although once considered an exclusively degradative process, it is now acknowledged that autophagy also has important regulatory roles, being able to finely tune the degradation of specific substrates according to cellular needs. Unconventional secretion of autophagic cargo has previously been described in contexts where intracellular proteolysis is impaired (*41*, *54*–*56*) and has been proposed to be a last resort to rid the cell of toxic waste products that accumulate when autophagic degradation is blocked (*12*). In addition to its role in secretion of undegraded autophagic cargo, unconventional secretion pathways have been described in various physiologic contexts in basal conditions for processes including cell-to-cell communication (*8*).

Further work will be required to fully understand the physiological importance of the SKP1 phosphorylation–dependent switch between autophagic degradation and secretion. Among the different possibilities, the tight connection of SKP1 with the cellular nutritional status provides support for posttranslational modifications in this protein serving as a mechanism for cells to modulate their own energetic balance. Thus, by promoting protein degradation of proteins otherwise destined for secretion, cells can increase the pool of free amino acids in conditions of nutrient scarcity to sustain biosynthetic functions and use them as an alternative source of energy (*57*). Furthermore, on the basis of the quantitative and qualitative changes in ECV cargo observed upon changes in SKP1 phosphorylation state, we propose that this could be a mechanism to modulate cell-to-cell communication and ensure a coordinated tissue-wide response to environmental change. Added to the previously known ability of SKP1 to regulate proteasome degradation [through SCF/CRL1-mediated substrate ubiquitylation (*13*)], this study also supports a key role for this protein in modulating two additional cellular proteostasis mechanisms, autophagic degradation (through V-ATPase–mediated amphisome acidification) and protein secretion (through SEC22B interaction). This ability of a single protein, especially one as evolutionarily conserved as SKP1, to coordinate multiple aspects of cellular proteostasis through discrete posttranslational modifications could allow for the mounting of a rapid stress response under proteotoxic stress conditions. Thus, SKP1 is a potential target in pathological conditions related to proteostasis failure (i.e., protein conformational disorders), where a shift from intracellular degradation to extracellular secretion plays an important role in disease pathogenesis and progression (*45*, *58*, *59*). Together, our findings present a molecular mechanism dictating the fate of autophagic cargo (degradation versus unconventional secretion) based on the nutrient status of the cell.

## MATERIALS AND METHODS

### Animal models

All animal handling and treatments in this study were done according to protocol, and all animal studies were under an animal study protocol approved by the Institutional Animal Care and Use Committee of the Albert Einstein College of Medicine. Animals were maintained at 19° to 23°C in a 12-hour light/dark cycle and fed ad libitum, except where indicated. Rodents deprived of food were given ad libitum access to water. Adult (3 to 6 months) male Wistar rats (Charles River Laboratories) and adult (3 to 6 months) male mice (The Jackson Laboratory) used for this study were maintained under pathogen-free conditions in ventilated cages with no more than two rats per cage or four mice per cage.

### Cell culture and treatments

HEK293T, HeLa, and RPE1 cells were cultured in DMEM supplemented with 10% fetal bovine serum (FBS) and penicillin-streptomycin/l-glutamine. Where indicated, cells were treated with a combination of endolysosomal protease inhibitors [20 mM ammonium chloride (Sigma-Aldrich) and 100 μM leupeptin (Thermo Fisher Scientific); + ammonium chloride and leupeptin (N/L) in text] or 100 nM bafilomycin A1 (Santa Cruz Biotechnology), 100 nM concanamycin A (MedChem Express), 0.5 μM KM91104 (MedChem Express), 0.5 μM apillimod (MedChem Express), and 40 μM choloroquine (MedChem Express). Starvation experiments were performed after washing cells several times with phosphate-buffered saline (PBS) and adding either EBSS (Gibco) or DMEM without FBS supplementation. HEK293T cells were transfected with plasmids with polyethylenimine (PEI). RPE1 and HeLa cells were transfected with plasmids with Lipofectamine 3000. All siRNA oligos were transfected with Lipofectamine RNAiMAX. To generate HeLa cells expressing SKP1^WT^, SKP1^RR^, or SKP1^T131A^ only, we first generated HeLa cell lines stably expressing Flag-tagged SKP1^WT^, SKP1^RR^, or SKP1^T131A^ with pBabe vector and then designed single-guide RNA (sgRNA) target sequences to knock out endogenous SKP1.

HEK293T cells were transiently cotransfected with retroviral vector (pBabe-puro) containing vesicular stomatitis virus G protein (VSV-G) and SKP1^WT^, SKP1^RR^, or SKP1^T131A^ along with pUMVC using PEI. pBABE-puro was a gift from H. Land, J. Morgenstern, and B. Weinberg (Addgene plasmid no. 1764). After transfection for 48 hours, retrovirus-containing medium was collected and supplemented with polybrene (8 mg/ml; Sigma-Aldrich). HeLa cells were infected by replacing the cell culture medium with the viral supernatant for 24 hours. Selection of stable clones was carried out using puromycin (1 mg/ml) for 7 days.

The sgRNA sequences that target endogenous SKP1 were cloned into pSpCas9(BB)-2A-GFP (PX458), a gift from F. Zhang (Addgene plasmid no. 48138) (*60*). HeLa cells were seeded into 10-cm dishes at 70% confluency and transfected with 10 mg of the appropriate sgRNA-containing PX458 plasmid using Lipofectamine 3000 (Life Technologies). The transfection was performed according to the manufacturer’s recommended protocol, using a 2.5:1 ratio of Lipofectamine/DNA. Two days after transfection, GFP-positive cells were sorted using the Beckman Coulter MoFlo XDP cell sorter (100-μm nozzle) to a 96-well plate with 1 cell per well. The positive clones were validated by immunoblotting.

### Antibodies

The dilutions and sources of antibodies used for immunoblot, IP, and immunogold EM in this study can be found in the Key Reagents Table. All antibodies used were validated following the multiple dilution method and, where available, using cell lines or tissues from animals knock out for the antigen.

### Protein electrophoresis and immunoblotting

Protein concentration was determined using the Lowry method (*61*) with BSA as the standard. Immunoblotting was performed after transferring SDS–polyacrylamide gel electrophoresis (SDS-PAGE) gels to nitrocellulose membrane and blocking with 5% milk in 0.01% Tween–tris-buffered saline for 1 hour at room temperature (RT). The proteins of interest were visualized after incubation with primaries by chemiluminescence using horseradish peroxidase–conjugated secondary antibodies in the LAS-3000 Imaging System (Fujifilm, Tokyo, Japan) or SRX-101A Tabletop X-Ray Film Processor (Konica). Densitometric quantification of the membranes was performed using ImageJ [National Institutes of Health (NIH); (*62*)]. All protein quantifications were done after normalization of protein levels to a loading control (tubulin or Ponceau S staining). When the number of experimental conditions exceeded the number of lanes available in the gel, all gels for the same experiment included one lane with the same sample, which was used to normalize samples across gels after densitometric quantification. Each immunoblot was performed a minimum of three times with similar results, with one representative blot shown in each figure.

### Affinity purification

HEK293T cells were transiently transfected with DNA using PEI (Polysciences). After transfection for 24 hours, cell lysis was carried out with lysis buffer [50 mM tris (pH 7.4), 150 mM NaCl, 10% glycerol, 0.3% Triton X-100, and 0.1% NP-40] supplemented with protease and phosphatase inhibitors. For the affinity purifications shown in Fig. 2A and table S6, a modified lysis buffer was used [50 mM MES (pH 5.9), 150 mM NaCl, 10% glycerol, 0.3% Triton X-100, and 0.1% NP-40]. Lysates were then immunoprecipitated with anti-FLAG antibody conjugated to agarose. After washing with lysis buffer for four times, elution was carried out with 3× FLAG peptide. For endogenous IP, lysates were incubated with anti–SUMO-1 or anti-V1B2 antibody and rotated for 3 hours at 4°C. Then, protein G beads were added and incubated for 1 hour at 4°C. After washing with lysis buffer for four times, the beads were denatured with 1× lithium dodecyl sulfate (LDS) for 3 min at 95°C. For affinity purification with MagStrep “type3” XT beads, elution was performed with Strep-TactinXT elution buffer. For denaturing IP, cells were lysed with 2% SDS and denatured for 5 min at 95°C, and then the lysates were diluted 1:20 to perform affinity purification.

### Isolation of subcellular compartments

Rodent liver lysosomes and LE/MVBs were isolated after tissue homogenization and centrifugation in density gradients. For lysosomes, homogenates underwent differential centrifugation to obtain a light mitochondrial and lysosome fraction, which was then subjected to ultracentrifugation in a discontinuous metrizamide density gradient at 141,000*g* for 1 hour at 4°C to separate lysosomes active (+) and inactive (−) for chaperone-mediated autophagy, as described previously (*35*). LE/MVBs were isolated from rodent liver homogenate, as described previously (*63*), by ultracentrifugation in consecutive discontinuous density gradients of Percoll (GE Healthcare) diluted in sucrose (27% Percoll followed by 10% Percoll, respectively). Both centrifugation steps were performed at 34,000*g* for 1 hour at 4°C. Integrity of LE/MVB and lysosome fractions was determined by measuring enzymatic β-hexosaminidase activity in pelleted organelles compared to the extra-organelle medium (*64*). Cytosol was obtained by centrifuging the postnuclear supernatant obtained during the LE/MVB isolation protocol at 100,000*g* for 1 hour at 4°C and taking the supernatant. Before freezing, all subcellular fractions were supplemented with a protease inhibitor cocktail [10 mM leupeptin, 10 mM 4-(2-aminoethyl) benzenesulfonyl fluoride hydrochloride, 1 mM pepstatin, and 100 mM EDTA] and phosphatase inhibitor cocktails 2 (Sigma-Aldrich, P5726) and 3 (Sigma-Aldrich, P0044).

LE/MVBs were isolated from cells using a modification of a previously described technique (*65*). Cells (2 × 10^8^) were grown to confluence in full DMEM and were incubated for an additional 1 hour either in full DMEM or in EBSS to deprive cells of nutrients after several washes with PBS. Following the incubation, cells were gently scraped from the plate, pelleted, and washed repeatedly in PBS before resuspension in PBS containing 0.25 M sucrose and 20 mM Hepes (pH 7.4). Resuspended cells were broken using nitrogen cavitation and subsequent manual dounce homogenization of the cavitated sample. This homogenate was centrifuged at 2000*g* for 5 min, and the resulting supernatant was used for consecutive discontinuous density gradient ultracentrifugation in Percoll and sucrose as described above for LE/MVB isolation from rodent liver tissue.

### Isolation of ECVs

Forty-eight hours before isolation, 1 × 10^7^ HeLa cells were plated in 150-mm tissue culture dishes. Culture medium was removed and replaced with 20 ml of DMEM with exosome-depleted FBS (Thermo Fisher Scientific, catalog no. A2720801) and incubated for 48 hours. Then, cells were incubated with DMEM in the presence or absence of exosome-depleted FBS for an additional 16 hours. Conditioned medium was collected, transferred to 50-ml tubes, and centrifuged for 20 min at 2000*g* at 4°C. Supernatants were transferred to a fresh 50-ml tube and centrifuged for 30 min at 10,000*g* at 4°C. Supernatants were ultracentrifuged for 90 min at 35,000 rpm at 4°C in a Ti-90 rotor to pellet ECVs. To fix the samples for EM, 16% paraformaldehyde (PFA) was added to a final concentration of 2% PFA. When assessing by Western blot, normalization was performed by controlling for the number of cells seeded onto the plate.

### Nano-flow cytometry

The nFCM flow nanoanalyzer N30 was used to measure the concentration and size of particles following the manufacturer’s instructions and as described previously (*66*). Briefly, two single photon–counting avalanche photodiodes were used to detect individual particles’ side scatter (SSC) simultaneously. The instrument was calibrated separately for concentration and size using 250-nm silica beads of known particle concentration and a silica nanosphere cocktail mixture of known sizes to generate a standard curve. PBS was used as a background signal, which was removed from each sample. Measurements were taken at a sampling pressure of 1.0 kPa for a 1-min period using samples diluted to ensure that 2000 to 12,000 counts were recorded for each measurement. The laser was set to 10 mW and 10% SSC decay. Data were generated through the NanoFCM Professional Suite v1.8 software.

### pH measurements

#### Intact cells

Endolysosomal pH measurements in cells were performed as described (*67*). Cells were cultured in either full DMEM or EBSS cultured medium for 1 hour before incubating with 1 μM LysoSensor Yellow/Blue DND-160 diluted in either DMEM or EBSS for 5 min at 37°C, 5% CO_2_. After the incubation, cells were washed three times with PBS (pH 7.4), maintained in PBS while being analyzed in a microplate reader (Tecan Infinite 200 Pro) (485/530 nm), and kept at a temperature of 37°C. To generate a calibration curve, LysoSensor Yellow/Blue DND-160 was diluted to 1 μM in 25 mM MES calibration buffer (pH 4 to 7.5), containing 125 mM KCl and 25 mM NaCl. Fluorescence was measured using a microplate reader (340/440 nm and 380/530 nm) at 37°C.

#### Isolated LE/MVBs

Isolated LE/MVBs were incubated in 1 μM LysoSensor Yellow/Blue DND-160 diluted in 20 mM Mops and 0.3 M sucrose (pH 7.3) (Mops-sucrose) and incubated at 37°C for 5 min before pelleting at 25,000*g* for 5 min at 4°C and resuspended in Mops-sucrose. Fluorescence was measured in a microplate reader kept at 37°C, as described above for intact cells.

### Autophagy measurements in cultured cells

Macroautophagy activity was assessed in cells stably transduced with lentivirus containing the GFP-RFP-LC3B tandem reporter (*38*). After indicated treatments, cells were fixed with 4% PFA. For imaging, on the day of data collection, cells were incubated in FluoroBrite DMEM supplemented with 10% FBS, 25 mM Hepes, and sodium pyruvate. Imaging was performed using a DeltaVision Elite inverted microscope system (Applied Precision), using a 100 × UPLS Apo oil objective (numerical aperture 1.40) from Olympus. Excitation was achieved with a 7 Color Combined Insight solid state illumination system equipped with a polychroic beam splitter with filter sets to support fluorescein isothiocyanate (FITC) [excitation (ex): 475/28 nm, emission (em): 523/36 nm] and Cy5 (ex: 632/22 nm, em: 676/34 nm). Images were acquired using a CoolSNAP HQ2 camera. Images were quantified using ImageJ (NIH) as described previously (*38*). Macroautophagy flux was calculated as the change of RFP^+^/GFP^+^ double-positive puncta into RFP^+^/GFP^−^ puncta due to quenching of GFP at low pH. Nuclei were labeled with 4′,6-diamidino-2-phenylindole (DAPI).

For labeling of the endocytic compartment, cells were incubated with BSA–Alexa Fluor 647 (20 mg/ml, A34785, Thermo Fisher Scientific) at 37°C for 20 min, followed by washes with medium before fixation. Colocalization between three fluorophores (GFP-RFP-LC3B and BSA–Alexa Fluor 647) was measured using EzColocalization plugin in ImageJ (*68*). A threshold overlap score (TOS) value of −1 corresponds to the minimum possible overlap (anti-colocalization), 0 corresponds to the same overlap as would occur by chance (noncolocalization), and 1 corresponds to the maximum possible overlap (colocalization).

### Intracellular protein degradation

Degradation of long-lived proteins was measured in confluent cells by metabolic labeling with ^3^H-leucine (2 μCi/ml) for 48 hours at 37°C. After extensive washing, cells were kept in the medium containing an excess of unlabeled leucine (2.8 mM) to prevent the reutilization of radiolabeled leucine, supplemented with or without 10% FBS. Aliquots of medium taken at different times were precipitated with 20% tricarboxylic acid (TCA) and BSA (20 mg/ml), and proteolysis was measured as the percentage of acid-insoluble radioactivity (protein) transformed into acid-soluble radioactivity (amino acids and small peptides) at the end of the incubation. Total radioactivity incorporated into the cellular proteins was determined as the amount of acid-precipitable radioactivity in labeled cells immediately after washing. The fraction of intracellular degradation occurring in lysosomes was identified as that inhibited by N/L treatment.

### Protein topology in isolated organelles

The presence of specific proteins in the surface or lumen of LE/MVBs and lysosomes was determined by incubating isolated organelles with increasing concentrations of trypsin (Sigma-Aldrich, catalog no. T1426) in Mops-sucrose [20 mM Mops and 0.3 M sucrose (pH 7.3)] at RT for 15 min. The detergent Triton X-100 (0.1%) was added to a control sample to disrupt the membrane and expose all internalized proteins to trypsinization.

### Electron microscopy

All EM work was performed at Microscopy Laboratory of NYU Grossman School of Medicine. Briefly, for morphology of autophagic vesicle analysis, following procedures previously described in (*69*), cultured cells were fixed in 0.1 M sodium cacodylate buffer (pH 7.4) containing 2.5% glutaraldehyde and 2% PFA overnight at 4°C and postfixed with 1% osmium tetroxide mixed with 1% potassium ferrocyanide for 1 hour at 4°C, then block-stained in 0.25% aqueous uranyl acetate overnight at 4°C, processed in a standard manner, and embedded in EMbed 812 (Electron Microscopy Sciences, Hatfield, PA). Ultrathin sections (70 nm) were cut and mounted on 200-mesh copper grids. All EM grids were stained with uranyl acetate and lead citrate by standard methods, and examined under either Philips CM-12 electron microscope (FEI; Eindhoven, The Netherlands) and photographed with a Gatan (4k × 2.7k) digital camera or Talos L120C electron microscope (Thermo Fisher Scientific, Hillsboro, OR) coupled with Gatan 4k × 4k OneView Camera (Gatan Inc., Pleasanton, CA).

For analysis of exosome morphology, we placed 5 μl of isolated exosomes on glow-discharged carbon-coated 200-mesh copper grids and stained the samples with 1% uranyl acetate aqueous solution. For whole-mount immune-EM, we placed 5 μl of 2% PFA–fixed exosomes on glow-discharged formvar carbon–coated copper grids and allowed the samples to adsorb for 20 min. After washing with PBS, the grids were incubated with 50 mM glycine in PBS for 5 min, blocked with 1% cold-water fish skin gelatin (Sigma-Aldrich) for 10 min, and incubated with primary antibodies (anti-CD63, Abcam) in blocking solution for 2 hours at RT. After washing with PBS, gold-conjugated secondary antibodies (18-nm protein-A–gold, Cell Microscopy Center, University Medical Center Utrecht) were applied in the blocking buffer and incubated for 1 hour. Following washing with PBS, the grids were fixed in 1% glutaraldehyde in PBS for 5 min, washed with water, contrasted, and embedded in a mixture of 3% uranyl acetate and 2% methylcellulose at a ratio of 1:9. All stained grids were examined under a Philips CM-12 electron microscope and photographed with a Gatan (4k × 2.7k) digital camera (Gatan, Pleasanton, CA).

To quantify CD63-positive ECVs (exosomes), HeLa cells expressing SKP1^WT^ or SKP1^T131A^ were cultured on 12-mm glass coverslip and fixed with 2% PFA and 0.1% glutaraldehyde in PBS for 1 hour. Cells were then incubated with 50 mM glycine/PBS for 5 min, blocked in 1% BSA/PBS for 10 min, and incubated with anti-CD63 monoclonal antibody in 1% BSA/PBS for 2 hours at RT. A rabbit anti-mouse bridge antibody (1:50, Jackson ImmunoResearch Labs, West Grove, PA) was applied for overnight at 4°C. Cells were then washed with PBS before adding 18 nm of gold-conjugated goat anti-rabbit (Jackson ImmunoResearch Labs, West Grove, PA) for 30 min at RT. Cells were fixed with 2.5% glutaraldehyde in PBS for 30 min, postfixed with 1% OsO_4_/PBS, and then treated with 1% tannic acid/PBS for 30 min. Treatment with 1% OsO_4_/PBS followed by 1% tannic acid/PBS was repeated once more before cells were fixed again in 1% OsO_4_/PBS for 1 hour. Cells were dehydrated with serial ethanol dilutions and critical point–dried using a Tousimis Autosamdri-931 critical point dryer (Tousimis, Rockville, MD). A thin layer of carbon (~10 nm) was coated using BOC Edwards Auto 306 evaporator (BOC Edwards, Mississauga, Ontario, Canada), and imaging was performed using Zeiss Gemini300 SEM using back scatter detector and in-lens detector with 10 kV at a 5.8-mm walking distance.

### Mass spectrometry

Affinity-purified proteins were reduced with dithiothreitol (DTT) at 57°C for 1 hour (2 μl of 0.2 M). Samples were then alkylated with iodoacetamide at RT in the dark for 45 min (2 μl of 0.5 M) and for detergent and FLAG peptide removal loaded onto NuPAGE 4 to 12% bis-tris gel 1.0 mm (Life Technologies Corporation). The gel was ran either for approximately 5 min at 200 V or for 15 min, if overexpressed bait proteins were present (tables S2, S5, and S6), and excised and analyzed separately on the mass spectrometer to increase the dynamic range of the analysis. Samples within a study were always treated identical. The gel was stained using GelCode Blue Stain Reagent (Thermo Fisher Scientific), and Coomassie-stained gel plugs or bands were excised. The excised gel pieces were destained in 1:1 (v/v) solution of methanol and 100 mM ammonium bicarbonate solution using at least three exchanges of destaining solution. The destained gel pieces were partially dehydrated with an acetonitrile rinse and further dried in a SpeedVac concentrator for 20 min. Two hundred nanograms of sequencing grade–modified trypsin (Promega) was added to each gel sample. After the trypsin was absorbed, 250 μl of 100 mM ammonium bicarbonate was added to cover the gel pieces. Digestion proceeded overnight on a shaker at RT. A slurry of R2 20 μm Poros beads (Life Technologies Corporation) in 5% formic acid and 0.2% trifluoroacetic acid (TFA) was added to each sample at a volume equal to that of the ammonium bicarbonate added for digestion as described previously (*70*). The digested peptides were allowed to bind to the Poros beads for 3 hours with vigorous shaking at 4°C. The peptide loaded beads were transferred onto equilibrated C18 ziptips (Millipore) using a microcentrifuge for 30 s at 6000 rpm. Gel pieces were rinsed three times with 0.1% TFA, and each rinse was added to its corresponding ziptip followed by microcentrifugation. The beads were further washed with 0.5% acetic acid. Peptides were eluted by the addition of 40% acetonitrile in 0.5% acetic acid followed by the addition of 80% acetonitrile in 0.5% acetic acid. The organic solvent was removed using a SpeedVac concentrator, and the sample was reconstituted in 0.5% acetic acid. The desalted peptides were injected onto a C18 pre-column using the autosampler of an EASY nLC1000 (Thermo Fisher Scientific) connected to a PepMap C18 analytical column. Peptides were gradient-eluted into a Q Exactive mass spectrometer (Thermo Fisher Scientific) using a 1-hour gradient from 2% solvent B to 31% solvent B in 60 min, in 10 min to 40% B, and another 10 min to 100% solvent B. Solvent A consisted of 2% acetonitrile in 0.5% acetic acid and solvent B of 90% acetonitrile in 0.5% acetic acid. High-resolution full MS spectra were acquired with a resolution of 70,000, an automatic gain control (AGC) target of 1 × 10^6^, with a maximum ion time of 120 ms, and scan range of 400 to 1500 mass/charge ratio (*m*/*z*). Following each full MS, 20 data-dependent high-resolution higher-energy collisional dissociation (HCD) MS/MS spectra were acquired. All MS/MS spectra were collected using the following instrument parameters: resolution of 17,500, AGC target of 5 × 10^4^, maximum ion time of 120 ms, one microscan, 2 *m*/*z* isolation window, fixed first mass of 150 *m*/*z*, and normalized collision energy (NCE) of 27. The MS/MS spectra were searched against a UniProt human protein database with common laboratory contaminants, and the sequence of the tagged bait proteins was added using Sequest within Proteome Discoverer 1.4. The search parameters were as follows: mass accuracy better than 10 parts per million (ppm) for MS1 and 0.02 Da for MS2, two missed cleavages, fixed modification carbamidomethyl on cysteine, variable modification of oxidation on methionine, and deamidation on asparagine and glutamine. The data were filtered using a 1% false discovery rate (FDR) cutoff for peptides and proteins against a decoy database, and only proteins with at least two unique peptides were reported.

For the phosphorylation analysis of SKP1, the data were searched using the search engine Byonic against the same database described above, and in addition to the variable modifications listed above, variable modification of phosphorylation was enabled for serine, threonine, and tyrosine. The area under the curve for all forms of the modified and corresponding unmodified peptide was determined, and the % modified peptide of the total was reported.

For the ECV samples, the samples were digested using the S-trap (Protifi) following the manufacturer’s instruction. In brief, 25 μg of each sample was mixed with SDS solubilization buffer [5% SDS in 50 mM triethyl ammonium bicarbonate (TEAB) (pH 7.5)] for a total volume of 500 μl. The proteins were reduced with 2 μl of DTT (0.2 M) at 57°C for 1 hour followed by alkylation with 2 μl of iodoacetamide (0.5 M) for 45 min in the dark. The samples were acidified using 12% phosphoric acid solution, and S-trap buffer [90% methanol and 100 mM final TEAB (pH 7.1)] was added to the sample and loaded onto the S-trap. Samples were washed three times using 50 mM TEAB (pH 8) and subsequently digested with trypsin (1 μg per sample) for 1 hour at 47°C. The digested peptides were eluted using 40% acetonitrile and 0.5% acetic acid, followed by 80% acetonitrile and 0.5% acetic acid. The peptide elutions were dried using a SpeedVac concentrator, and the peptides were resuspended in 0.5% acetic acid and stored at −80°C until analysis by MS. The desalted peptides were injected onto a C18 pre-column using the autosampler of an EASY nLC1200 (Thermo Fisher Scientific) connected to a PepMap C18 analytical column. Peptides were gradient-eluted into an Orbitrap Eclipse mass spectrometer (Thermo Fisher Scientific) using a 1-hour gradient from 0 to 5% solvent B in 5 min, to 15% solvent B in 60 min, to 25% solvent B in 35 min, to 40% solvent B in 20 min, and to 100% solvent B in 10 min. Solvent A consisted of 2% acetonitrile in 0.5% acetic acid and solvent B of 90% acetonitrile in 0.5% acetic acid. High-resolution full MS spectra were acquired with a resolution of 240,000, an AGC target of 1 × 10^6^, with a maximum ion time of 50 ms, and scan range of 400 to 1500 *m*/*z*. Following each full MS, the top speed method was used to acquire HCD MS/MS spectra in the ion trap using rapid scan. All MS/MS spectra were collected using the following instrument parameters: 0.7 *m*/*z* isolation window (quadrupole isolation), AGC target of 2 × 10^4^, normalized AGC target of 200%, maximum ion time of 18 ms, one microscan, and NCE of 27. The MS/MS spectra were searched against a UniProt human protein database with common laboratory contaminants using Sequest within Proteome Discoverer 1.4. The search parameters were as follows: mass accuracy better than 10 ppm for MS1 and 0.2 Da for MS2, two missed cleavages, fixed modification carbamidomethyl on cysteine, variable modification of oxidation on methionine, and deamidation on asparagine and glutamine. The data were filtered using a 1% FDR cutoff for peptides and proteins against a decoy database, and only proteins with at least two unique peptides were reported.

### Cross-linking MS

Expi293F cells were grown in a flask at 37°C with agitation (100 rpm) until the cell concentration reached 1.5 million/ml. The cells were transfected with Flag-tagged SKP1^FI^ for 24 hours. Then, cells were washed with ice-cold PBS (pH 8.0) for three times and incubated with the DSS solution to a final concentration of 1 mM for 1 hour at RT. The reaction was quenched by additional tris-HCl (pH 7.5) to a final concentration of 20 mM for 15 min at RT. After centrifugation at 300*g* for 5 min, the supernatant was removed and cell pellet was lysed on ice and immunoprecipitated with anti-FLAG antibody conjugated to agarose. Elution was carried out with 3× FLAG peptide. The samples were separated with SDS-PAGE followed with Coomassie brilliant blue stain. Stained gel was split into five slices covering molecular weights between 50 and 70, 70 and 80, 80 and 120, 120 and 150, and 150 and 250 kDa. Each gel slice was further diced and treated with 5 mM DTT followed by 25 mM iodoacetamide (*71*). Digestion reactions (50 μl per sample) containing 50 mM NH_4_HCO_3_ and SOLu-trypsin (20 ng/μl; Sigma-Aldrich) were incubated at 37°C overnight with shaking (1200 rpm). After incubation, reactions were quenched by mixing with equal volume of 2% heptafluorobutyric acid. Peptides from supernatants were desalted on C18 spin tips (Pierce), dried under vacuum, and redissolved in 20 μl 0.1% formic acid. Peptides were analyzed on Orbitrap Lumos Fusion mass spectrometer coupled with Dionex Ultimate 3000 UHPLC (Thermo Fisher Scientific). Peptides were resolved on 50-cm EASY-spray column (Thermo Fisher Scientific) over 90-min-long gradient from 4 to 40% acetonitrile in 0.1% formic acid at a flow rate of 0.25 μl/min. Data-dependent acquisition method for peptide identification was set up as in (*72*), except that each cycle was set to last 2 s. Data-dependent acquisition method for identification of cross-linked peptides was described elsewhere (*73*). Raw data files were analyzed in Proteome Discoverer 2.1 (Thermo Fisher Scientific) to identify peptides and proteins in the sample. Protein database for Sequest HT search engine contained *Homo sapiens* proteome and list of common protein contaminants. Fully specific tryptic peptides with maximum two missing cleavages were allowed. The list of variable modifications included carbamidomethylation of cysteine, methionine oxidation, mono- and dimethylation of lysine and arginine, acetylation of protein N terminus, and phosphorylation of tyrosine, serine, and threonine. All remaining parameters were left with default settings. The resulting list of identified proteins was filtered at 1% FDR and used to create focused database for identification of cross-linked peptides. Cross-linked peptides were identified using pLink2 (*74*). Maximum four missed trypsin cleavages were allowed; fixed modification was carbamidomethylation of cysteine and variable oxidation of methionine; cross-linker was DSS; and FDR level was set to 1%. Other parameters were left unchanged.

### Quantification and statistical analysis

All data presented are means ± SEM and individual values. Before statistical testing, normality was assessed using the Shapiro Wilk test. Statistical significance was compared by two-tailed unpaired Student’s *t* test for two groups, one-way analysis of variance (ANOVA) for a single parameter in multiple groups, or two-way ANOVA for multiple parameters in multiple groups. The post hoc test used for multiple comparisons is stated in the figure legends. The number of animals used per experiment was calculated through power analysis based on previous results. Statistical analyses were performed in GraphPad Prism 9.0.

## Supplementary Materials

### This PDF file includes:

Figs. S1 to S12 Legends for tables S1 to S8 Legends for key table for mass spectrometry raw files 1 and 2 Legend for raw data table Table S9 Uncropped membranes

### Other Supplementary Material for this manuscript includes the following:

Tables S1 to S8

Key table for mass spectrometry raw files 1 and 2

Raw data table
