# Supplementary material for: A noncanonical function of SKP1 regulates the switch between autophagy and unconventional secretion
Source: Sci Adv. 2023 Oct 13;9(41):eadh1134. doi: 10.1126/sciadv.adh1134 (PMC10575587; doi:10.1126/sciadv.adh1134)
Supplement: Supplementary file 1 — Figs. S1 to S12 Legends for tables S1 to S8 Legends for key table for mass spectrometry raw files 1 and 2 Legend for raw data table Table S9 Uncropped membranes [file sciadv.adh1134_sm.pdf]

Science Advances
AAAS

Supplementary Materials for

A noncanonical function of SKP1 regulates the switch between autophagy and unconventional secretion

Jie Li *et al.*

Corresponding author: Ana Maria Cuervo, ana-maria.cuervo@einsteinmed.edu;
Michele Pagano, michele.pagano@nyulangone.org

Sci. Adv. **9**, eadh1134 (2023)
DOI: 10.1126/sciadv.adh1134

The PDF file includes:

Figs. S1 to S12
Legends for tables S1 to S8
Legends for key table for mass spectrometry raw files 1 and 2
Legend for raw data table
Table S9
Uncropped membranes

Other Supplementary Material for this manuscript includes the following:

Tables S1 to S8
Key table for mass spectrometry raw files 1 and 2
Raw data table

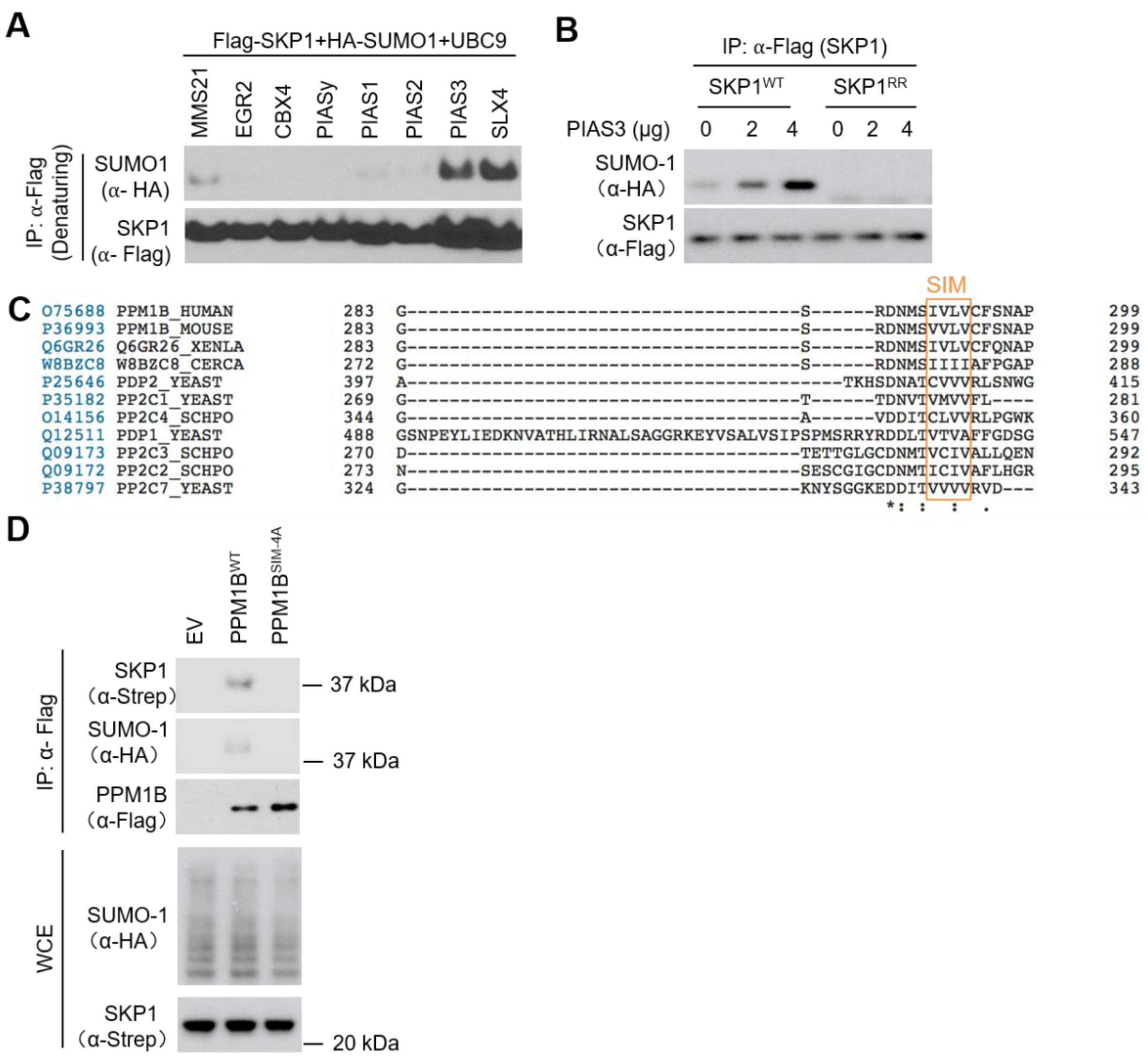

Figure S1. SUMOylated SKP1 binds PPM1B.
(**A**) Immunoblot for the indicated proteins after immunoprecipitation (IP) in denaturing conditions of HeLa cells expressing Flag-SKP1, HA-SUMO1 and Myc-UBC9 with co-transfection of the indicated SUMO E3 ligase plasmids.
(B) Immunoblot of IP with Flag tag from HEK293T cells transfected with Flag-SKP1, Myc-UBC9, HA-SUMO-1 and gradually increased amount of PIAS3.
(C) Conservation of SUMOylation interacting motif 4A (SIM-4A) in PPM1B amino acid sequence in a variety of organisms.
(D) Immunoblot of Flag-co-immunoprecipitation (Flag-co-IP) from HEK293T cells transfected with SUMOylation-enriching plasmids (HA-SUMO1, Myc-UBC9, SLX4), 2Strep-SKP1 and Flag-tagged PPM1B^{WT} or PPM1B$^{SIM\text{-}4A}$. Control co-IP without transfection of PPM1B plasmids is shown in left lane. WCE controls are shown at the bottom.
Each experiment was performed a minimum of three times with similar results.

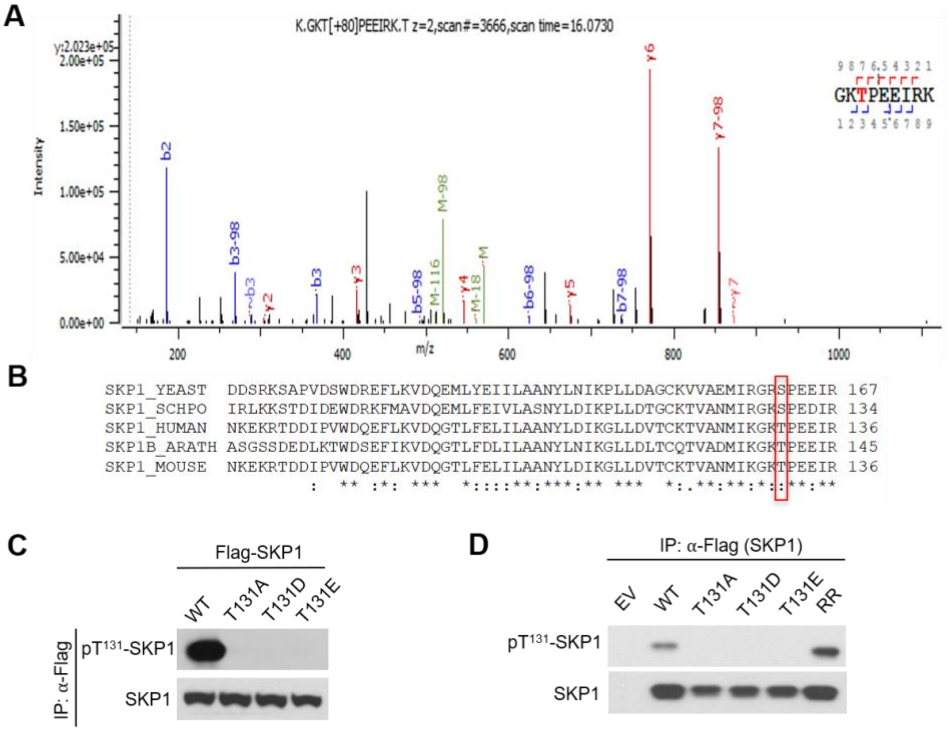

E

	WT		RR	
**Sequence**	**Unmod.**	**Phos.**	**Unmod.**	**Phos.**
TVANMIKGKTPEEIR	0.00E+00	0.00E+00	0.00E+00	4.87E+06
TVANmIKGKTPEEIR	0.00E+00	1.81E+06	0.00E+00	2.43E+07
GKTPEEIR	2.45E+07	1.16E+06	9.38E+07	7.09E+06
GKTPEEIRK	2.69E+07	3.05E+06	1.23E+08	1.59E+07
TPEEIR	6.14E+06	0.00E+00	1.44E+07	0.00E+00
TPEEIRK	1.13E+08	0.00E+00	2.93E+08	0.00E+00
SUM	1.70E+08	6.02E+06	5.24E+08	5.22E+07
%	97	3	91	9

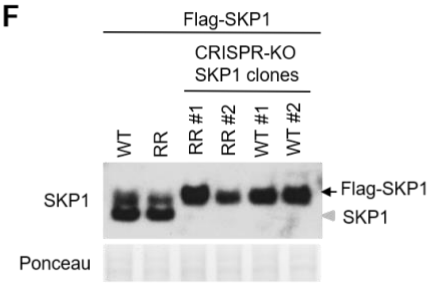

Figure S2. SKP1 SUMOylation promotes PPM1B binding to reduce SKP1 phosphorylation.
(**A**) Liquid chromatography mass spectrometry analysis of immunoprecipitated Flag-SKP1^{WT} from HEK293T cell lysate identified SKP1 phosphorylation at Threonine 131 (T131).
(**B**) Conservation of SKP1 phosphorylation site (red box) across species, including yeast, *Schizosaccharomyces pombe* (schpo), human, *Arabidopsis thaliana* (arath), and mouse.
(**C**) Immunoblot for the indicated proteins after co-immunoprecipitation (co-IP) in HEK293T cell lysate with either Flag-tagged SKP1^{WT}, SKP1^{T131A}, SKP1^{T131D}, or SKP1^{T131E}.
(**D**) Immunoblot for the indicated proteins after co-IP from lysates of HeLa cells stably expressing different Flag-tagged forms of SKP1, including SKP1^{WT}, SKP1^{T131A}, SKP1^{T131D}, SKP1^{T131E}, or SKP1^{RR}.
(**E**) Table containing SKP1 peptides detected via mass spectroscopy from SKP1^{WT} or SKP1^{RR} expressing cells, showing the areas under the curve for all of the peptides that cover T131 with unmodified (Unmod.) or phosphorylated (Phos.) residues in both cell types.
(**F**) Immunoblot for SKP1 in HeLa cells after CRISPR knockout (KO) of endogenous SKP1 and replacement with SKP1^{WT} or SKP1^{RR}. Cells expressing endogenous SKP1 and Flag-tagged SKP1^{WT} or SKP1^{RR} are shown on the left. Black arrow: Flag-SKP1.
Each experiment was performed a minimum of three times with similar results.

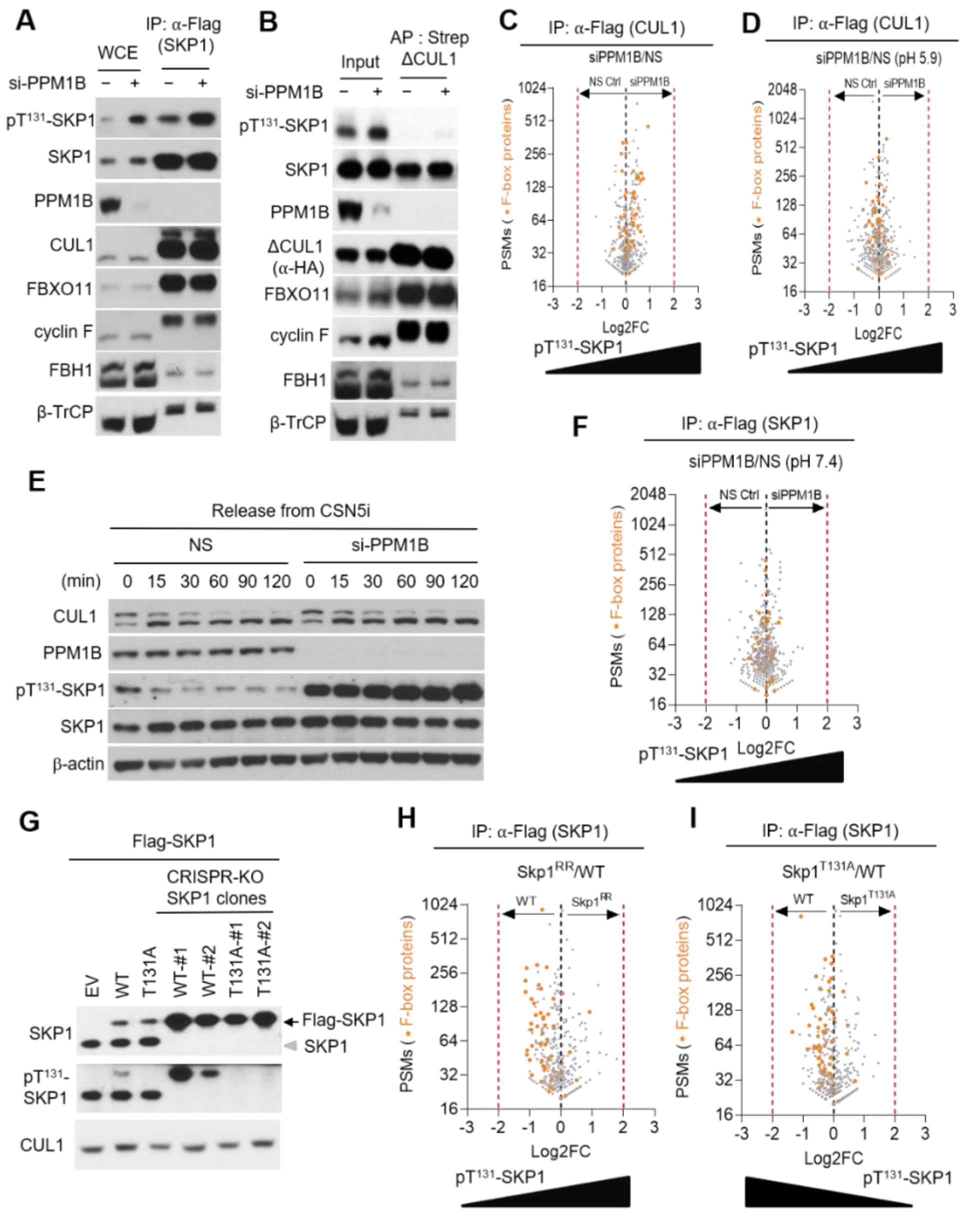

Figure S3. SKP1 phosphorylation does not affect SCF complex assembly.

(**A**) Immunoblot of Flag co-immunoprecipitation (co-IP) in HeLa cells expressing Flag-tagged SKP1 and treated with a control siRNA (-) or siRNA to PPM1B (si-PPM1B) for 48h. Inputs are shown on left.

(**B**) Immunoblot of biotin co-IP in HEK293T cells transfected with 2Strep-HA-ΔCUL1 (1-252 aa) and treated with or without si-PPM1B for 24h. Inputs are shown on left.

(**C-D**) Proteomic analysis of proteins pulled down for Flag-tagged CUL1 in HEK293T cells treated without (-) or with (+) si-PPM1B at pH 7.4 (**C**) or 5.9 (**D**). Values expressed as $\log_2$ fold change (Log_2FC) in interaction with Flag-CUL1 between indicated treatment conditions. Orange dots: F-box proteins.

(**E**) Immunoblot of HeLa cells treated with si-PPM1B or a control (NS) and 16µM CSN5i for 4hr followed by culture in fresh media for the indicated times.

(**F**) Proteomic analysis of proteins pulled down with Flag-SKP1^{WT} at pH of 7.4 (after treatment with or without si-PPM1B. Values expressed as $\log_2$ fold change (Log_2FC) in interaction with Flag-SKP1^{WT} between treatment conditions. Orange dots: F-box proteins.
(**G**) Creation of SKP1^{WT} or SKP1^{T131A} clonal cell lines. Immunoblot for the indicated proteins in HeLa cells after CRISPR knockout (KO) of endogenous SKP1 and replacement with Flag-tagged SKP1^{WT} or SKP1^{T131A}. Cells transfected with an empty vector (EV) or Flag-tagged versions of SKP1^{WT} or SKP1^{T131A} in addition to endogenous SKP1 are shown on the left. Arrows: Flag-SKP1 (black), endogenous SKP1 (grey arrowhead), and SUMO-SKP1 (black arrowhead).
(**H-I**) Proteomic analysis of proteins pulled down with Flag-SKP1 in the indicated conditions: SKP1^{RR} to SKP1^{WT} cells (**H**) and SKP1^{T131A} to SKP1^{WT} cells (**I**). Data is shown as $\log_2$ fold change (Log_2FC) in the interaction of the proteins with Flag-SKP1 against peptide spectra matched (PSMs) for each protein as a proxy for protein coverage. Orange dots: F-box proteins.
Each experiment was performed a minimum of three times with similar results.

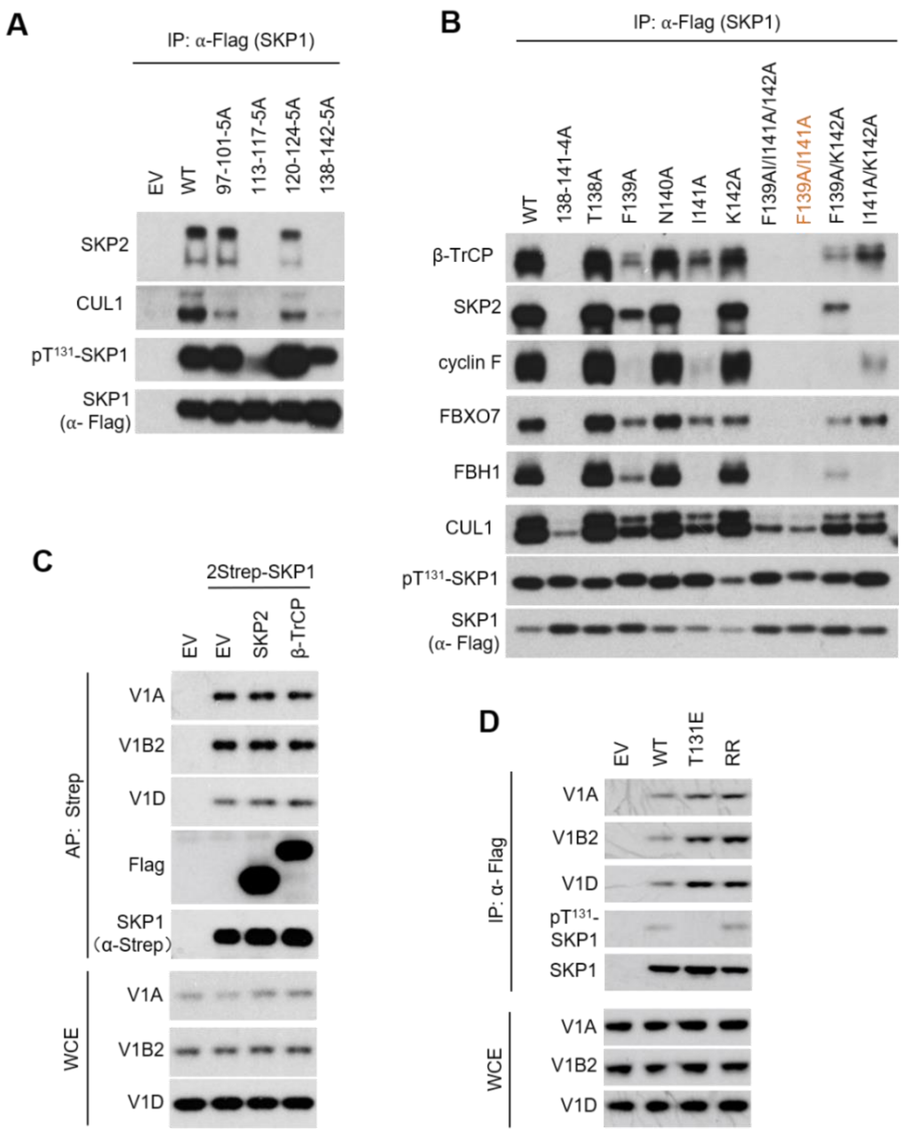

Figure S4. Mapping SKP1 mutant that does not bind F-box proteins but is still phosphorylated on Thr131.

(**A**) Immunoblot of Flag-co-immunoprecipitation (co-IP) in cells expressing Flag-tagged versions of SKP1 containing the indicated five amino acid substitution with alanine, $SKP1^{WT}$ or empty vector (EV).

(**B**) Immunoblot of Flag-co-IP in cells expressing SKP1 proteins with the indicated single or combined mutations or $SKP1^{WT}$.

(**C**) Immunoblot of affinity precipitation with Strep tag from HEK293T cells transfected with 2Strep-SKP1 and Flag-tagged SKP2 or β-TrCP. Control without transfection of SKP1 plasmids is shown in left lane. WCE controls are shown at the bottom.

(**D**) Immunoblot of IP with Flag tag from HEK293T cells transfected with Flag-tagged $SKP1^{WT}$ or $SKP1^{T131E}$ or $SKP1^{RR}$. Control without transfection of SKP1 plasmids is shown in left lane. WCE controls are shown at the bottom.

Each experiment was performed a minimum of three times with similar results.

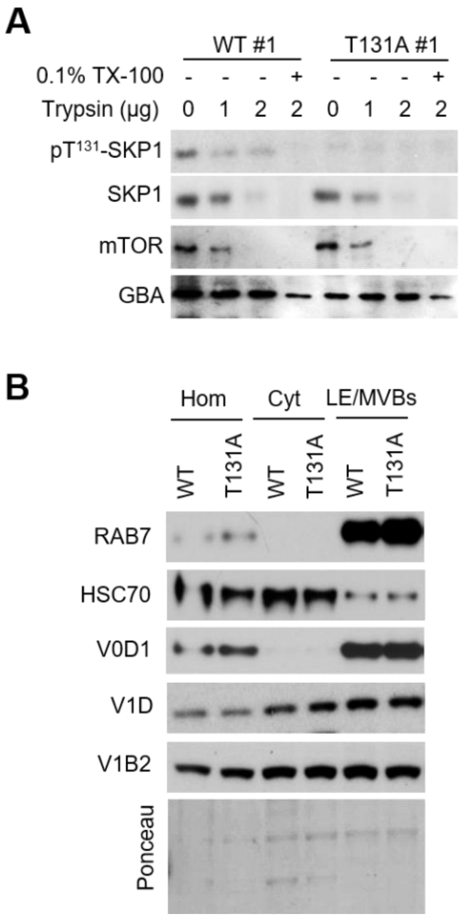

Figure S5. Characterization of SKP1 phosphorylation in the LE/MVB compartment.

(**A**) Topology of pT^{131}-SKP1 in isolated LE/MVBs. Immunoblot of LE/MVBs incubated with increasing concentrations of trypsin and, where indicated, Triton X-100 (TX-100) to allow trypsin access to lumen proteins. GBA and mTOR are shown as example of luminal and membrane-associated proteins, respectively.

(**B**) Immunoblot of subcellular fractions from HeLa cells expressing SKP1^{WT} and SKP1^{T131A} homogenate (H), cytosol (C) and LE/MVBs.

Each experiment was performed a minimum of three times with similar results.

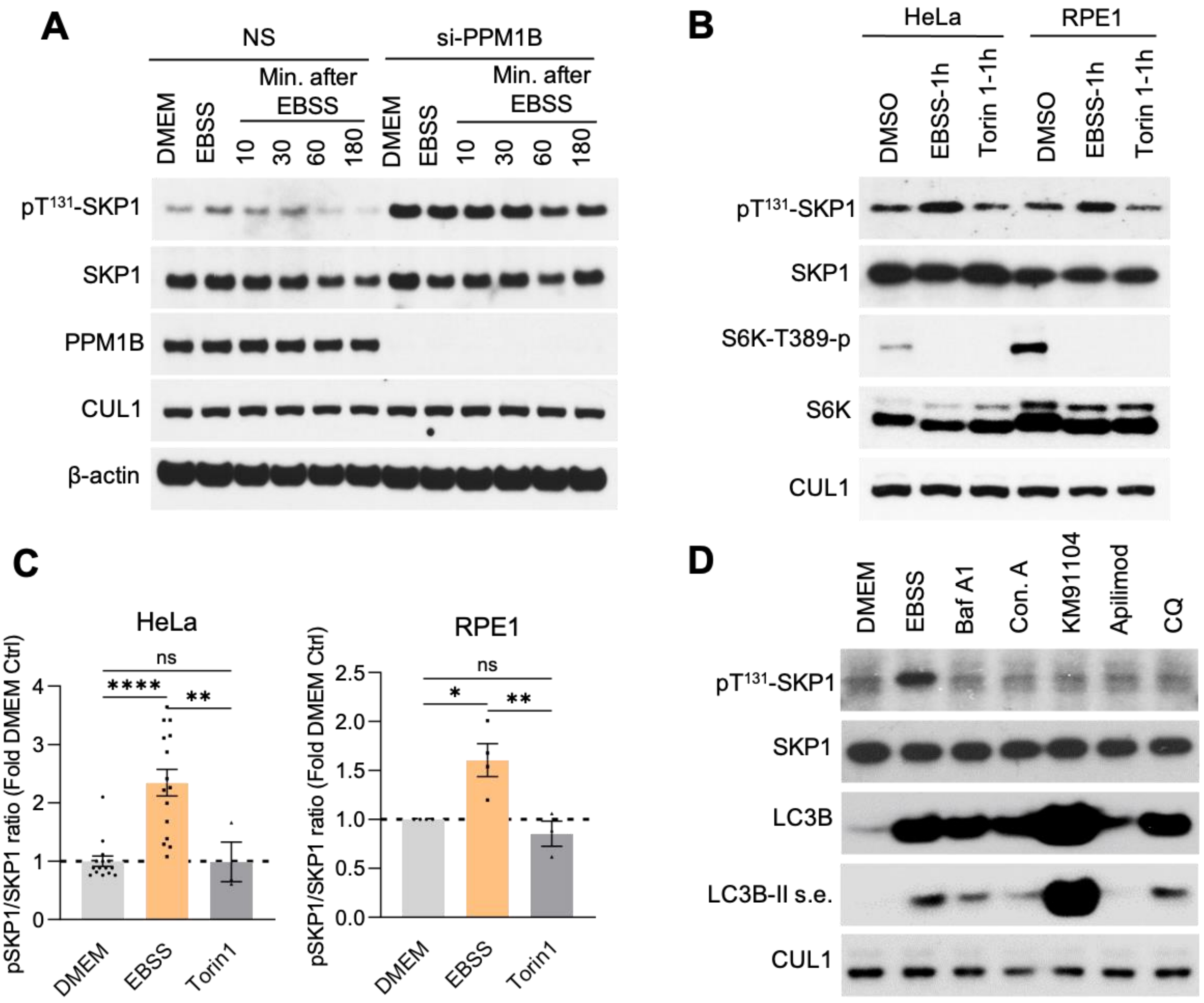

Figure S6. SKP1 phosphorylation is induced by starvation.

(**A**) Immunoblot of RPE1 cells treated with a control siRNA (NS) or siRNA for PPM1B (si-PPM1B) after incubation in full DMEM media, or at the indicated times after 1hr incubation in EBSS followed by DMEM replacement.

(**B-C**) Immunoblot for the indicated proteins (**B**) and quantification of the ratio of p^{T131}-SKP1 to total SKP1 (**C**) of cancerous (HeLa) and non-cancerous (RPE1) cell lines treated with EBSS or the mTORC1 inhibitor Torin1 for 1hr. n=3-15 independent experiments. Reduced phosphorylation of S6K (S6K-T389-p) is used as a positive control for Torin1 inhibitory efficacy.

(**D**) Immunoblot of HeLa cells incubated in complete DMEM media or 1hr incubation in EBSS or in DMEM with 10 nM Baf A1, 100 nM Concanamycin (Con.A), or 24hr incubation in 0.5 μM KM91104 or 0.5 μM Apilimod or 40 μM Chloroquine (CQ). Changes in LC3B-II levels are used as a control for the different lysosomal inhibitors.

Data are mean±s.e.m. and individual values. One-way ANOVA with Bonferroni's multiple comparison's post-hoc test was performed for (C). *p<0.05, **p<0.01, ****p<0.0001. ns: not significant.

Each experiment was performed a minimum of three times with similar results.

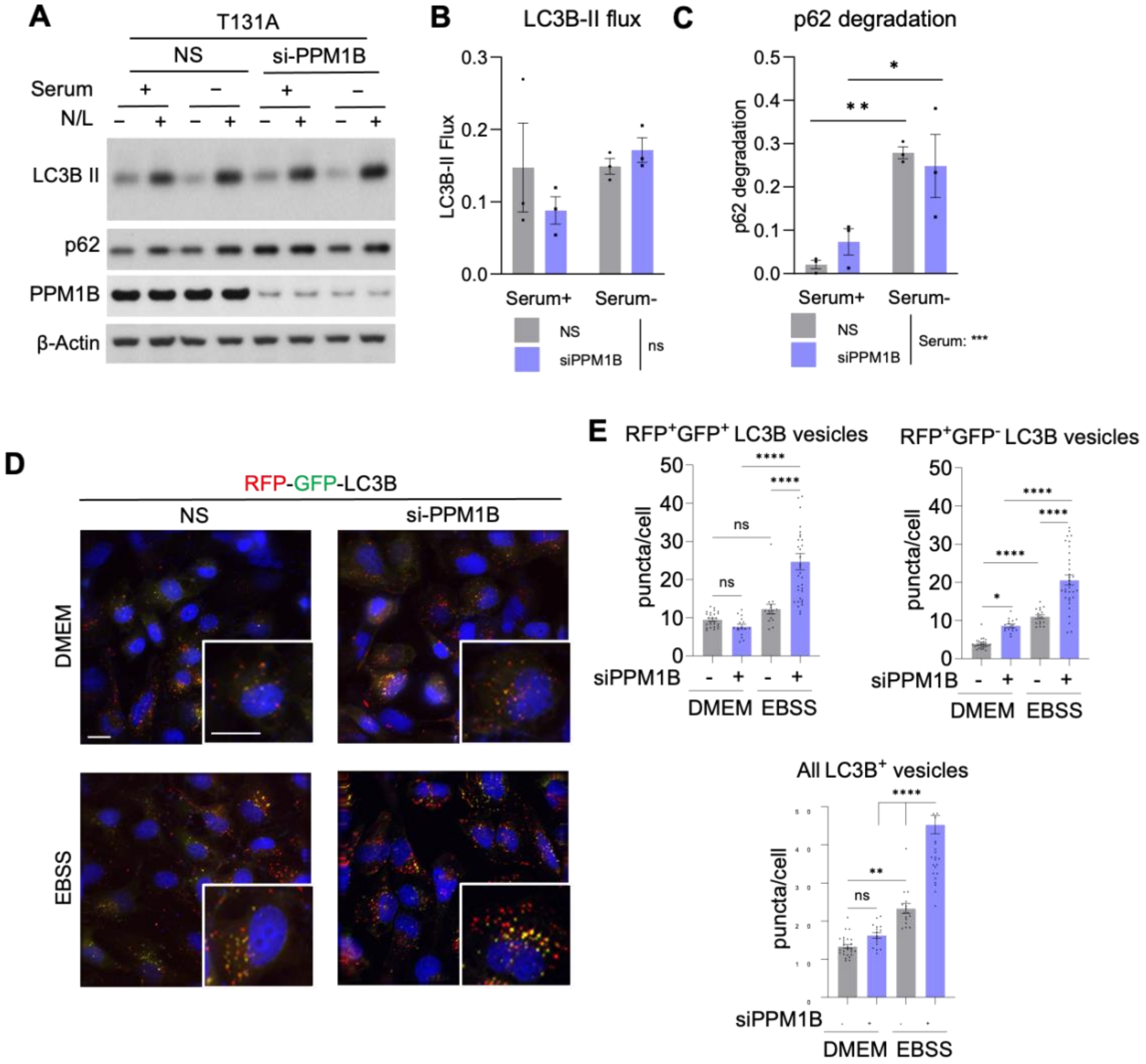

Figure S7. Persistent SKP1 phosphorylation leads to constitutive upregulation of macroautophagy

(A-C) Immunoblot of SKP1^{T131A} HeLa cells treated without (NS) or with siRNA to PPM1B (si-PPM1B) incubated in DMEM supplemented with (+) or without (-) serum for 8 hours in the presence or absence of Ammonium chloride and leupeptin (N/L) to inhibit endolysosomal degradation. Quantification of LC3B-II flux (B) and p62 degradation (C). n=3-6 independent experiments

(**D-E**) Macroautophagy activity in U-2 OS cells expressing the GFP-RFP-LC3B tandem fluorescent reporter treated with si-PPM1B or a control siRNA (NS) and incubated in DMEM media or EBSS for 1hr. Representative images (**D**) and quantification (**E**) of number of neutral pH LC3B^{+} vesicles (GFP^{+}; RFP^{+} puncta) (left), acidic LC3B^{+} vesicles (GFP^{-}; RFP^{+} puncta) (right) and total number of LC3B^{+} vesicles (independently of their pH) (bottom). n>250 cells from 2 cell lines each. Data are mean$\pm$s.e.m. and individual values. Two-way ANOVA with Fisher's LSD post-hoc test (**B,C**) or Bonferroni's post-hoc correction test (**E**) was used. Differences were significant for *p<0.05, **p<0.01, ***p<0.001, ****p<0.0001. ns: not significant.

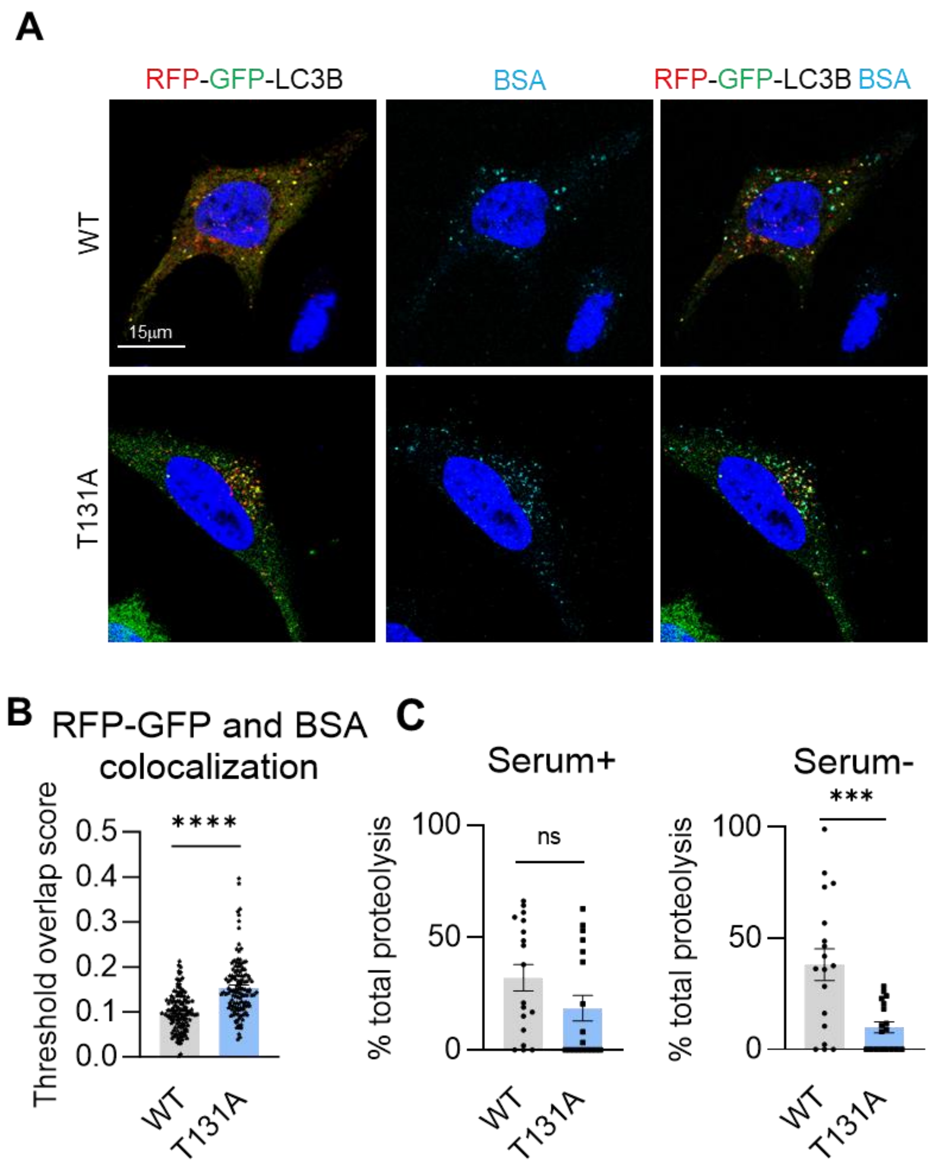

Figure S8. Impact of SKP1 phosphorylation on amphisomes

(**A-B**) Cells expressing SKP1^{WT} and SKP1^{T131A} transduced with the GFP-RFP-LC3B tandem fluorescent reporter incubated with BSA-Alexa 647 to highlight endosomes. Representative images (**A**) and quantification (**B**) of threshold overlap score. N>130 cells from 3 independent experiments. Nuclei are highlighted with DAPI.

(**C**) Degradation of long half-life proteins in cells expressing SKP1^{WT} or SKP1^{T131A} 12hr after culture in serum supplemented (top) or serum deprived (bottom) media. n=3 independent experiments with 3 individual wells per experiment.

Data are mean$\pm$s.e.m. and individual values. Unpaired t-tests (**B**-**C**) were used. Differences were significant for ***p<0.001, ****p<0.0001. ns: not significant.

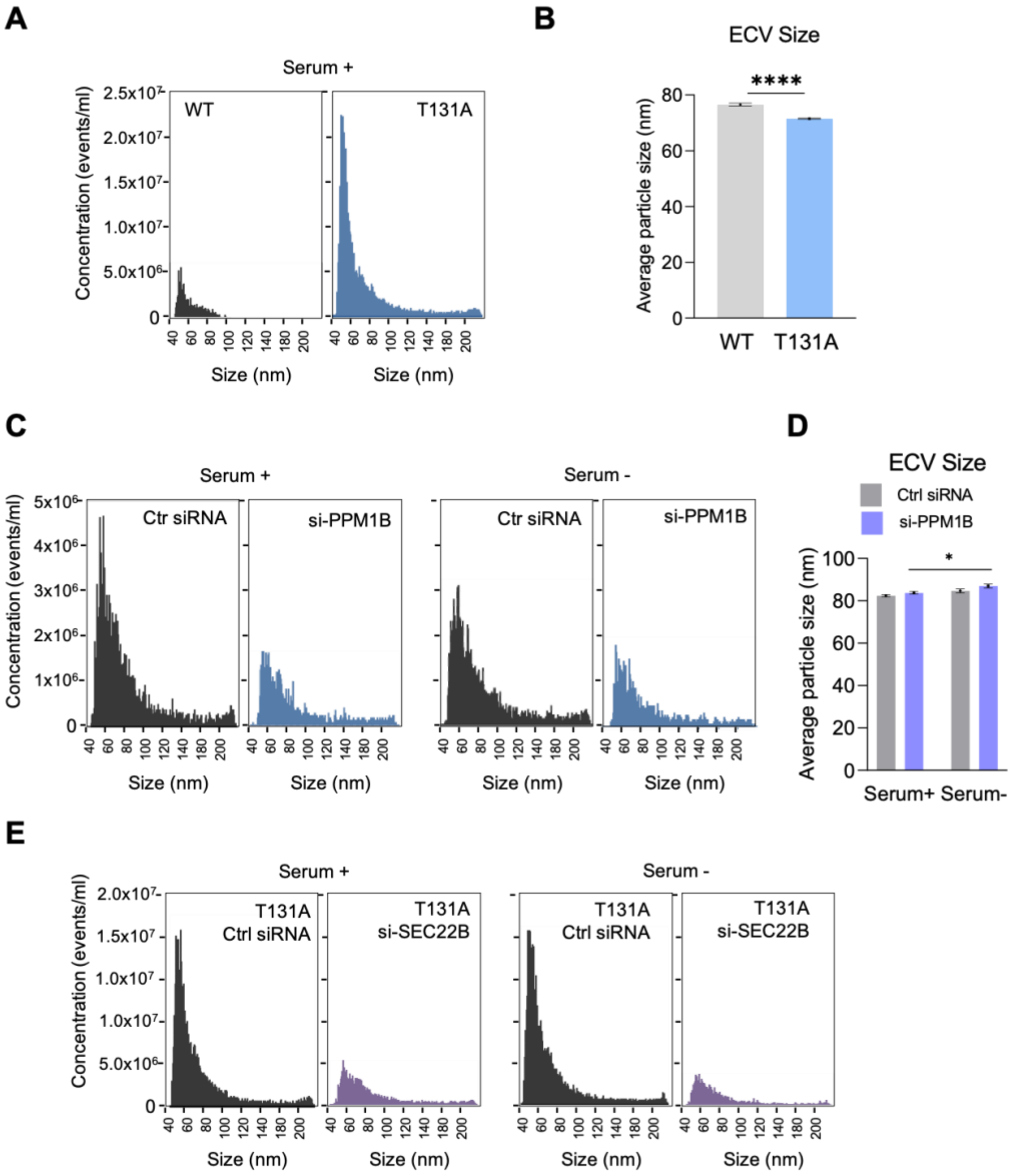

Figure S9: Characterization of extracellular vesicles released in SKP1-dependent manner
(**A-B**) Representative histograms (**A**) and average extracellular vesicle (ECV) size (**B**) based on NanoFCM analysis from ECVs isolated from culture media of $SKP1^{WT}$ and $SKP1^{T131A}$ cells. n=3 independent experiments (ie).
(**C-D**) Representative histograms (**C**) and average extracellular vesicle (ECV) size (**D**) based on NanoFCM analysis from ECVs isolated from culture media of $SKP1^{WT}$ cells treated with control siRNA (Ctrl) or siRNA to PPM1B (si-PPM1B) cultured in DMEM supplemented (+) or not (-) with serum for 16hr. n=3 ie with >4,000 vesicles per condition.
(**E**) Representative histograms of extracellular vesicles (ECVs) size based on NanoFCM analysis from ECVs isolated from culture media of $SKP1^{T131A}$ cells treated with control siRNA (Ctrl) or siRNA to SEC22B (si-SEC22B) cultured in DMEM supplemented (+) or not (-) with serum for 16hr. n=3 ie.
Data are mean$\pm$s.e.m. and individual values. Two-way ANOVA with Bonferroni's multiple comparisons post hoc test (**B-D**) were used. Differences were significant for *p<0.05, ****p<0.0001.

A

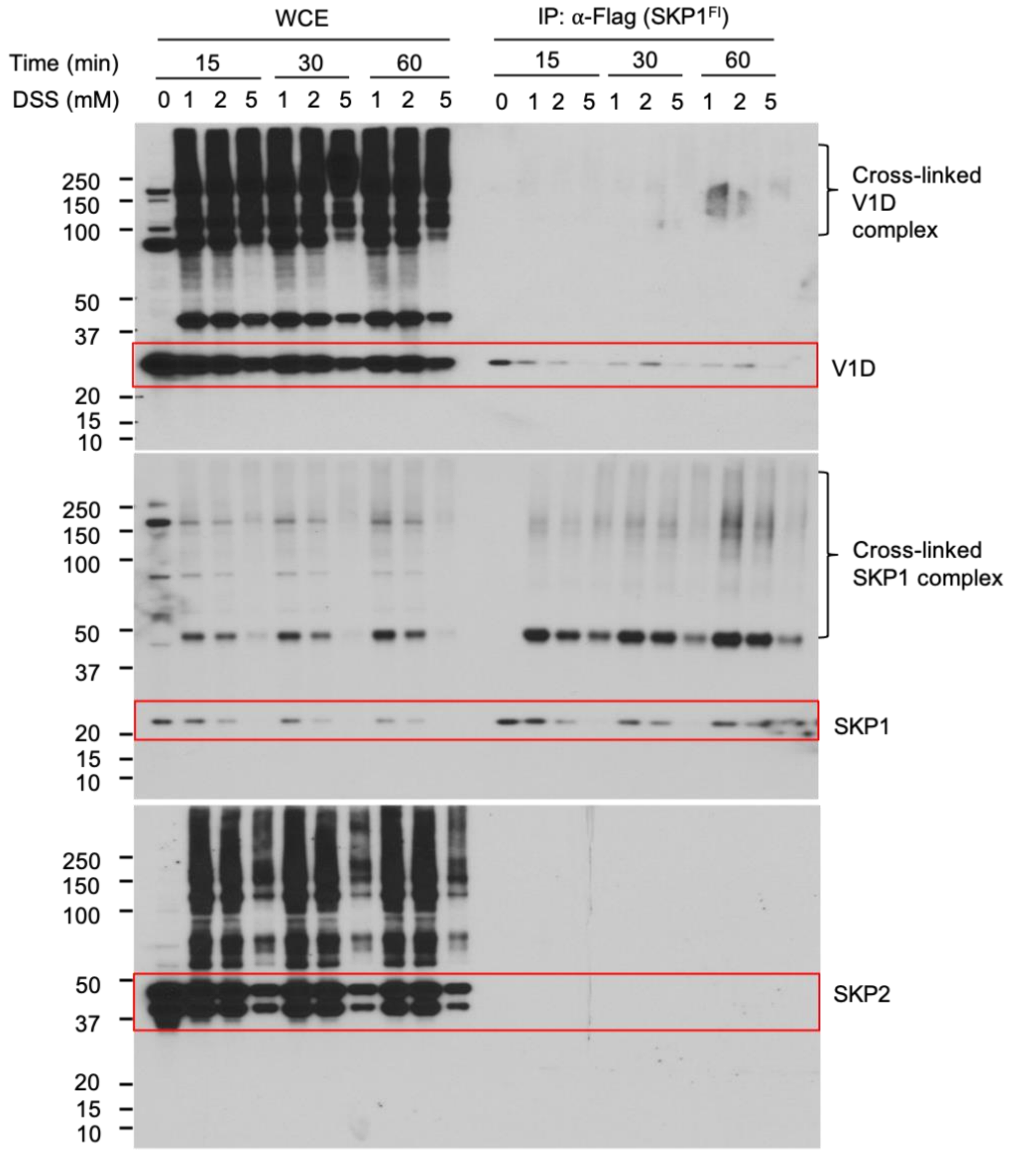

B

SKP1-Fl Crosslink coIP

Peptide	protein_1	position_1	protein_2	position_2	count
DLQQYQSQAKQLFR(10)-GKTPEEIRK(2)	sp|O75396|SEC22B	38	sp|P63208|SKP1	130	8
DLQQYQSQAKQLFR(10)-GKTPEEIR(2)	sp|O75396|SEC22B	38	sp|P63208|SKP1	130	5

Figure S10. SKP1 interacting peptides after co-immunoprecipitation with crosslinking.

(**A**) Full, uncropped immunoblots for V_1D (top), SKP1 (middle) or SKP2 (bottom) after Flag-co-immunoprecipitation in Expi293F cells expressing Flag-SKP1$^{F139A/I141A}$ (SKP1^{FI}) treated with the indicated concentrations of the cross-linking agent disuccinimidyl suberate (DSS). Brackets indicate crosslinked complexes for each protein; red boxes indicate monomeric proteins.

(**B**) Peptides of SEC22B pulled down with Flag-SKP1$^{F139A/I141A}$ (SKP1-FI) in Expi293F cells after treatment with 1 mM DSS for 1h.

This experiment was performed three times with similar results.

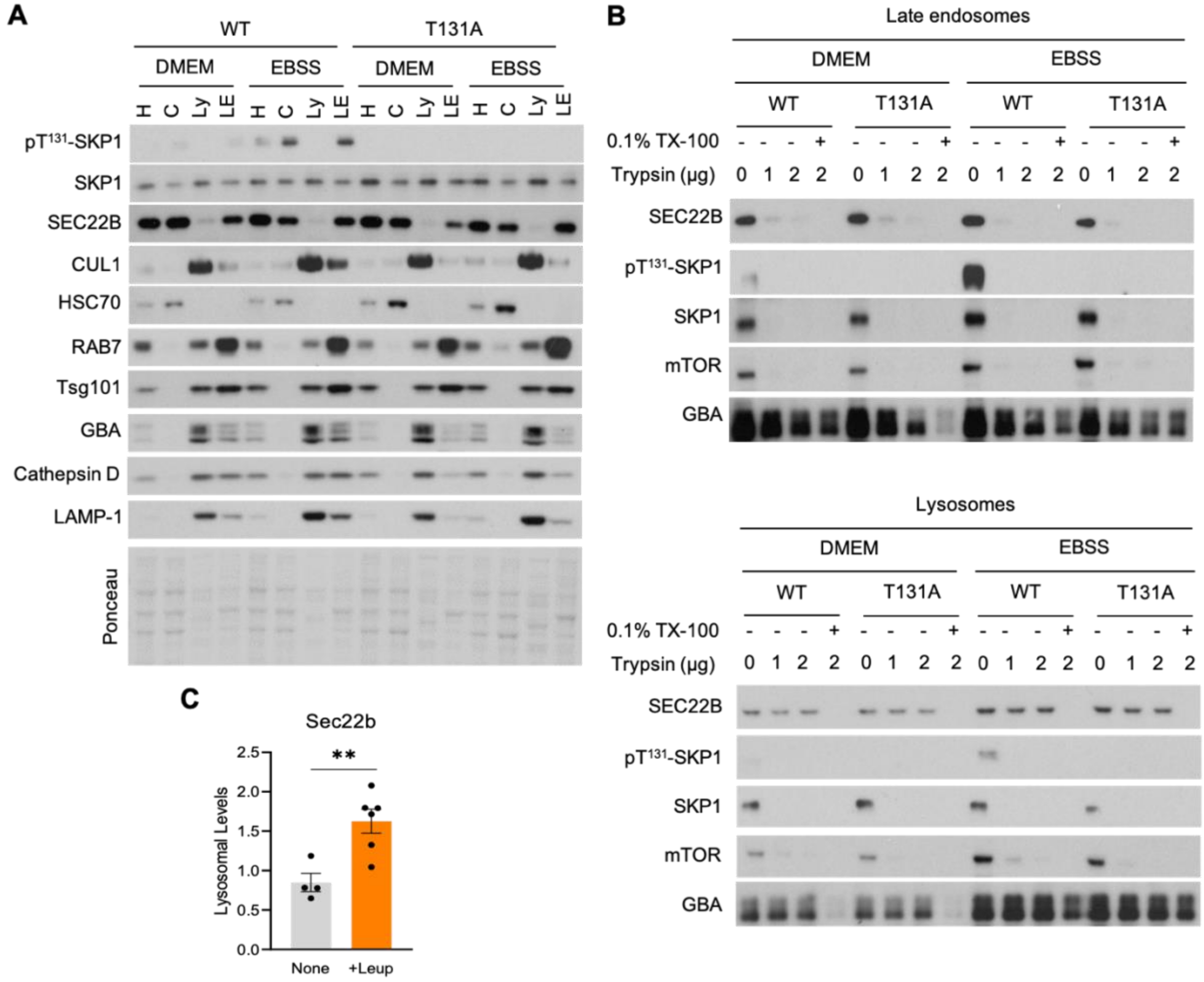

Figure S11. Characterization of SEC22B in late endosome and lysosome fractions.
(**A**) Immunoblot of proteins in homogenate (H), cytosol (C), lysosomes (Ly) or late endosomes/multivesicular bodies (LE) isolated from cells expressing SKP1^{WT} or SKP1^{T131A} after incubation in DMEM or in EBSS for 1hr.
(**B**) Topology of pT^{131}-SKP1 in isolated LE/MVBs (**left**) and lysosomes (**right**). Organelles were incubated with increasing concentrations of trypsin with or without the detergent Triton X-100 (TX-100) to lyse the organelle limiting membrane and expose contents to trypsin. GBA and mTOR are shown as example of luminal and membrane-associated proteins, respectively.
(**C**) Levels of Sec22b in lysosomes isolated from the livers of mice injected without (None) or with the endolysosomal protease inhibitor leupeptin (+Leup). N=4-6 mice. Data are mean$\pm$s.e.m. and individual values. Unpaired t-test (**C**) was used. Difference was significant for **$p<0.01$.
Each experiment was performed a minimum of three times with similar results.

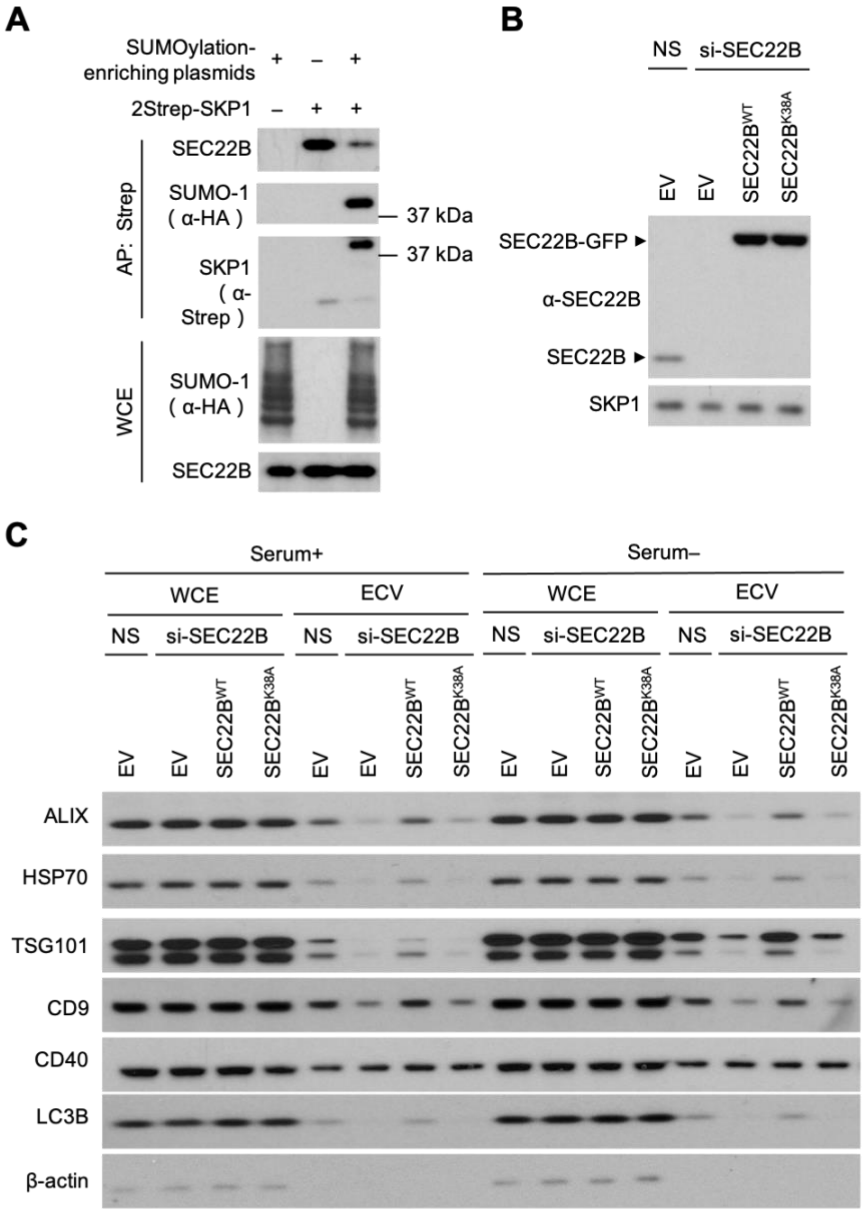

Figure S12: The interaction between SKP1 and SEC22B is essential for the contribution of SEC22B to extracellular secretion.
(**A**) Immunoblot for indicated proteins from affinity purification with streptavidin-tagged SKP1 in cells treated without (-) or with (+) SUMOylation enriching conditions. Whole cell extract (WCE) is shown below as a control.
(**B**) Immunoblot for indicated proteins of HeLa cells treated with a control siRNA (NS) or siRNA to SEC22B (si-SEC22B) and subsequent introduction of an empty vector (EV), WT SEC22B (SEC22B^{WT}), and a form of SEC22B that cannot bind SKP1 (SEC22B^{K38A}).
(**C**) Immunoblot for indicated proteins of whole cell extract (WCE) and isolated extracellular vesicles (ECV) from cells treated without or with si-SEC22B and expressing a control empty vector (EV), SEC22B^{WT}, or SEC22B^{K38A} construct in nutrient rich (Serum+) or nutrient poor (Serum-) conditions.
Each experiment was performed a minimum of three times with similar results.

Other Supplementary Materials included in this paper are listed below and provided as Excel files:

- **Supplementary Table 1.** Search results of MS analysis for proteins co-IPed from HEK293T cells transiently expressing $SKP1^{WT}$ or $SKP1^{RR}$ with SUMO-enriching plasmids
- **Supplementary Table 2.** Search results of MS analysis for proteins co-IPed from HEK293T cells transiently expressing CUL1 with or without PPM1B silencing
- **Supplementary Table 3.** Search results of MS analysis for proteins co-IPed from HeLa cells stably expressing $SKP1^{WT}$ or $SKP1^{RR}$
- **Supplementary Table 4.** Search results of MS analysis for proteins co-IPed from HeLa cells stably expressing $SKP1^{WT}$ or $SKP1^{T131A}$
- **Supplementary Table 5.** Search results of MS analysis for proteins co-IPed at pH 7.4 from HEK293T cells transiently expressing $SKP1^{WT}$, $SKP1^{RR}$, $SKP1^{T131A}$ or $SKP1^{FI}$
- **Supplementary Table 6.** Search results of MS analysis for proteins co-IPed at pH 5.9 from HEK293T cells transiently expressing $SKP1^{WT}$, $SKP1^{T131A}$ or $SKP1^{FI}$
- **Supplementary Table 7** Search results of MS analysis for ECVs isolated from the culture media of $SKP1^{WT}$ and $SKP1^{T131A}$ cells
- **Supplementary Table 8.** Search results of cross-linking MS analysis for proteins co-IPed from Expi293F cells transiently expressing $SKP1^{FI}$
- **Key Table for mass spectrometry raw files-1**
- **Key Table for mass spectrometry raw files-2**
- **Raw data table**

Supplementary table 9. Key Reagents Table

REAGENT or RESOURCE	SOURCE	IDENTIFIER
Antibodies		
rabbit anti-SKP1 (IB: 1:5000)	Michele Pagano's lab	
rabbit anti-pT^{131}-SKP1 (IB: 1:1000)	Michele Pagano's lab	
mouse anti-SUMO1 (IP: 10 ug/ml)	Developmental Studies Hybridoma Bank	Cat # SUMO-1 21C7 RRID:AB_87007
rabbit anti-UBC9 (IB: 1:1000)	Cell Signaling Technology	Cat # 4918S RRID:AB_2210600
rabbit anti-Flag (IB: 1:1000)	Sigma-Aldrich	Cat # F7425-.2MG RRID:AB_439687
rabbit anti-PPM1B (IB: 1:1000)	Bethyl Laboratories	Cat # A300-887A RRID:AB_2170086
rabbit anti-CUL1 (IB: 1:1000)	Thermo Fisher Scientific	Cat # 71-8700 RRID:AB_87955
Mouse anti-HA (IB: 1:1000)	BioLegend	Cat # 901503 RRID:AB_2565005
Mouse anti-Myc (9E10) (IB: 1:1000)	Santa Cruz Biotechnology	Cat # sc-40 RRID:AB_627268
Rabbit anti-FBXO11 (IB: 1:1000)	Novus Biologicals	Cat # NB100-59826 RRID:AB_892469
rabbit anti-cyclin F (IB: 1:1000)	Santa Cruz Biotechnology	Cat # sc-952 RRID:AB_2071212
rabbit anti-FBH1(IB: 1:1000)	Michele Pagano's lab (PMID: 23319600)	
rabbit anti-β-TRCP (IB: 1:1000)	Cell Signaling Technology	Cat # 4394S RRID:AB_10545763
Mouse anti-V1A (IB: 1:1000)	Abnova	Cat # H00000523-M02 RRID:AB_1672274
mouse anti-V1B2 (IB: 1:1000)	Santa Cruz Biotechnology	Cat # sc-166045 RRID:AB_2062496
rabbit anti-V1D (IB: 1:1000)	Novus Biologicals	Cat # NBP1-88895 RRID:AB_11020241
rabbit anti-SKP2 (D3G5) (IB: 1:1000)	Cell Signaling Technology	Cat # 2652S RRID:AB_11178941
rabbit anti-Rab7 (D95F2) (IB: 1:5000)	Cell Signaling Technology	Cat # 9367; RRID:AB_1904103
Rabbit anti-V0D1(IB: 1:1000)	Proteintech	Cat # 18274-1-AP RRID:AB_2258877
Rabbit anti-FBXO7 (IB: 1:1000)	Proteintech	Cat # 10696-1-AP RRID:AB_2246820
Rabbit anti-Lamin B (IB: 1:1000)	Abcam	Cat # ab16048 RRID:AB_443298

Mouse anti-ACTIN (IB: 1:5000)	Sigma-Aldrich	Cat # A5441<br>RRID:AB_476744
Rabbit anti-LC3B (IB: 1:10,000)	Novus Biologicals	Cat # NB100-2220<br>RRID:AB_578334
Mouse anti-p62 (IB: 1:1000)	Abcam	Cat # ab56416<br>RRID:AB_945626
Rabbit anti-hsp70 (IB: 1:1000)	Enzo	Cat # ADI-SPA-757<br>RRID:AB_10616383
Rabbit anti-Tsg101(IB: 1:1000)	Abcam	Cat # ab30871<br>RRID:AB_2208084
Mouse anti-CD63<br>(Immune-EM: 1:50)	Abcam	Cat # ab59479<br>RRID:AB_940915
Rabbit anti-CD63 (IB: 1:1000)	Proteintech	Cat # 25682-1-AP<br>RRID:AB_2783831
Rabbit anti-Aldolase C (IB: 1:1000)	Proteintech	Cat # 14884-1-AP<br>RRID:AB_2226691
Rabbit anti-Sec22B (IB: 1:1000)	Abcam	Cat # ab181076
Rabbit anti-mTOR (IB: 1:1000)	Cell Signaling	Cat # 2972;<br>RRID:AB_330978
Rabbit anti-GBA (IB: 1:1000)	Sigma	Cat # G4171;<br>RRID:AB_1078958
Rabbit anti-V1B (IB: 1:1000)	Abcam	Cat # ab200839
goat anti-rabbit IgG HRP (IB: 1:5000)	KPL	Cat# 5067202
goat anti-mouse IgG HRP (IB: 1:5000)	KPL	Cat# 5067205
goat anti-mouse IgM HRP<br>(IB: 1:5000)	Jackson Immunoresearch	Cat# 115-035-075;<br>RRID:AB_2338508
Rabbit Anti-Mouse IgG<br>(Immune-EM: 1:50)	Jackson Immunoresearch	Cat# 315-005-003<br>RRID:AB_2340033
Goat anti-Rabbit IgG conjugated with18nm gold (Immune-EM: 1:50)	Jackson Immunoresearch	Cat# 111-215-144<br>RRID:AB_2338017
Chemicals, peptides, and recombinant proteins		
Ammonium Chloride	Sigma-Aldrich	Cat # A9434
Leupeptin	ThermoFisher Scientific	Cat # BP2662-25
Pierce ECL Western Blotting Substrate	Thermo Fisher Scientific	Cat #32106
Percoll	GE Healthcare	Cat# 17-0891-02
LysoSensor Yellow/Blue DND-160	Invitrogen	Cat# L7545
MG132	Peptide international	Cat# IZL-3175v
CSN5i	Novartis	PMID: 27774986
anti-Flag resin	Sigma-Aldrich	Cat# A2220
Biotin resin	IBA	Cat# 2-4090-010
Recombinant Protein G Agarose	ThermoFisher Scientific	Cat# 15920010

Bafilomycin A	Santa Cruz Biotechnology	Cat# sc-201550A
EBSS	Gibco	Cat# 24010043
LysoTracker Red	Invitrogen	Cat# L12492
Polybrene	Sigma-Aldrich	Cat# H9268-10G
Lipofectamine™ 3000	ThermoFisher Scientific	Cat# L3000001
Lipofectamine RNAiMAX	ThermoFisher Scientific	Cat# 13778500
Polyethylenimine (PEI)	Polysciences, Inc	Cat# 24765-1
Disuccinimidyl suberate (DSS)	ThermoFisher Scientific	Cat# 21655

Experimental models: Cell lines		
HeLa	ATCC	CCL-2 RRID:CVCL_0030
U-2 OS	ATCC	HTB-96 RRID:CVCL_0042
HCT116	ATCC	CCL-247 RRID:CVCL_0291
RPE1	ATCC	CRL-4000 RRID:CVCL_4388
HEK293T	ATCC	CRL-3216 RRID:CVCL_0063
Expi293F	Thermo Fisher Scientific	A14527 RRID:CVCL_D615
Recombinant DNA		
pE1E2S1	PMID: 15246018	Gift from Dr. Hisato Saitoha
pE1E2S2	PMID: 15246018	Gift from Dr. Hisato Saitoha
HA-SUMO1	PMID: 9452416	Addgene 17359 RRID:Addgene_17359
Myc-UBC9	PMID: 9353268	Addgene 20082 RRID:Addgene_20082
SLX4	RefSeq: BC065125	transOMIC technologies BC065125-seq
CBX4	PMID: 24434214	Cat # Addgene 82513 RRID:Addgene_82513
PIAS1	PMID: 9724754	Cat # Addgene 15206 RRID:Addgene_15206

PIASy	PMID: 11248056	Cat # Addgene 15208 RRID:Addgene_15208
PIAS2	RefSeq: BC015190	transOMIC technologies BC015190-seq
PIAS3	RefSeq: BC001154	transOMIC technologies BC001154-seq
MMS21	RefSeq: BC032797	transOMIC technologies BC032797-seq
EGR2	RefSeq: BC035625	transOMIC technologies BC035625-seq
Flag-SKP1-K142R	Michele Pagano's lab	
Flag-SKP1-K163R	Michele Pagano's lab	
Flag-SKP1-RR (K142R/K163R)	Michele Pagano's lab	
Flag-SKP1-T131A	Michele Pagano's lab	
Flag-SKP1-T131D	Michele Pagano's lab	
Flag-SKP1-T131E	Michele Pagano's lab	
Flag-SKP1-97-101-5A	Michele Pagano's lab	
Flag-SKP1-113-117-5A	Michele Pagano's lab	
Flag-SKP1-120-124-5A	Michele Pagano's lab	
Flag-SKP1-138-142-5A	Michele Pagano's lab	
Flag-SKP1-138-141-4A	Michele Pagano's lab	
Flag-SKP1-T138A	Michele Pagano's lab	
Flag-SKP1-F139A	Michele Pagano's lab	
Flag-SKP1-N140A	Michele Pagano's lab	
Flag-SKP1-I141A	Michele Pagano's lab	
Flag-SKP1-K142A	Michele Pagano's lab	
Flag-SKP1-F139A/I141A/K142A	Michele Pagano's lab	
Flag-SKP1-F139A/I141A	Michele Pagano's lab	
Flag-SKP1-F139A/K142A	Michele Pagano's lab	
Flag-SKP1-I141A/K142A	Michele Pagano's lab	
pET-24d-Flag-SKP1	Michele Pagano's lab	
pBabe-Flag-SKP1	Michele Pagano's lab	
2strep-HA-ΔCUL1	Michele Pagano's lab	
Flag-SKP2	Michele Pagano's lab	
Flag-β-TrCP	Michele Pagano's lab	
Flag-FBXO5	Michele Pagano's lab	
Flag-FBXO31	Michele Pagano's lab	
Flag-CUL1	Michele Pagano's lab	
Flag-V0A	Dafna Bar-Sagi's lab	

Flag-V0D1	PMID: 22053050	Cat # Addgene 87931 RRID:Addgene_87931
RFP-GFP-LC3	PMID: 19148225	Cat # Addgene 22418 RRID:Addgene_22418
STX17	PMID: 23217709	Cat # Addgene 86777 RRID:Addgene_86777
Oligonucleotides		
sgRNA	DNA sequence	
skp1-sgRNA#1--exon1-T	CACCGACTATTAAGACCATGTTGGA	
skp1-sgRNA#1-exon1-B	AAACTCCAACATGGTCTTAATAGTC	
skp1-sgRNA#2-exon2-T	CACCGGGTTTTCTTAGATTTGGGAA	
skp1-sgRNA#2-exon2-B	AAACTTCCCAAATCTAAGAAAACCC	
siRNA	Source	Catalog #
Non-specific targeting siRNA (CGUACGCGGAAUACUUCGA)	Dharmacon	Cat# D-001810-01-20
SMARTpool: ON-TARGETplus PPM1B siRNA	Dharmacon	Cat# L-008281-01-0050
Software and algorithms		
Fiji	NIH	http://fiji.sc/ RRID:SCR_002285
GraphPad	Prism	http://www.graphpad.com/; RRID:SCR_002798
Adobe Photoshop 6.0	Adobe Systems	www.adobe.com; RRID:SCR_014199
PhosphoSitePlus		RRID:SCR_001837 https://www.phosphosite.org/
BioRender	Biorender	https://biorender.com/ RRID:SCR_018361

Other		
Fujifilm LAS-3000 Imager	Fujifilm, Albert Einstein College of Medicine	http://www.fujifilm.com/products/medical/
Konica SRX-101A Tabletop X-Ray Film Processor	Konica, New York University School of medicine	https://www.zzmedical.com/x-ray-practices/konica-srx101a.html
DeltaVision Elite inverted microscope	Applied Precision	
Tecan Infinite 200 Pro microplate reader	Tecan	
pLink2 (v2.3.9)		https://www.cog-genomics.org/plink/2.0/general_usage#cite
pFind3 (v.3.1.5)		http://pfind.org/software/pFind/index.html#Cite%20us

Uncropped membranes

Uncropped membranes used for Western blots corresponding to all main and supplementary figures. Red boxes mark cropped areas shown in the main and supplementary figure of the membranes blotted for the proteins indicated on the right. In the indicated instances, ponceau staining was used as internal loading control. Asterisks denote non specific or relevant bands. Molecular weight markers are color coded according to the key shown on the bottom right.

1A

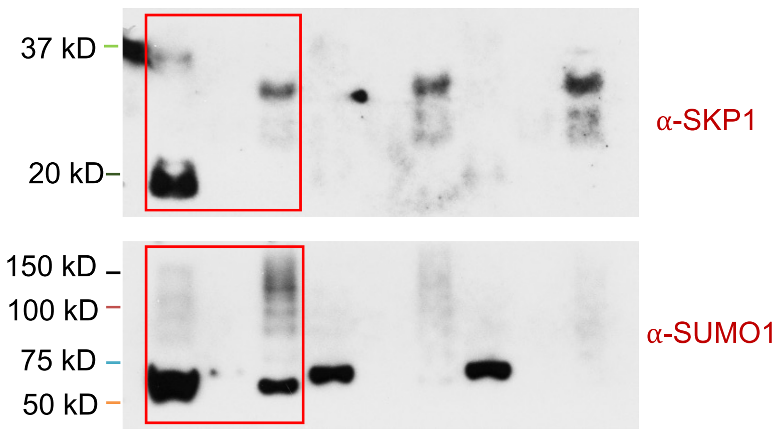
37 kD
20 kD
α-SKP1
150 kD
100 kD
75 kD
50 kD
α-SUMO1

1B

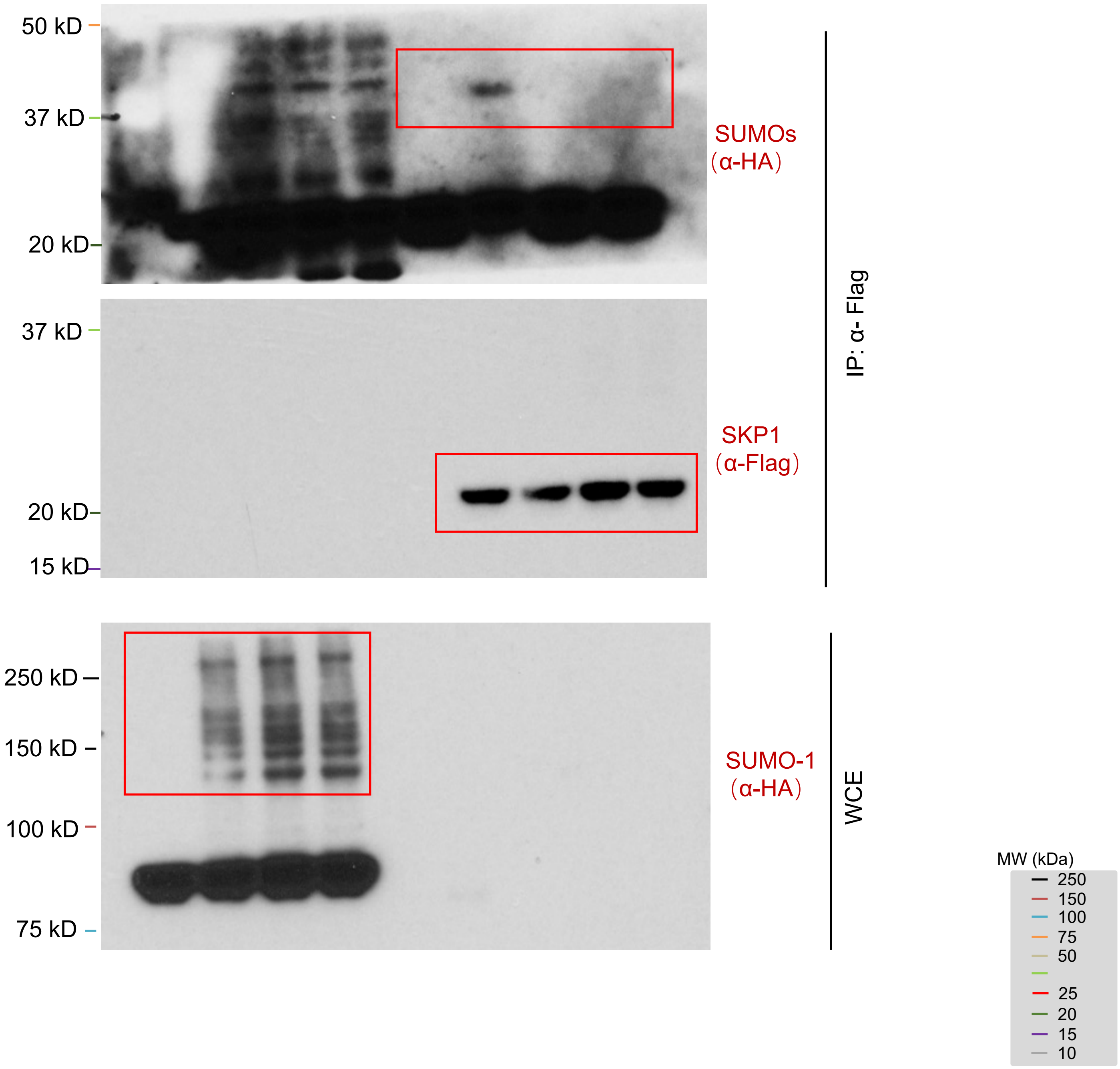
50 kD
37 kD
20 kD
SUMOs
(α-HA)
37 kD
20 kD
15 kD
SKP1
(α-Flag)
IP: α- Flag
250 kD
150 kD
100 kD
75 kD
SUMO-1
(α-HA)
WCE
MW (kDa)
250
150
100
75
50
25
20
15
10

1C

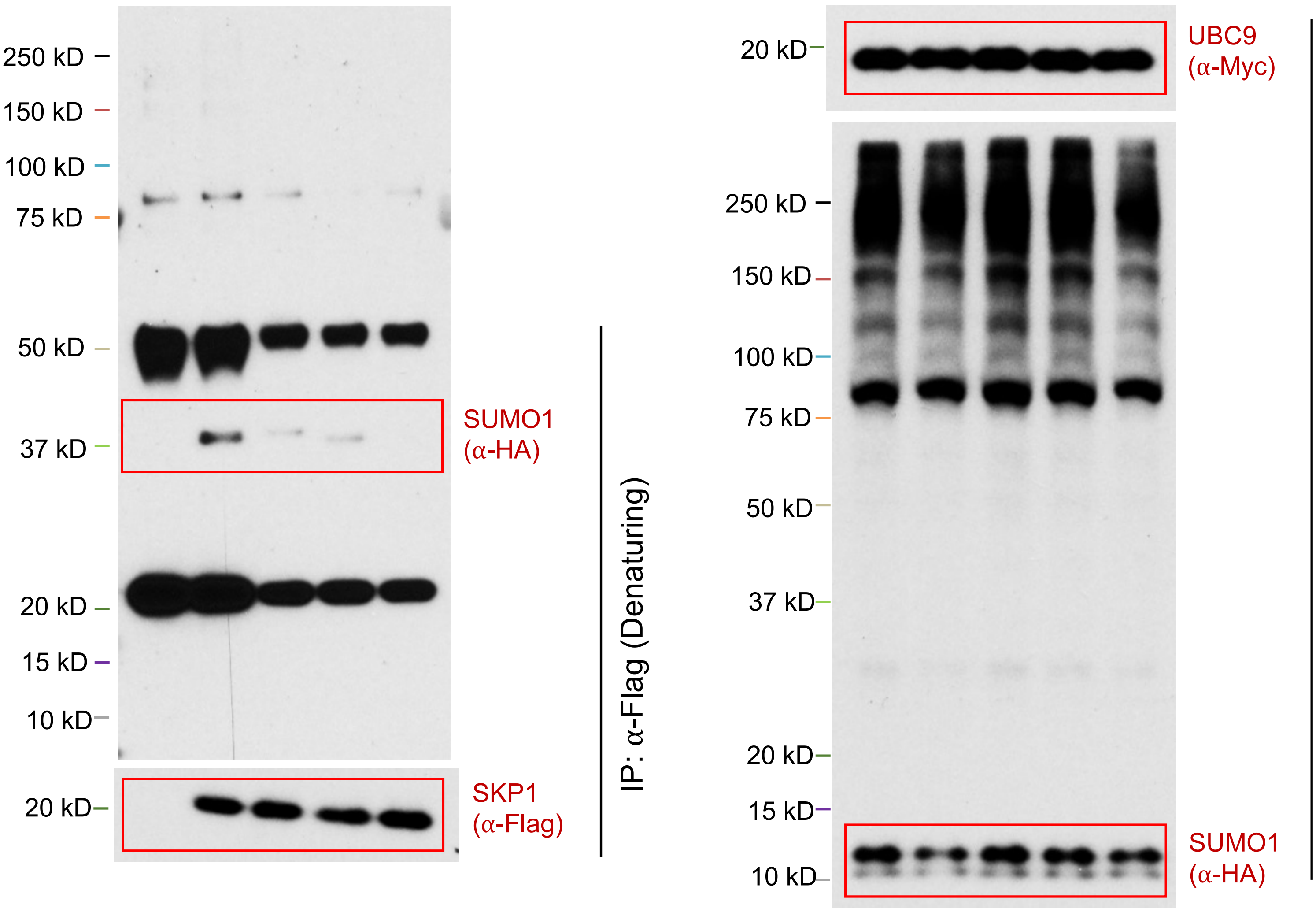
250 kD
150 kD
100 kD
75 kD
50 kD
37 kD
20 kD
15 kD
10 kD
SUMO1
(α-HA)
20 kD
SKP1
(α-Flag)
IP: α-Flag (Denaturing)
20 kD
UBC9
(α-Myc)
250 kD
150 kD
100 kD
75 kD
50 kD
37 kD
20 kD
15 kD
10 kD
SUMO1
(α-HA)

1D

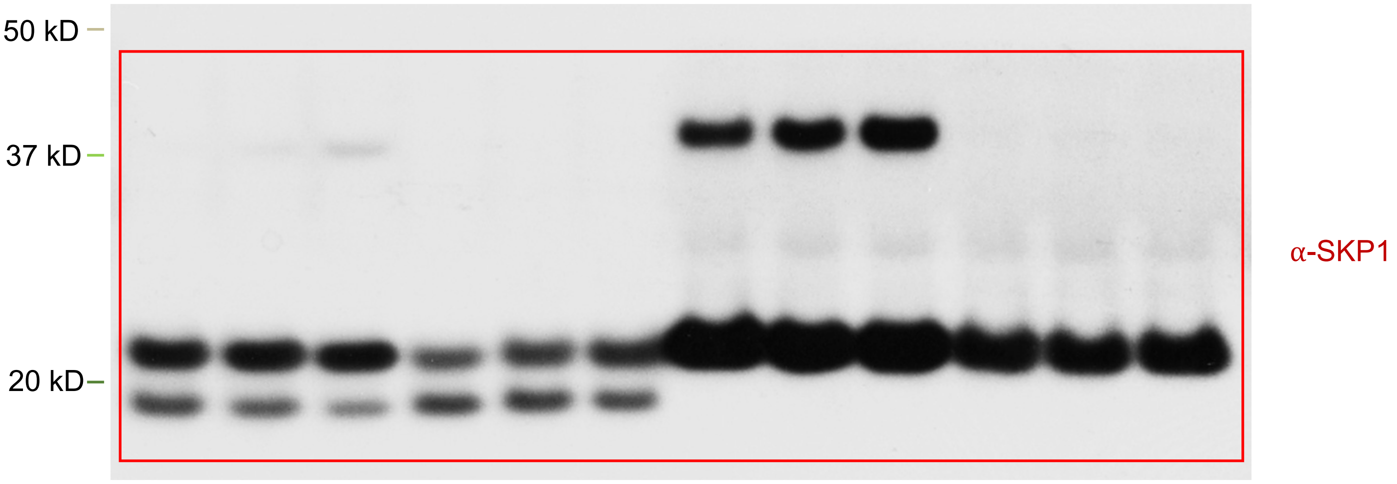
50 kD
37 kD
20 kD
α-SKP1

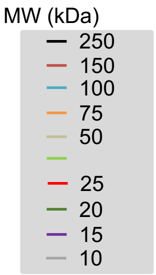
MW (kDa)
250
150
100
75
50
25
20
15
10

1F

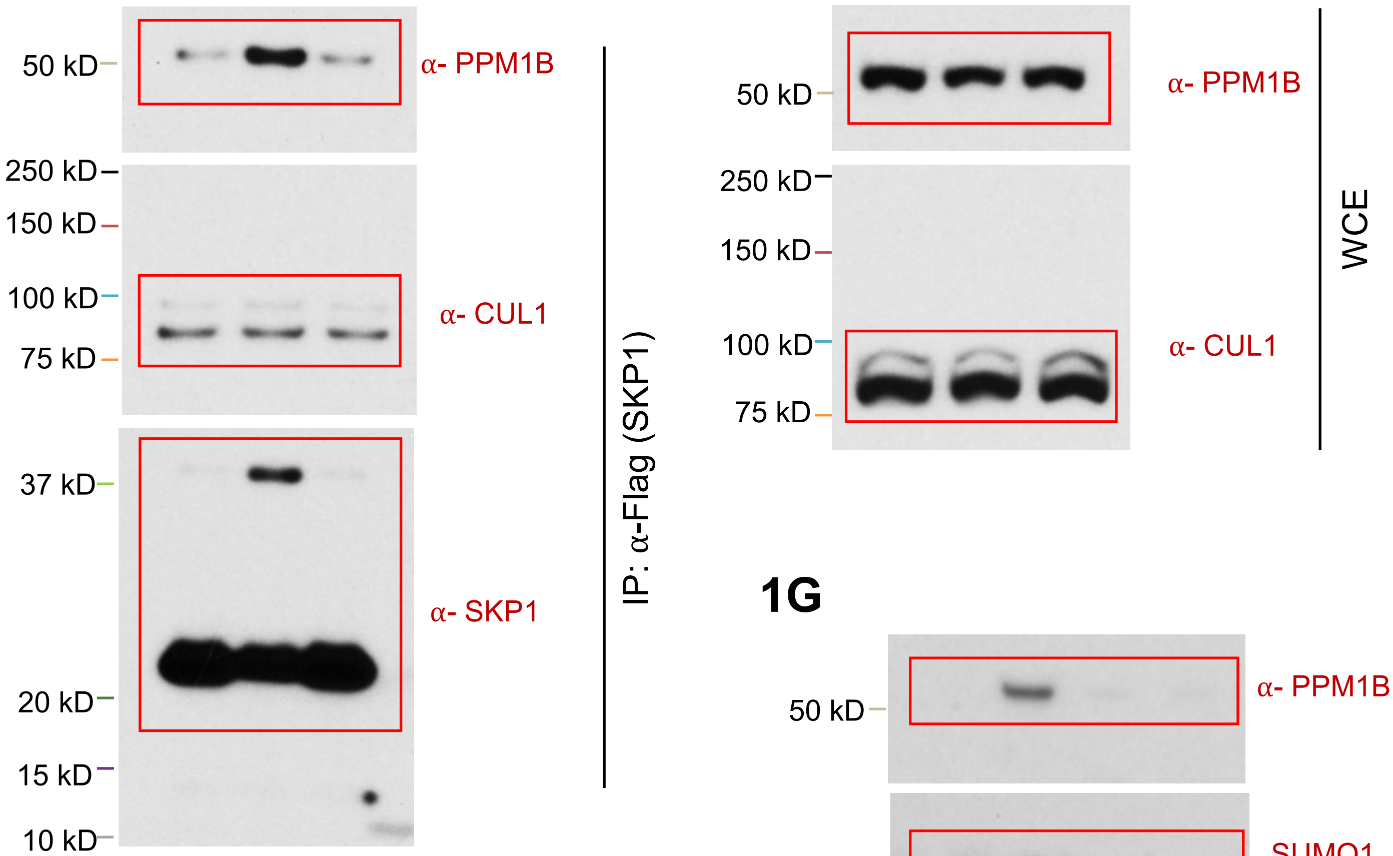

50 kD
α- PPM1B
250 kD
150 kD
100 kD
75 kD
α- CUL1
37 kD
α- SKP1
20 kD
15 kD
10 kD
IP: α-Flag (SKP1)
50 kD
α- PPM1B
250 kD
150 kD
100 kD
75 kD
α- CUL1
WCE

1G

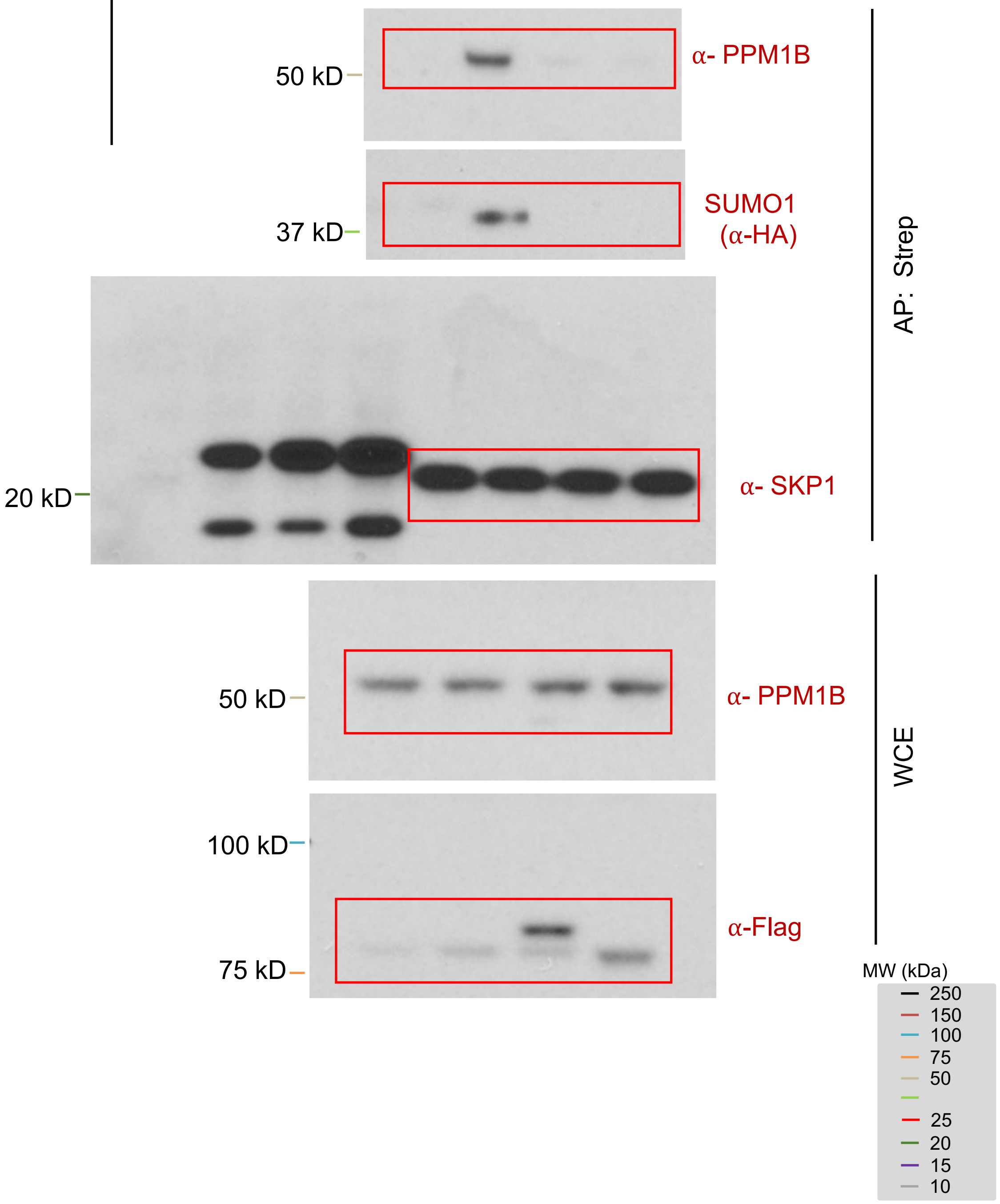

50 kD
α- PPM1B
37 kD
SUMO1
(α-HA)
20 kD
α- SKP1
AP: Strep
50 kD
α- PPM1B
100 kD
75 kD
α-Flag
WCE
MW (kDa)
250
150
100
75
50
25
20
15
10

1H-1

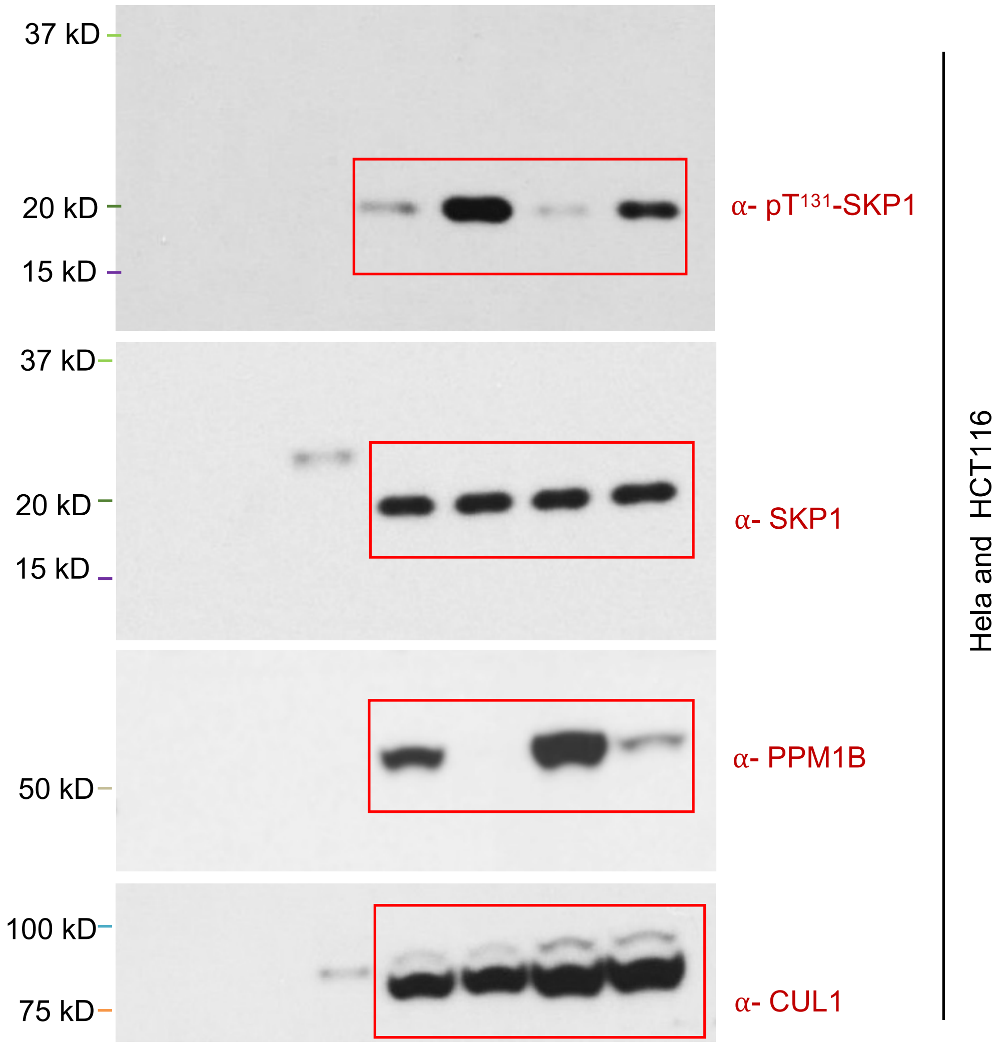
37 kD
20 kD
15 kD
α- pT131-SKP1
37 kD
20 kD
15 kD
α- SKP1
50 kD
α- PPM1B
100 kD
75 kD
α- CUL1
Hela and HCT116

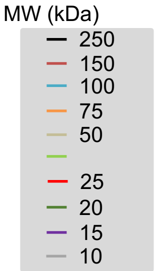
MW (kDa)
250
150
100
75
50
25
20
15
10

1H-2

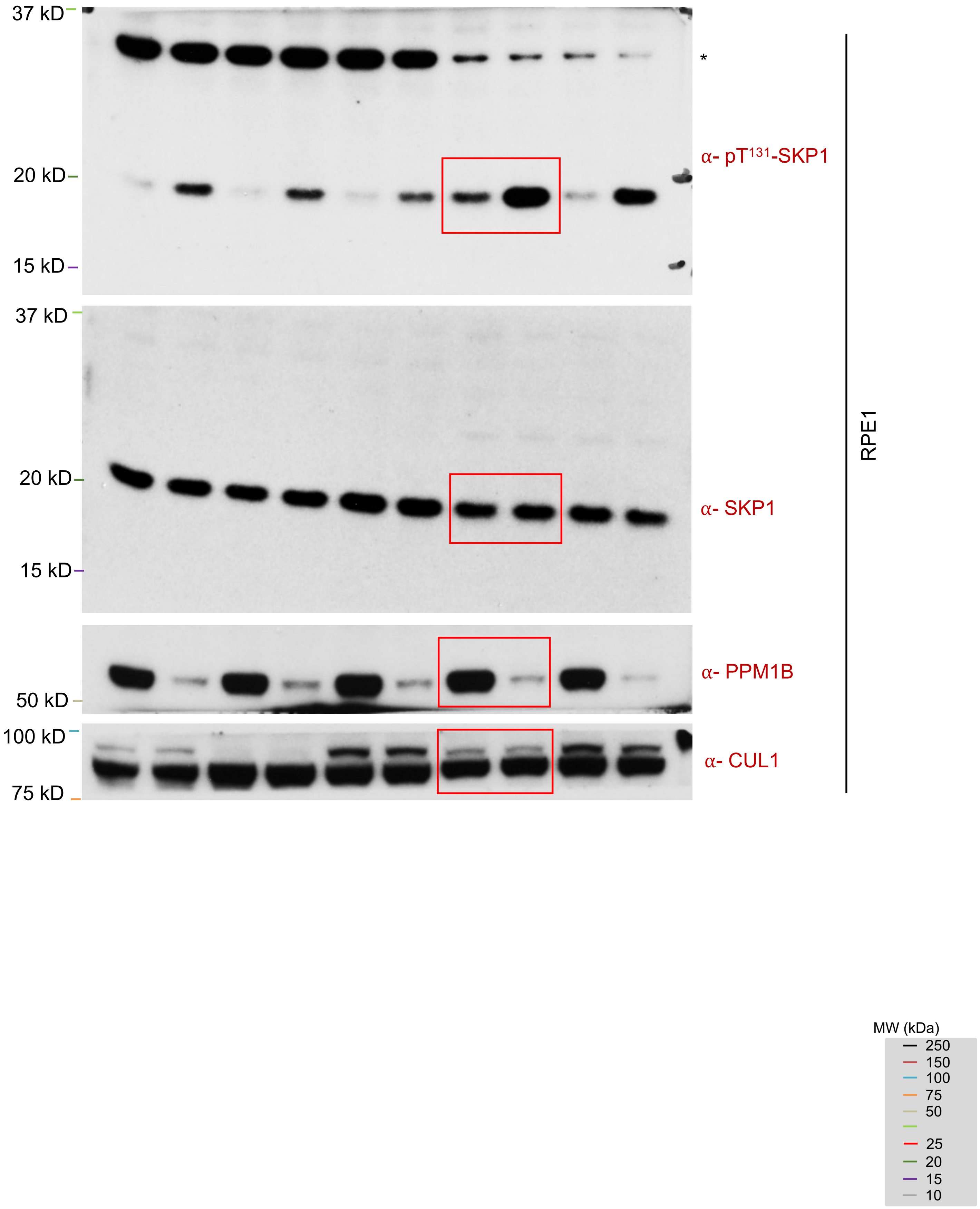

37 kD
20 kD
15 kD
*
α- pT131-SKP1
37 kD
20 kD
15 kD
α- SKP1
50 kD
α- PPM1B
100 kD
75 kD
α- CUL1
RPE1
MW (kDa)
250
150
100
75
50
25
20
15
10

1I

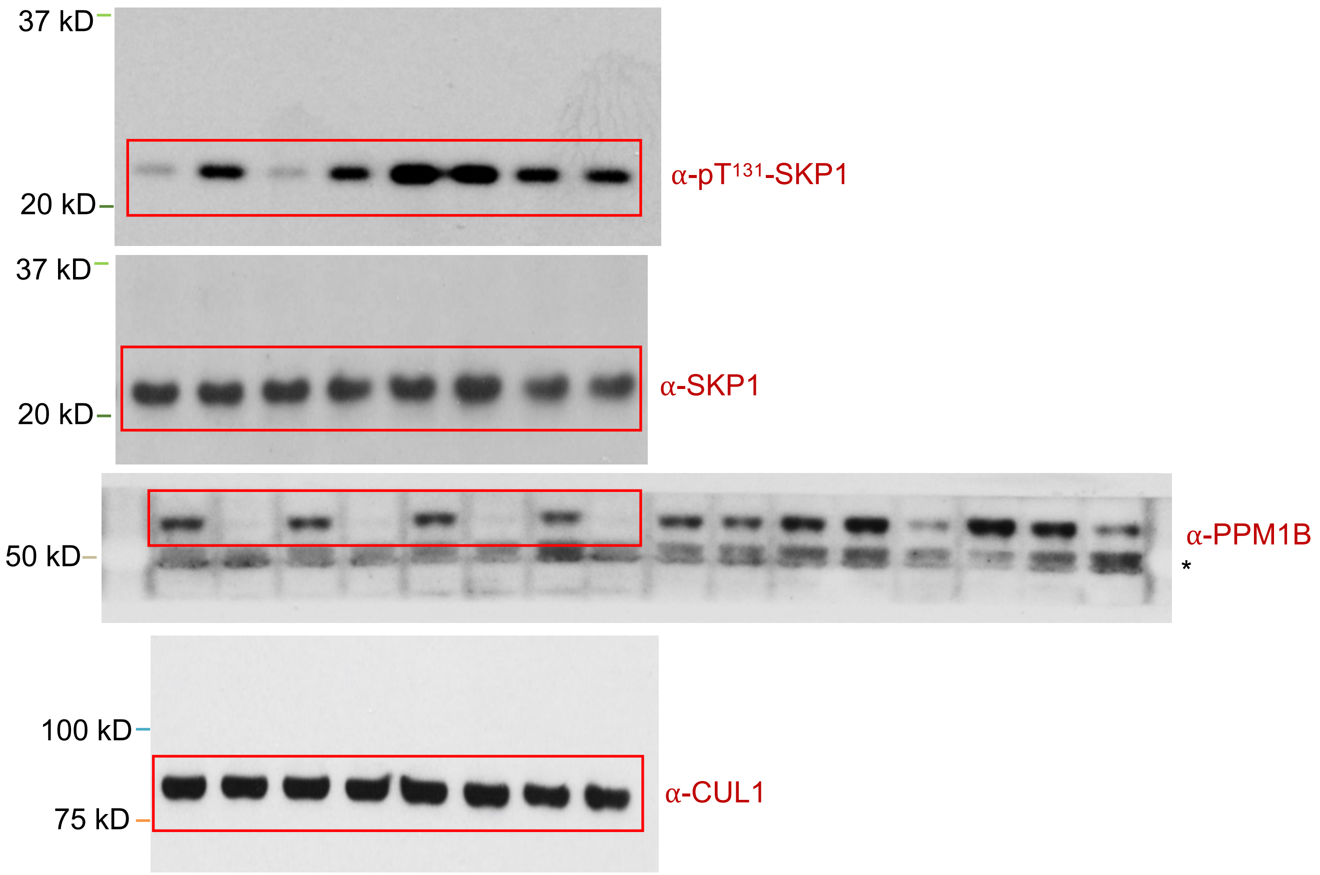
37 kD
20 kD
α-pT131-SKP1
37 kD
20 kD
α-SKP1
50 kD
α-PPM1B
*
100 kD
75 kD
α-CUL1

2B

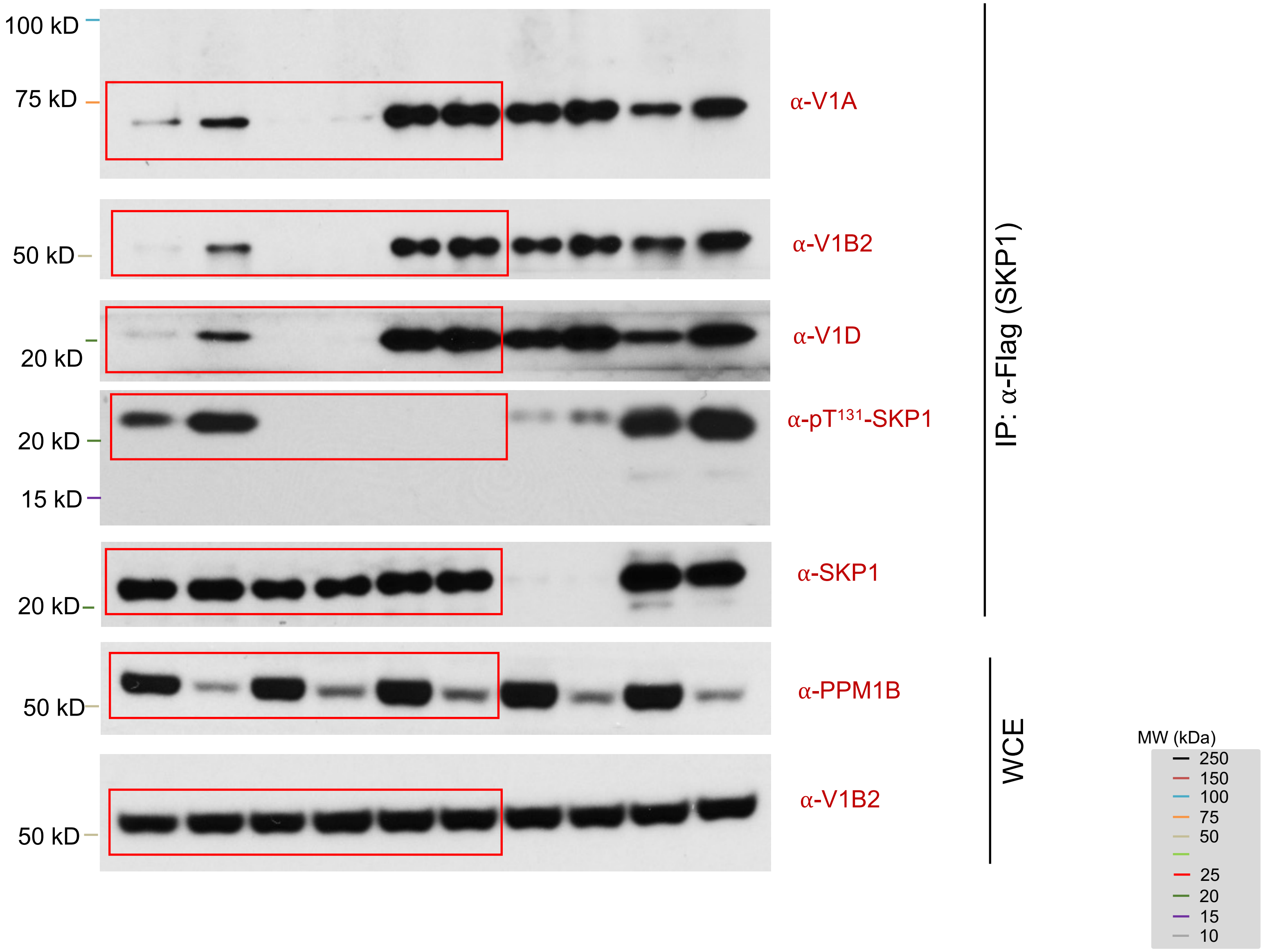
100 kD
75 kD
α-V1A
50 kD
α-V1B2
20 kD
α-V1D
20 kD
15 kD
α-pT131-SKP1
20 kD
α-SKP1
IP: α-Flag (SKP1)
50 kD
α-PPM1B
50 kD
α-V1B2
WCE
MW (kDa)
250
150
100
75
50
25
20
15
10

2C

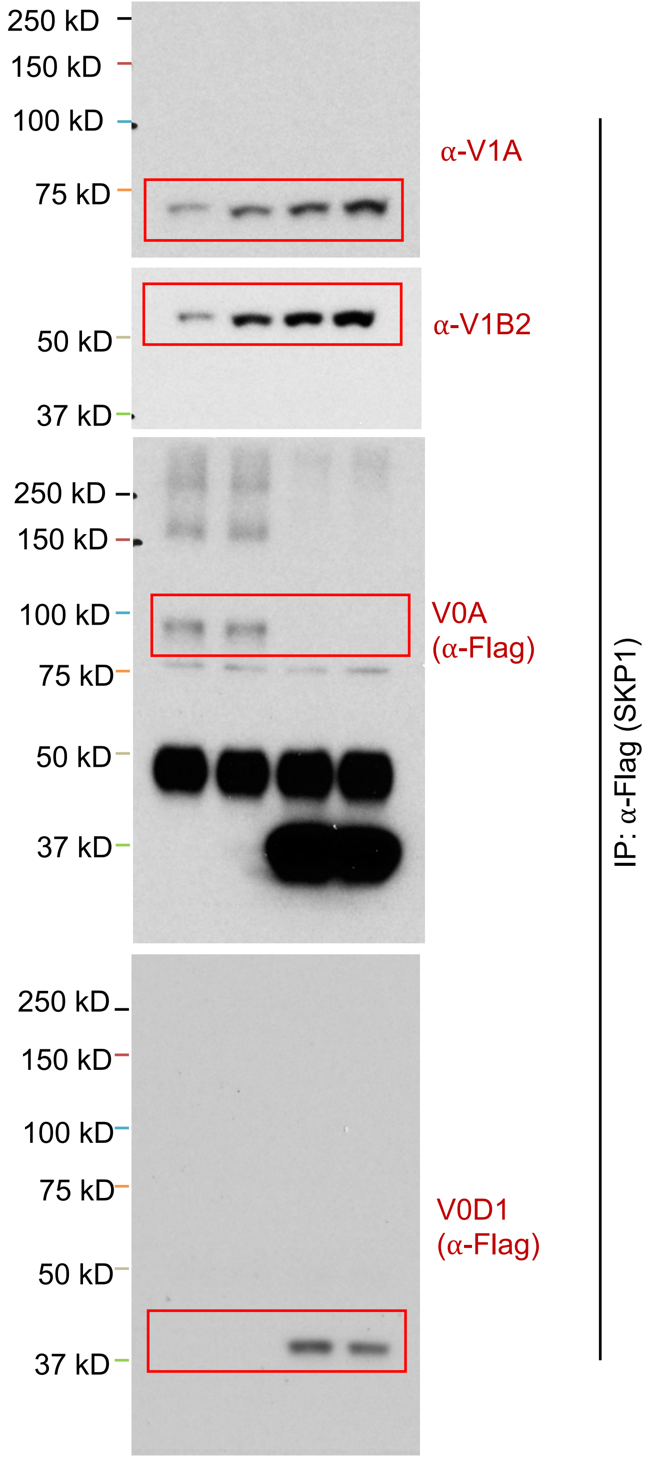
250 kD
150 kD
100 kD
75 kD
α-V1A
α-V1B2
50 kD
37 kD
250 kD
150 kD
100 kD
75 kD
V0A
(α-Flag)
50 kD
37 kD
250 kD
150 kD
100 kD
75 kD
V0D1
(α-Flag)
50 kD
37 kD
IP: α-Flag (SKP1)

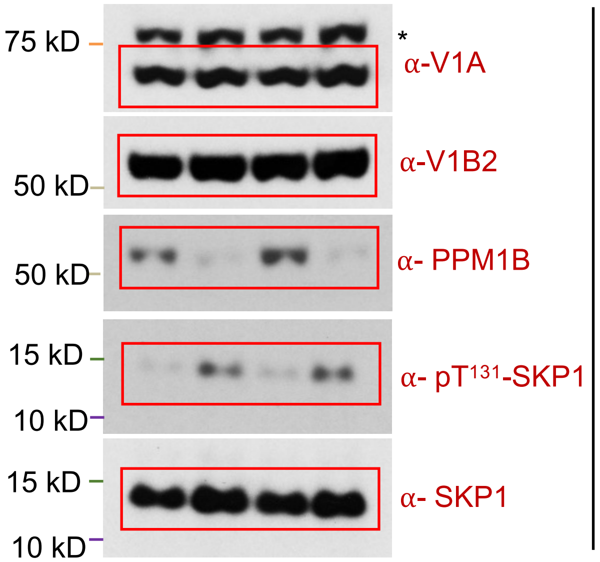
75 kD
*
α-V1A
50 kD
α-V1B2
50 kD
α- PPM1B
15 kD
α- pT131-SKP1
10 kD
15 kD
α- SKP1
10 kD
WCE

2D

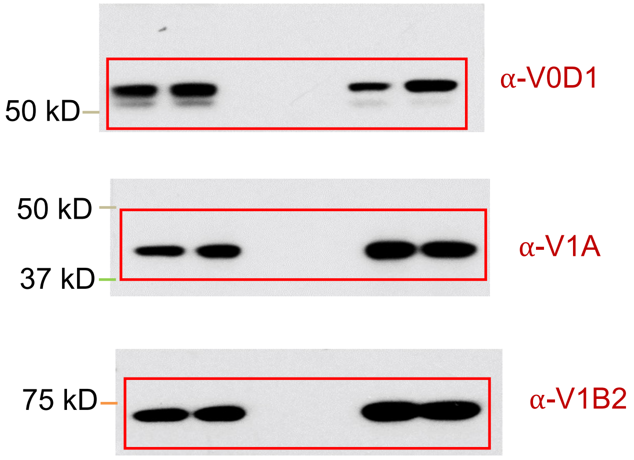
50 kD
α-V0D1
50 kD
37 kD
α-V1A
75 kD
α-V1B2

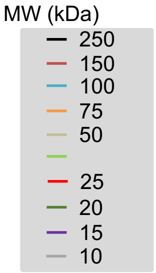
MW (kDa)
250
150
100
75
50
25
20
15
10

2E

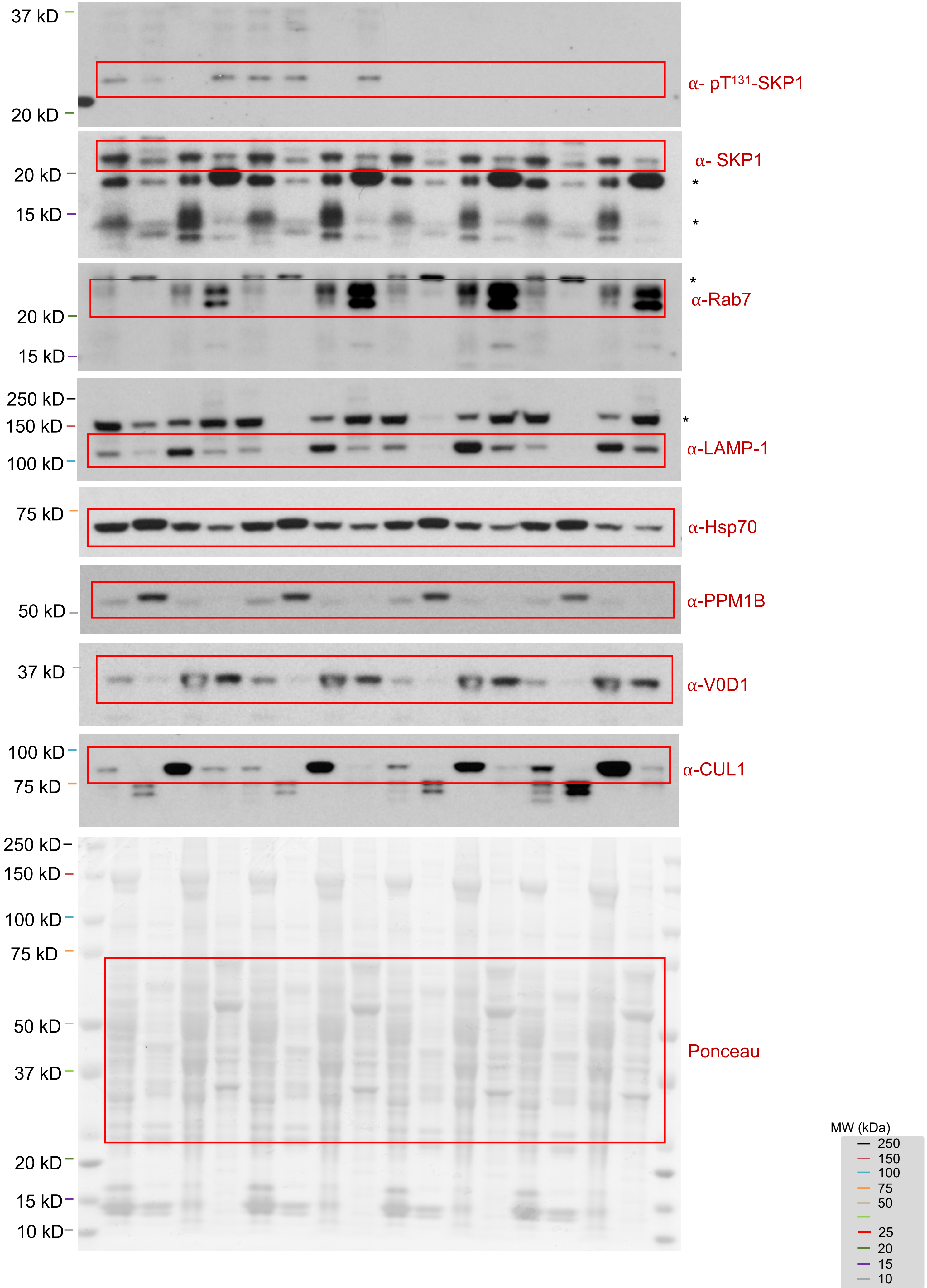
37 kD
20 kD
α- pT131-SKP1
20 kD
15 kD
α- SKP1
*
*
20 kD
15 kD
*
α-Rab7
250 kD
150 kD
100 kD
*
α-LAMP-1
75 kD
α-Hsp70
50 kD
α-PPM1B
37 kD
α-V0D1
100 kD
75 kD
α-CUL1
250 kD
150 kD
100 kD
75 kD
50 kD
37 kD
20 kD
15 kD
10 kD
Ponceau
MW (kDa)
250
150
100
75
50
25
20
15
10

2F

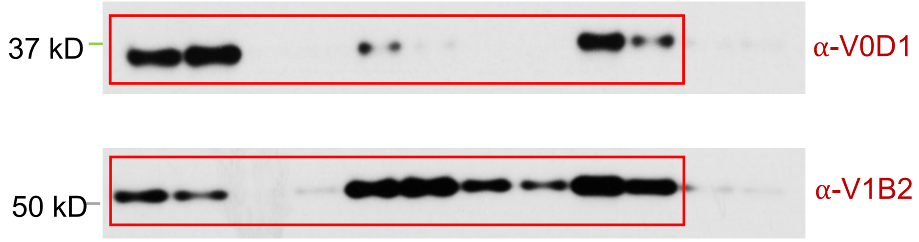
37 kD
α-V0D1
50 kD
α-V1B2

3A

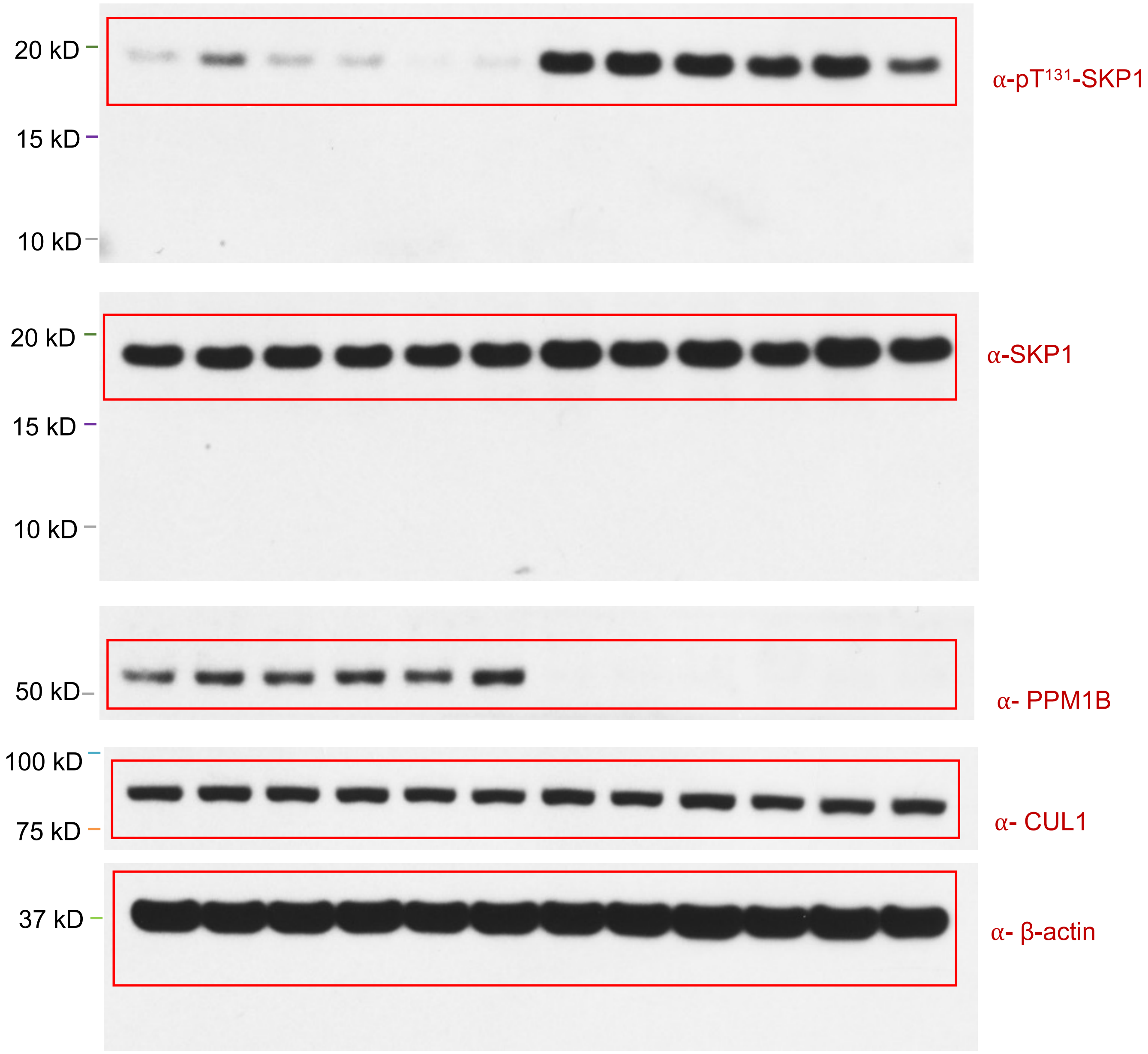
20 kD
15 kD
10 kD
α-pT131-SKP1
20 kD
15 kD
10 kD
α-SKP1
50 kD
α- PPM1B
100 kD
75 kD
α- CUL1
37 kD
α- β-actin

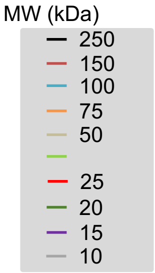
MW (kDa)
250
150
100
75
50
25
20
15
10

3C

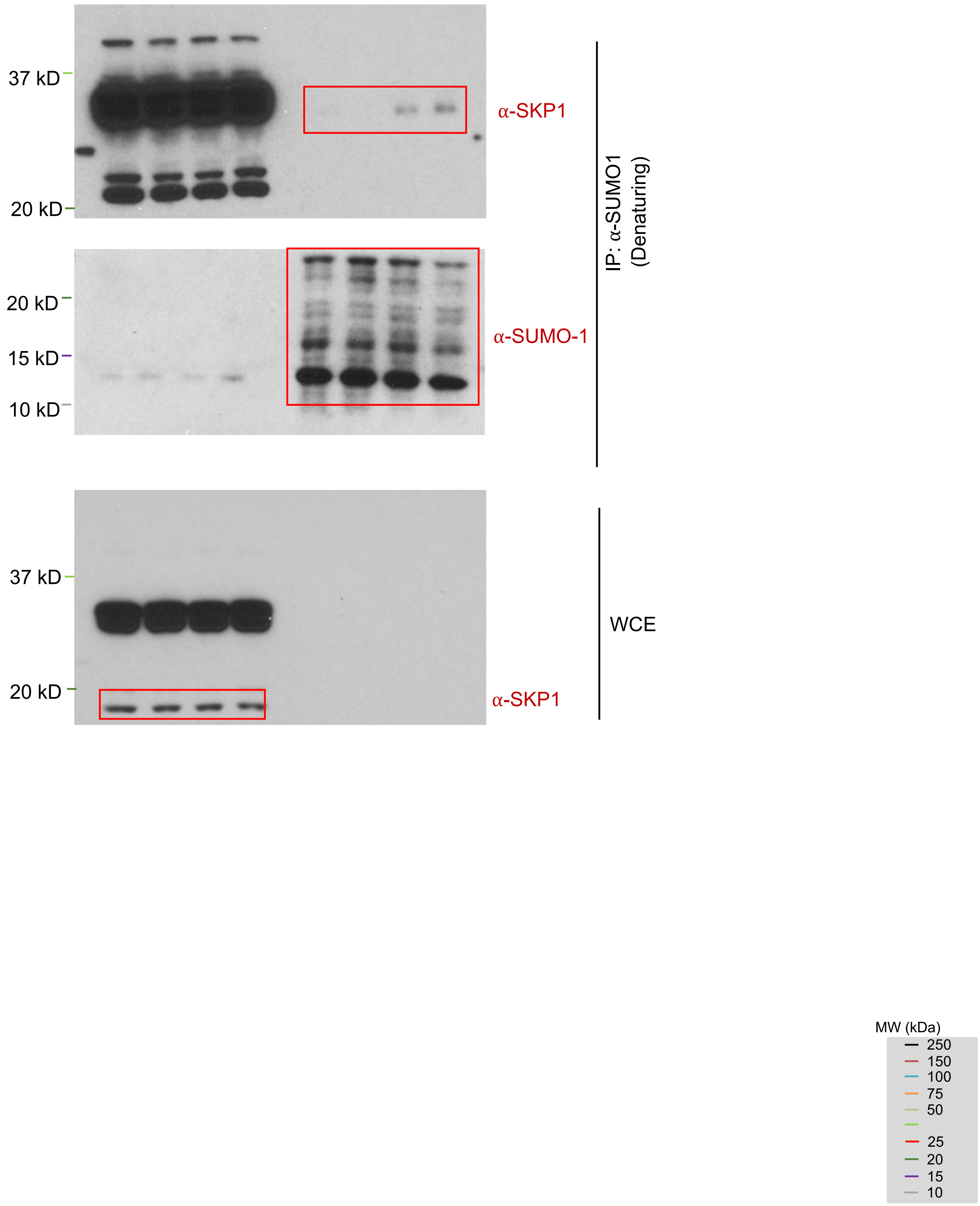
37 kD
20 kD
α-SKP1
20 kD
15 kD
10 kD
α-SUMO-1
IP: α-SUMO1
(Denaturing)
37 kD
20 kD
WCE
α-SKP1
MW (kDa)
250
150
100
75
50
25
20
15
10

3D

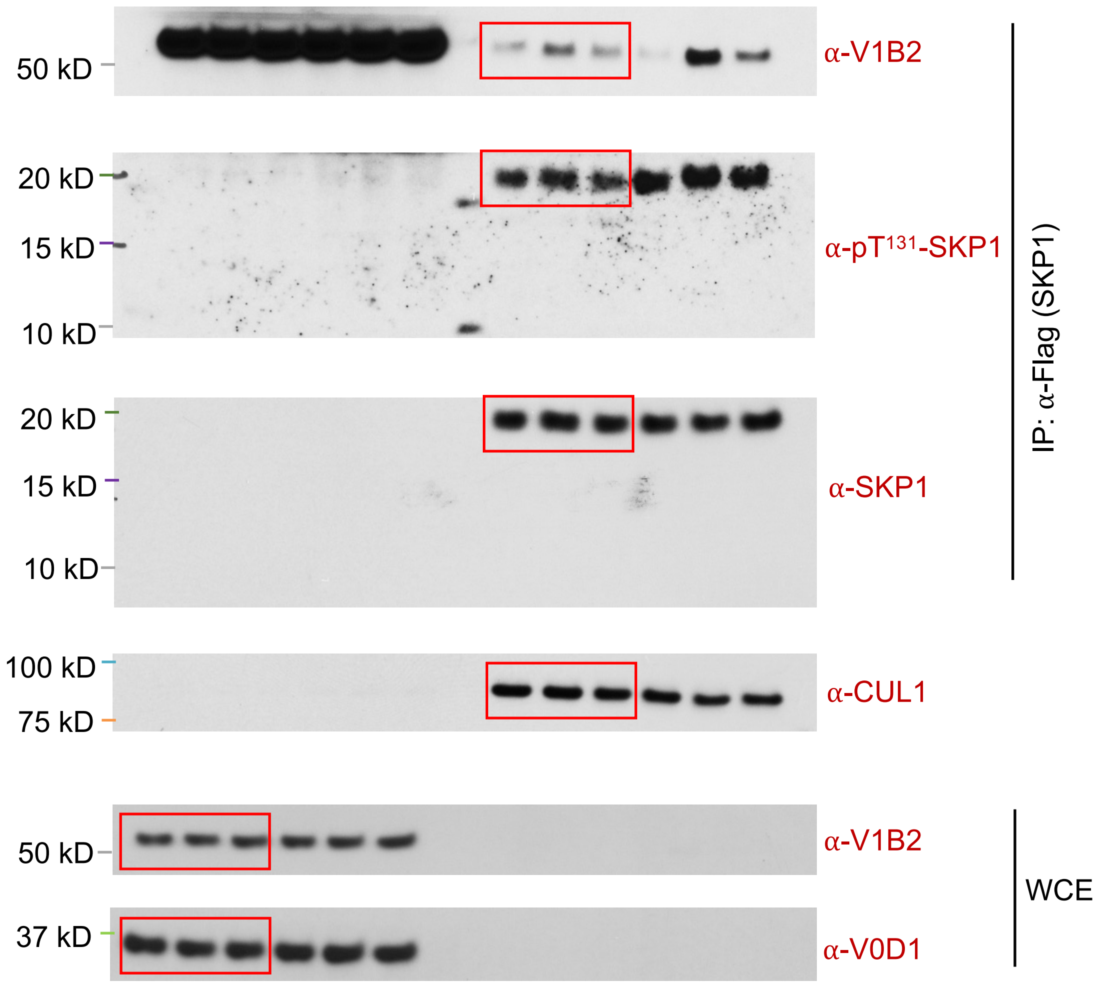

50 kD
α-V1B2
20 kD
15 kD
10 kD
α-pT131-SKP1
20 kD
15 kD
10 kD
α-SKP1
IP: α-Flag (SKP1)
100 kD
75 kD
α-CUL1
50 kD
α-V1B2
37 kD
α-V0D1
WCE

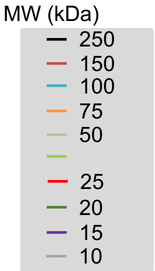

MW (kDa)
250
150
100
75
50
25
20
15
10

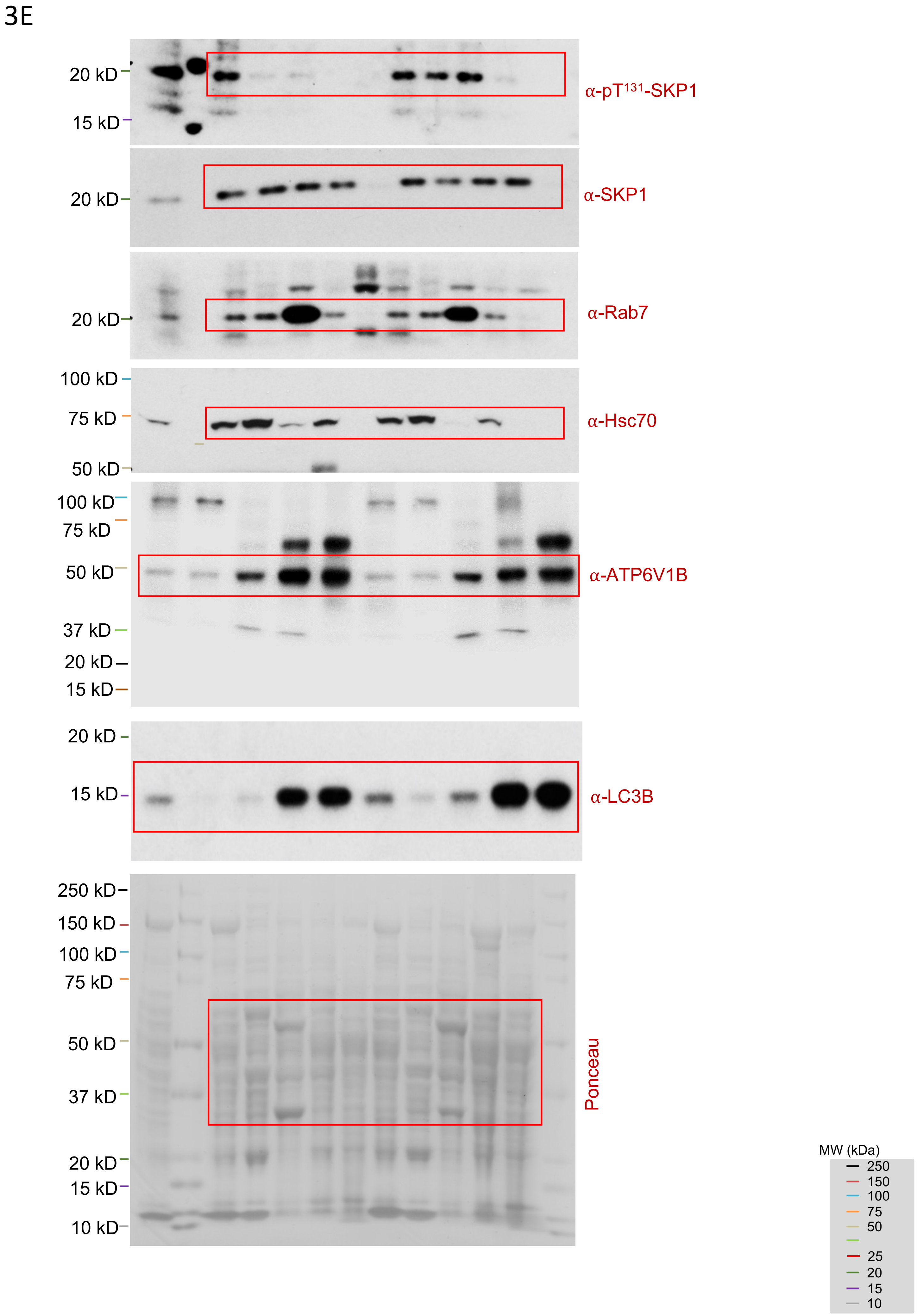
3E
20 kD
15 kD
α-pT131-SKP1
20 kD
α-SKP1
20 kD
α-Rab7
100 kD
75 kD
50 kD
α-Hsc70
100 kD
75 kD
50 kD
37 kD
20 kD
15 kD
α-ATP6V1B
20 kD
15 kD
α-LC3B
250 kD
150 kD
100 kD
75 kD
50 kD
37 kD
20 kD
15 kD
10 kD
Ponceau
MW (kDa)
250
150
100
75
50
25
20
15
10

4A

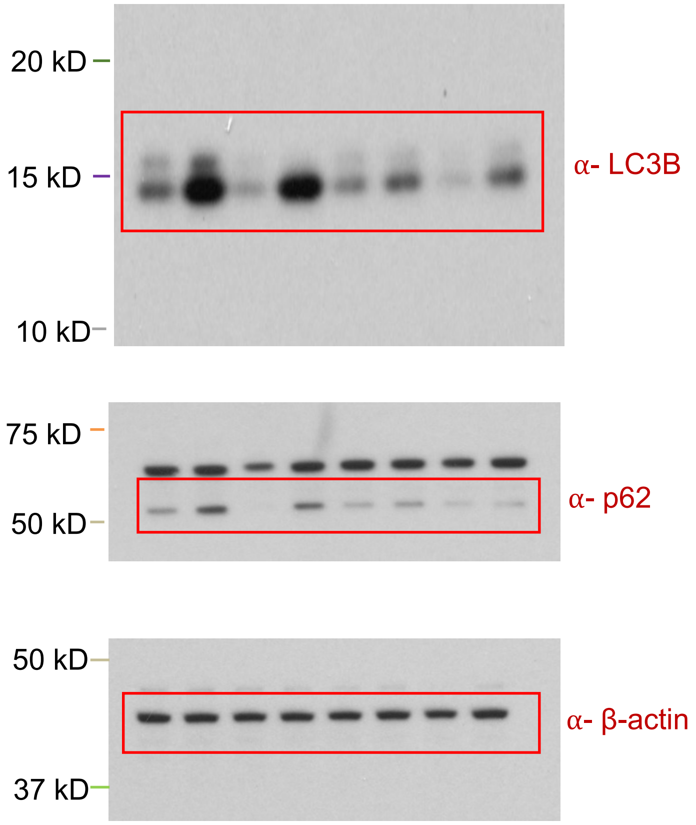
20 kD
15 kD
10 kD
α- LC3B
75 kD
50 kD
α- p62
50 kD
37 kD
α- β-actin

4C

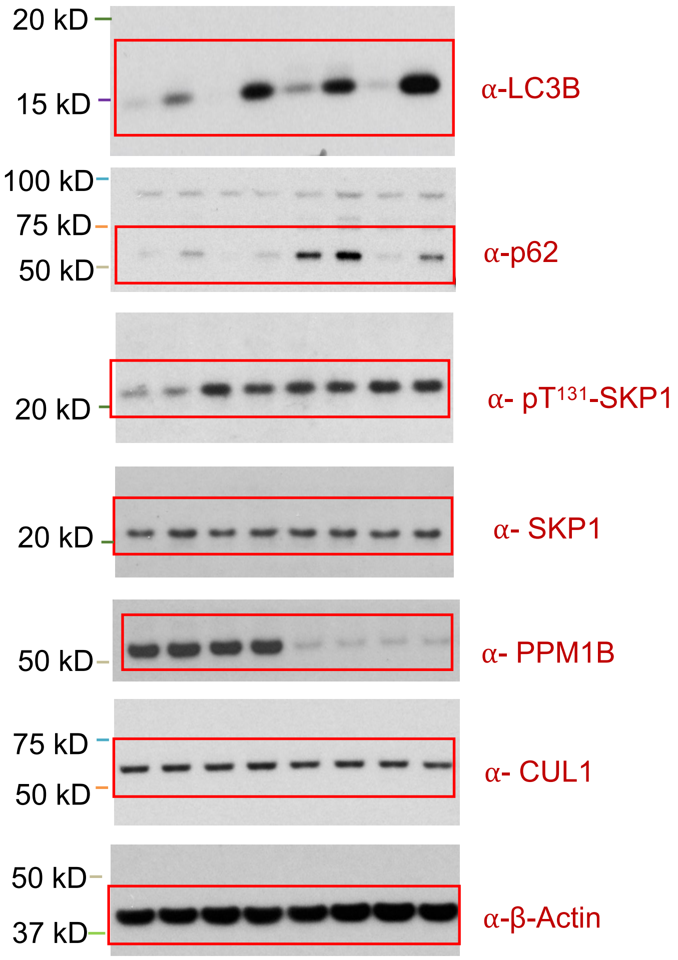
20 kD
15 kD
α-LC3B
100 kD
75 kD
50 kD
α-p62
20 kD
α- pT131-SKP1
20 kD
α- SKP1
50 kD
α- PPM1B
75 kD
50 kD
α- CUL1
50 kD
37 kD
α-β-Actin

5A

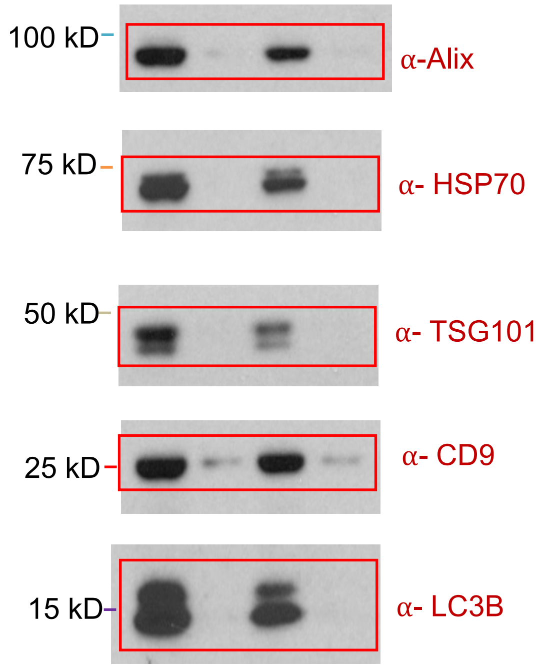
100 kD
α-Alix
75 kD
α- HSP70
50 kD
α- TSG101
25 kD
α- CD9
15 kD
α- LC3B

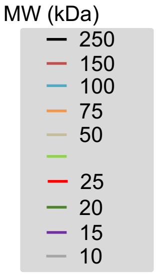
MW (kDa)
250
150
100
75
50
25
20
15
10

5E

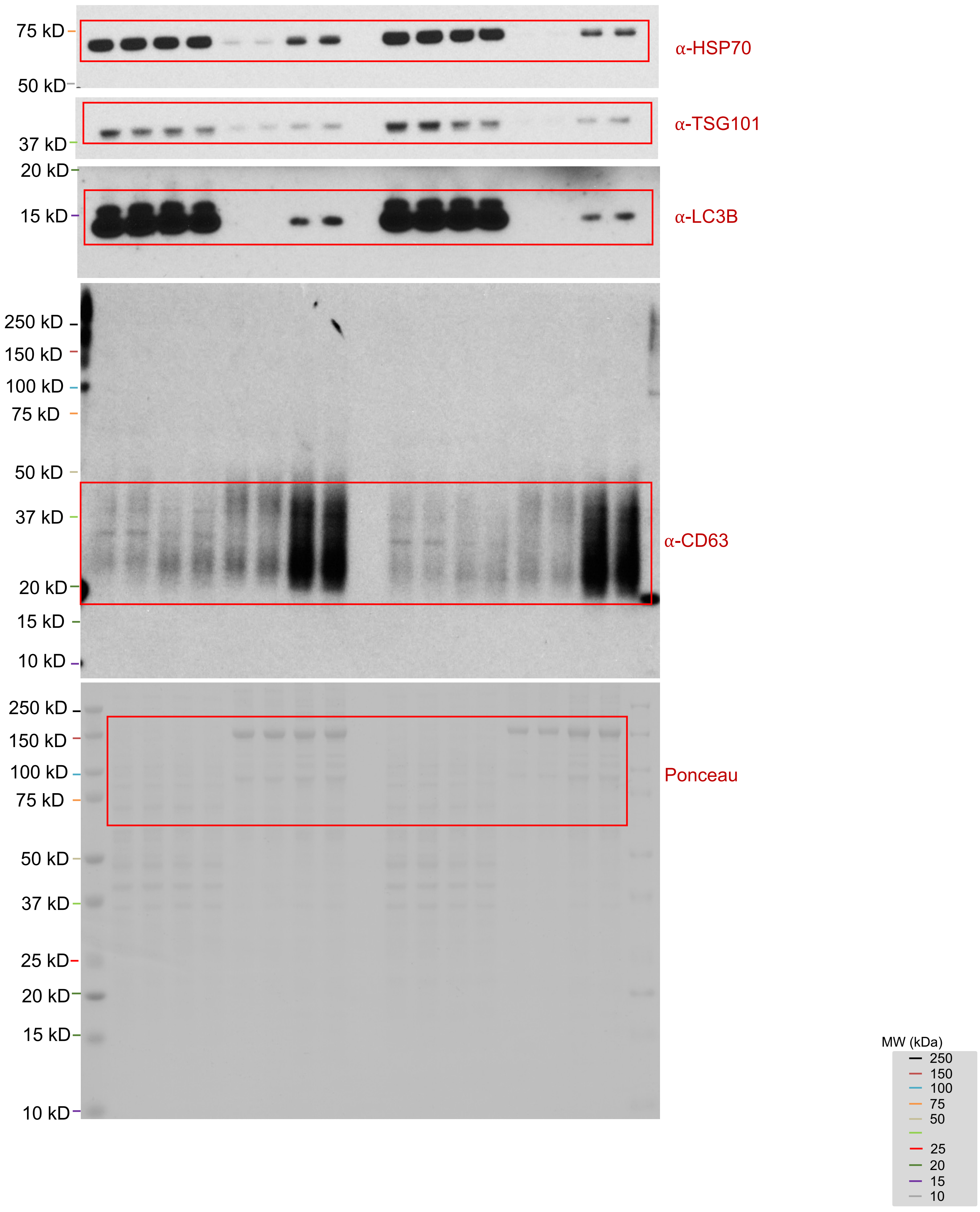

75 kD
50 kD
α-HSP70
α-TSG101
37 kD
20 kD
15 kD
α-LC3B
250 kD
150 kD
100 kD
75 kD
50 kD
37 kD
20 kD
15 kD
10 kD
α-CD63
250 kD
150 kD
100 kD
75 kD
50 kD
37 kD
25 kD
20 kD
15 kD
10 kD
Ponceau
MW (kDa)
250
150
100
75
50
25
20
15
10

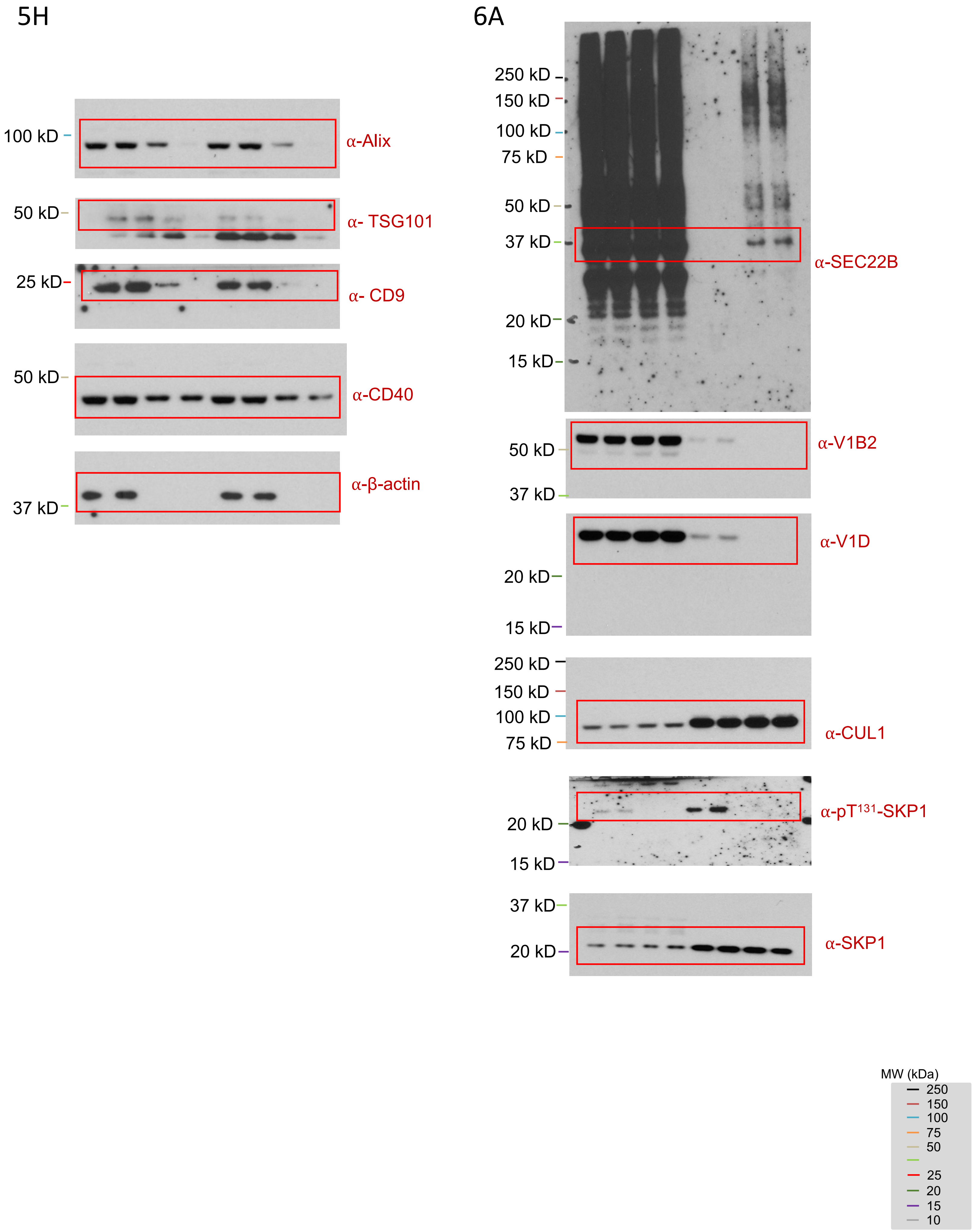
5H
100 kD
α-Alix
50 kD
α- TSG101
25 kD
α- CD9
50 kD
α-CD40
37 kD
α-β-actin
6A
250 kD
150 kD
100 kD
75 kD
50 kD
37 kD
20 kD
15 kD
α-SEC22B
50 kD
37 kD
α-V1B2
20 kD
15 kD
α-V1D
250 kD
150 kD
100 kD
75 kD
α-CUL1
20 kD
15 kD
α-pT131-SKP1
37 kD
20 kD
α-SKP1
MW (kDa)
250
150
100
75
50
25
20
15
10

6B

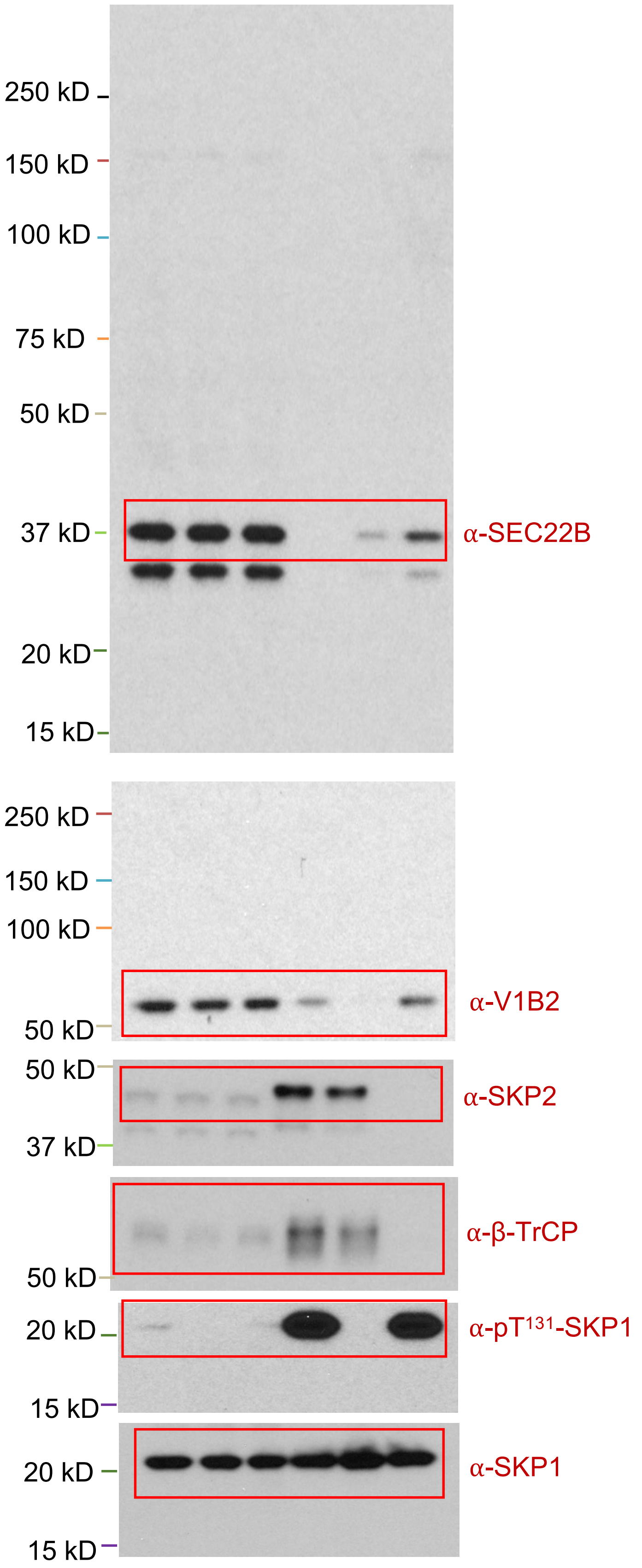
250 kD
150 kD
100 kD
75 kD
50 kD
37 kD
20 kD
15 kD
α-SEC22B
250 kD
150 kD
100 kD
50 kD
α-V1B2
50 kD
37 kD
α-SKP2
α-β-TrCP
50 kD
20 kD
15 kD
α-pT131-SKP1
20 kD
15 kD
α-SKP1

6C

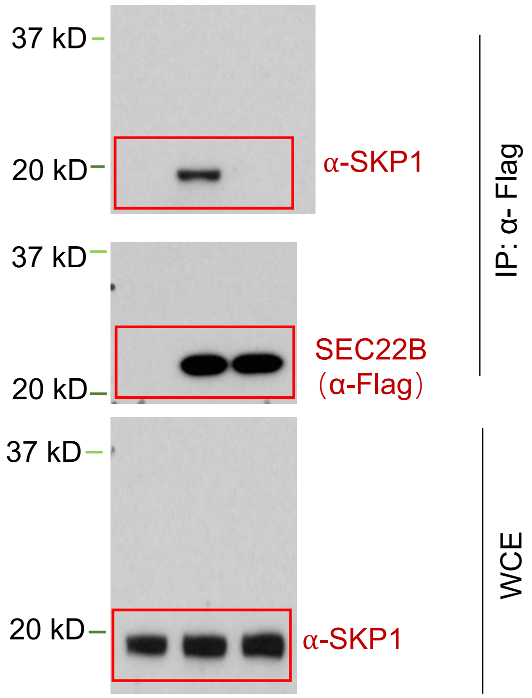
37 kD
20 kD
α-SKP1
37 kD
20 kD
SEC22B
(α-Flag)
IP: α- Flag
37 kD
20 kD
α-SKP1
WCE

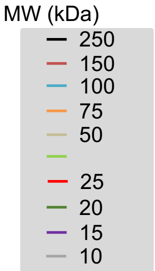
MW (kDa)
250
150
100
75
50
25
20
15
10

6D

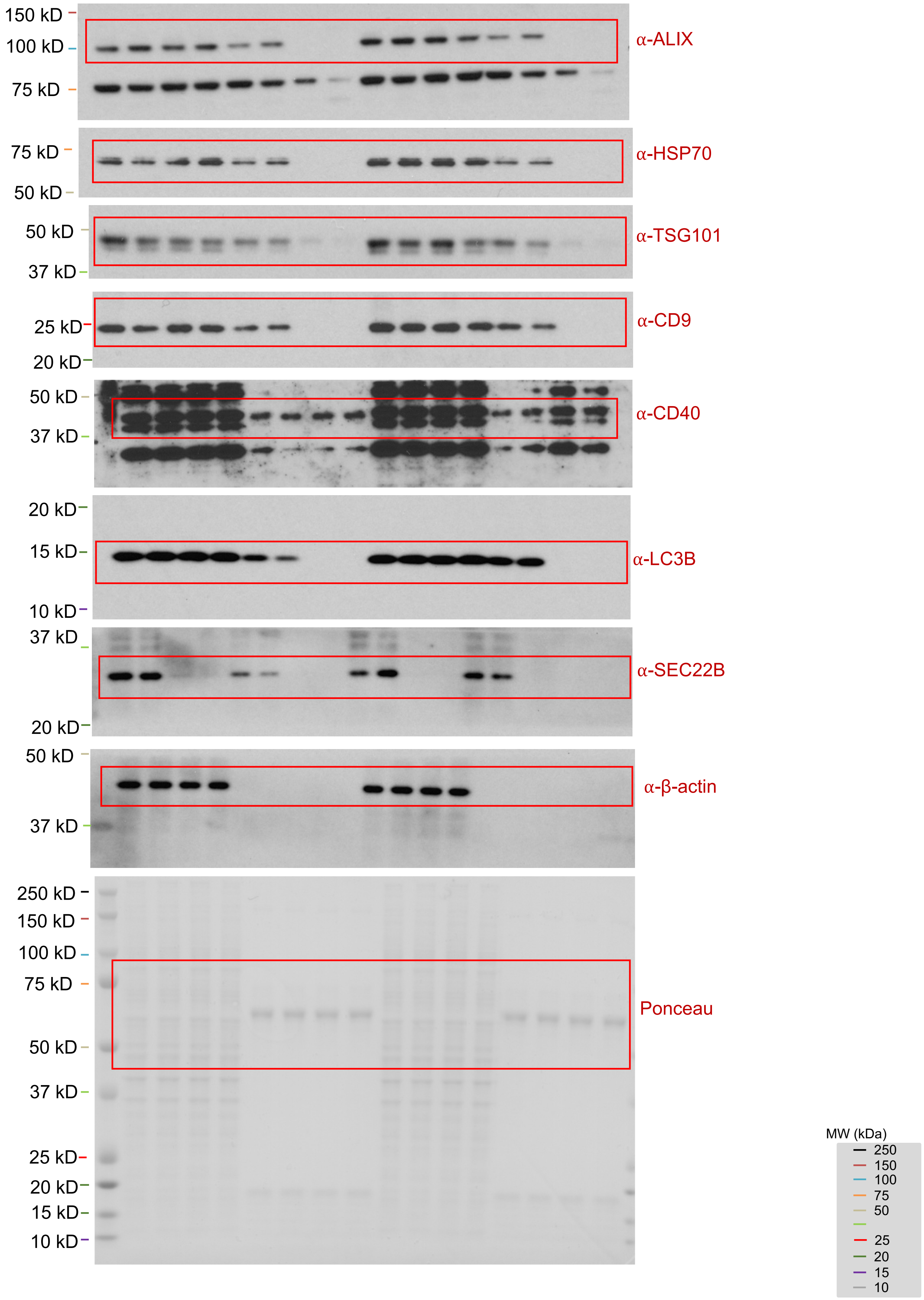
150 kD
100 kD
75 kD
α-ALIX
75 kD
50 kD
α-HSP70
50 kD
37 kD
α-TSG101
25 kD
20 kD
α-CD9
50 kD
37 kD
α-CD40
20 kD
15 kD
10 kD
α-LC3B
37 kD
20 kD
α-SEC22B
50 kD
37 kD
α-β-actin
250 kD
150 kD
100 kD
75 kD
50 kD
37 kD
25 kD
20 kD
15 kD
10 kD
Ponceau
MW (kDa)
250
150
100
75
50
25
20
15
10

S1A

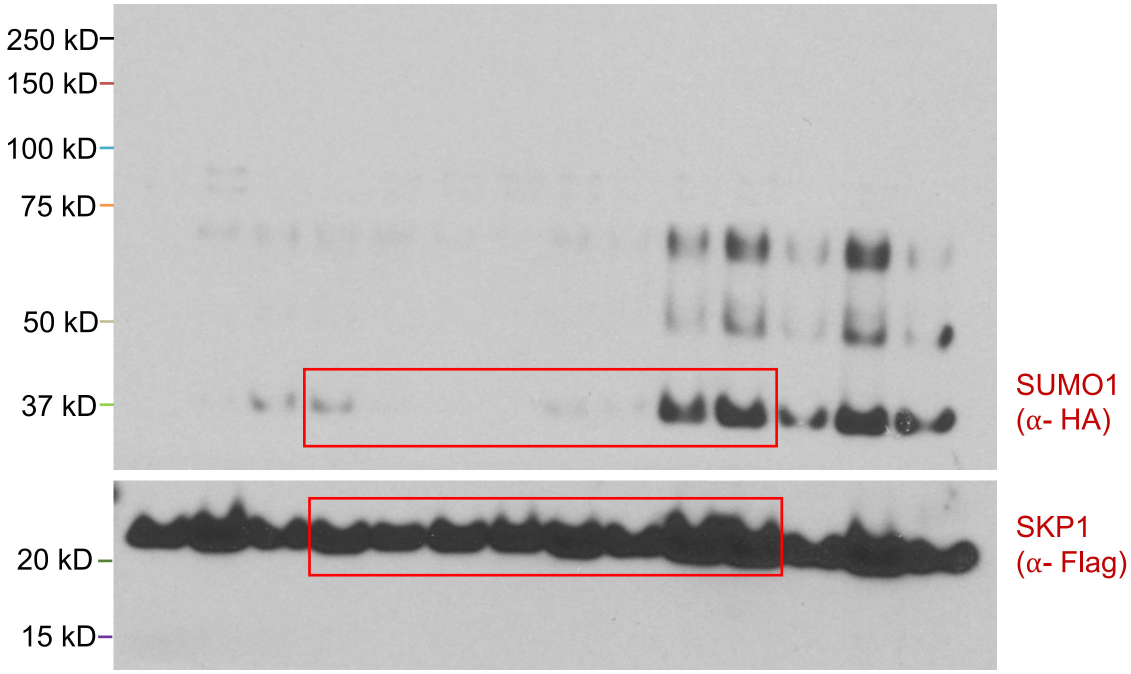
250 kD
150 kD
100 kD
75 kD
50 kD
37 kD
20 kD
15 kD
SUMO1
(α- HA)
SKP1
(α- Flag)

S1B

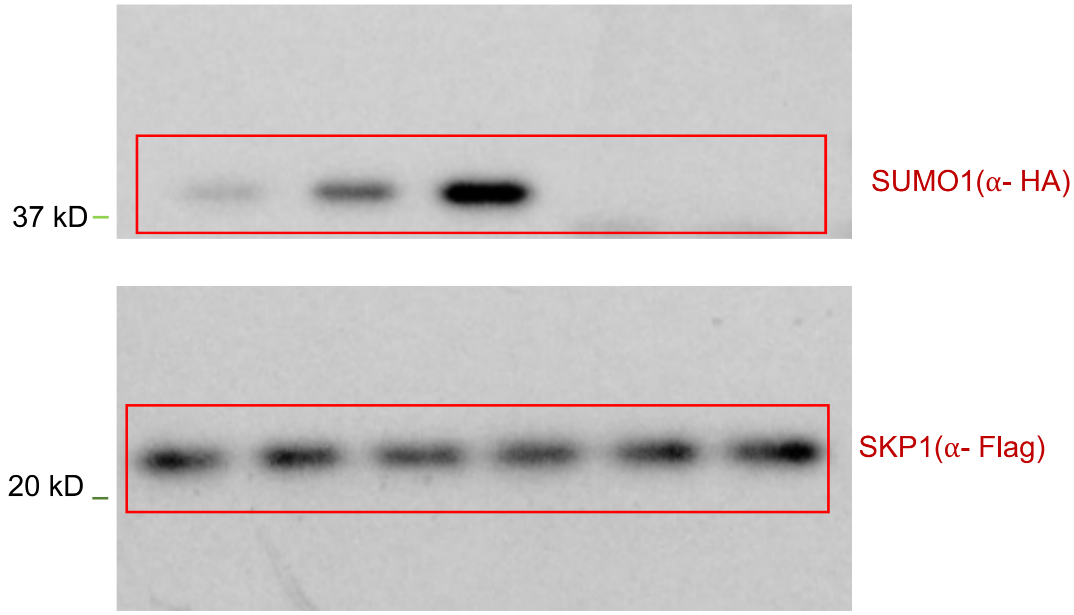
37 kD
20 kD
SUMO1(α- HA)
SKP1(α- Flag)

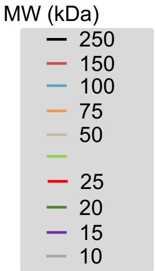
MW (kDa)
250
150
100
75
50
25
20
15
10

S1D

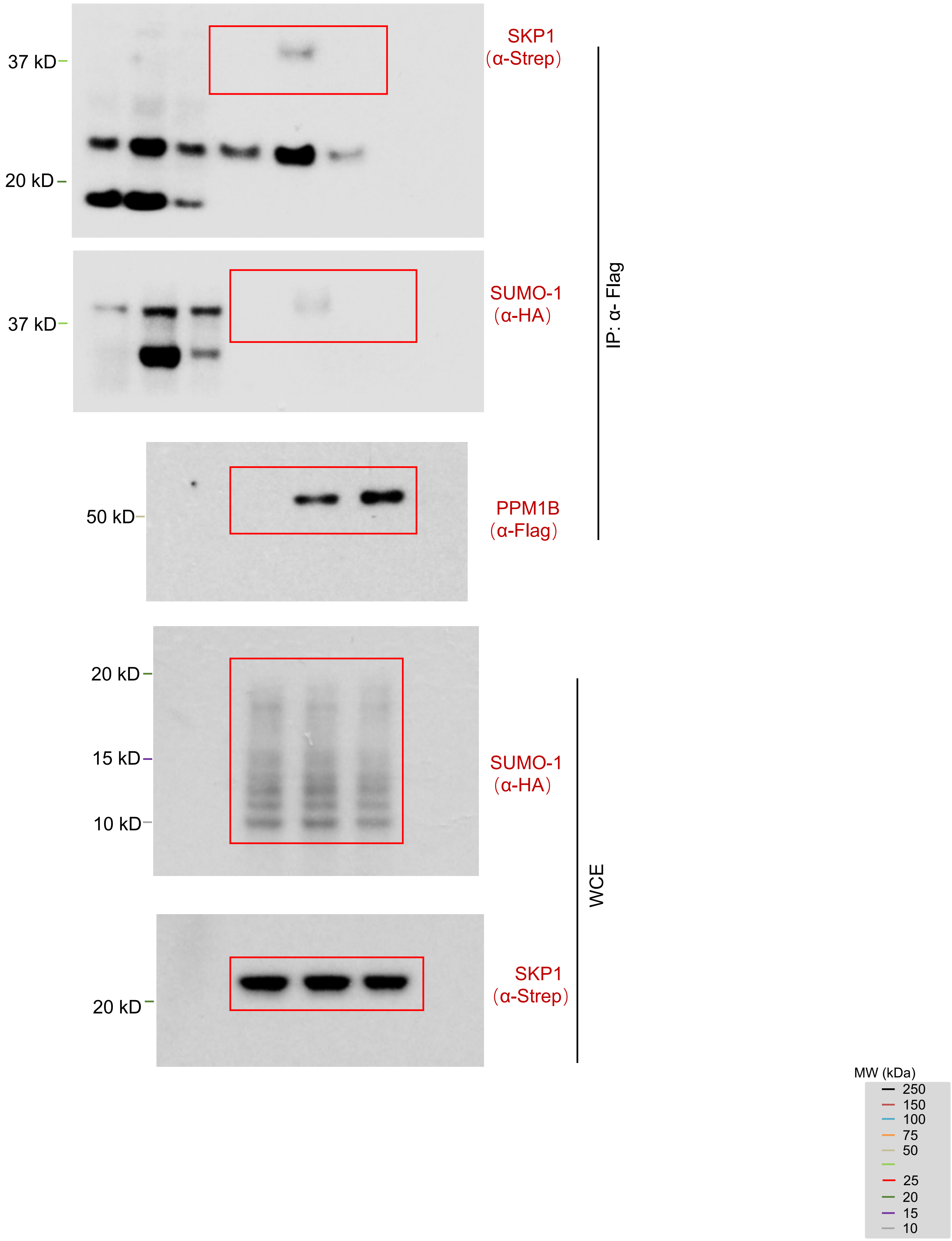
37 kD
20 kD
SKP1
(α-Strep)
37 kD
SUMO-1
(α-HA)
50 kD
PPM1B
(α-Flag)
IP: α- Flag
20 kD
15 kD
10 kD
SUMO-1
(α-HA)
20 kD
SKP1
(α-Strep)
WCE
MW (kDa)
250
150
100
75
50
25
20
15
10

S2C

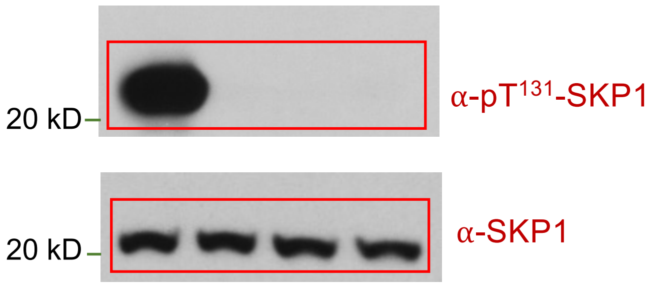
20 kD
α-pT131-SKP1
20 kD
α-SKP1

S2D

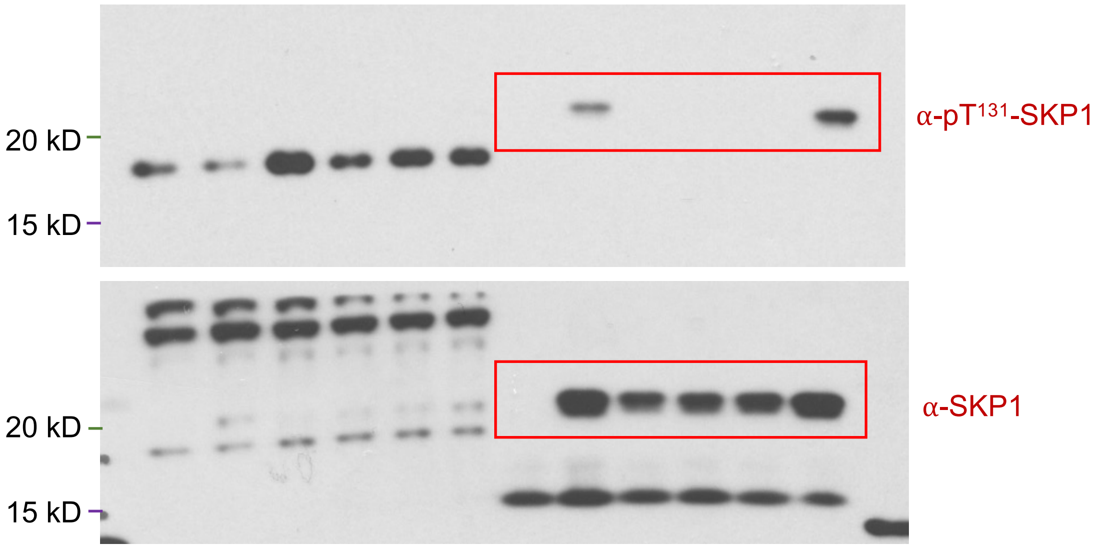
20 kD
15 kD
α-pT131-SKP1
20 kD
15 kD
α-SKP1

S2F

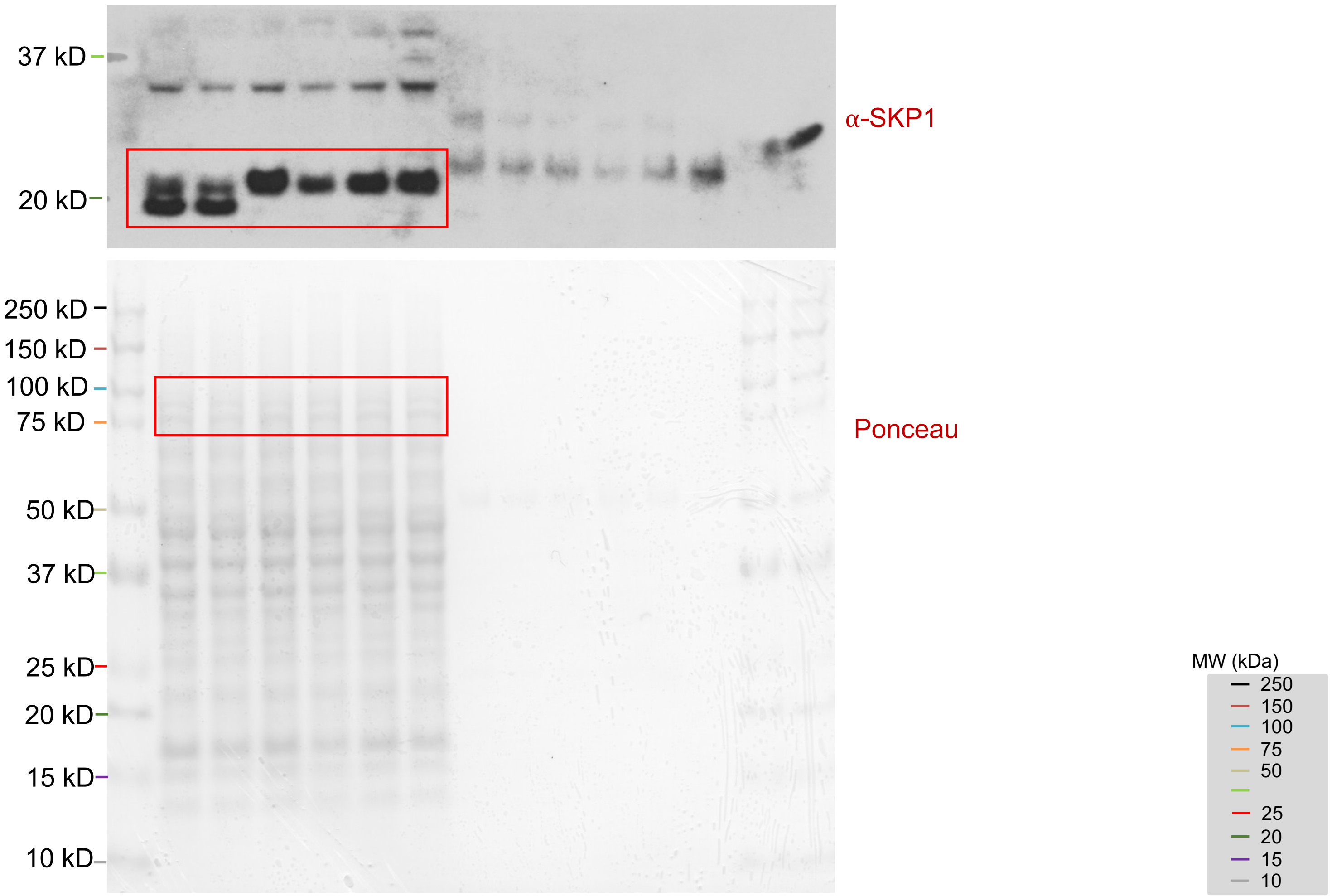
37 kD
20 kD
α-SKP1
250 kD
150 kD
100 kD
75 kD
50 kD
37 kD
25 kD
20 kD
15 kD
10 kD
Ponceau
MW (kDa)
250
150
100
75
50
25
20
15
10

S3A

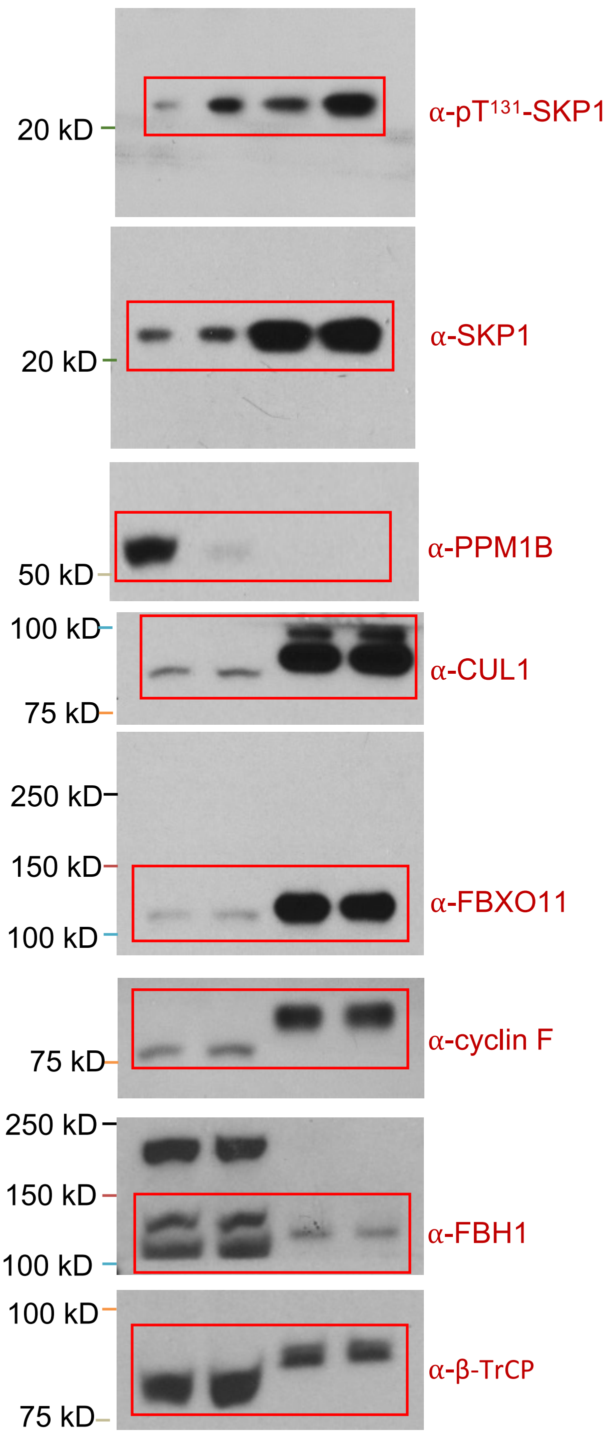
20 kD
α-pT131-SKP1
20 kD
α-SKP1
50 kD
α-PPM1B
100 kD
75 kD
α-CUL1
250 kD
150 kD
100 kD
α-FBXO11
75 kD
α-cyclin F
250 kD
150 kD
100 kD
α-FBH1
100 kD
75 kD
α-β-TrCP

S3B

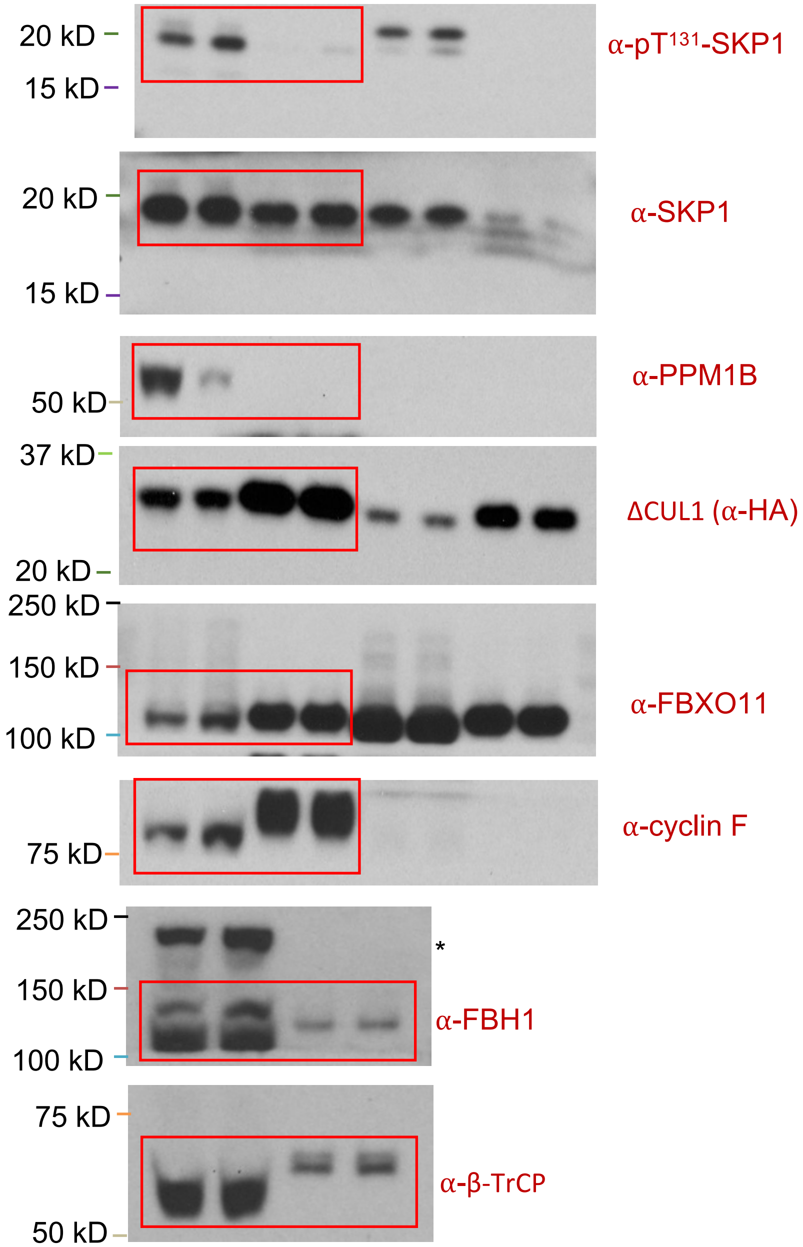
20 kD
15 kD
α-pT131-SKP1
20 kD
15 kD
α-SKP1
50 kD
α-PPM1B
37 kD
20 kD
ΔCUL1 (α-HA)
250 kD
150 kD
100 kD
α-FBXO11
75 kD
α-cyclin F
250 kD
150 kD
100 kD
*
α-FBH1
75 kD
50 kD
α-β-TrCP

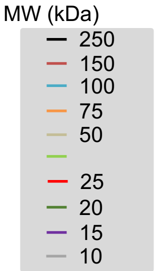
MW (kDa)
250
150
100
75
50
25
20
15
10

S3E

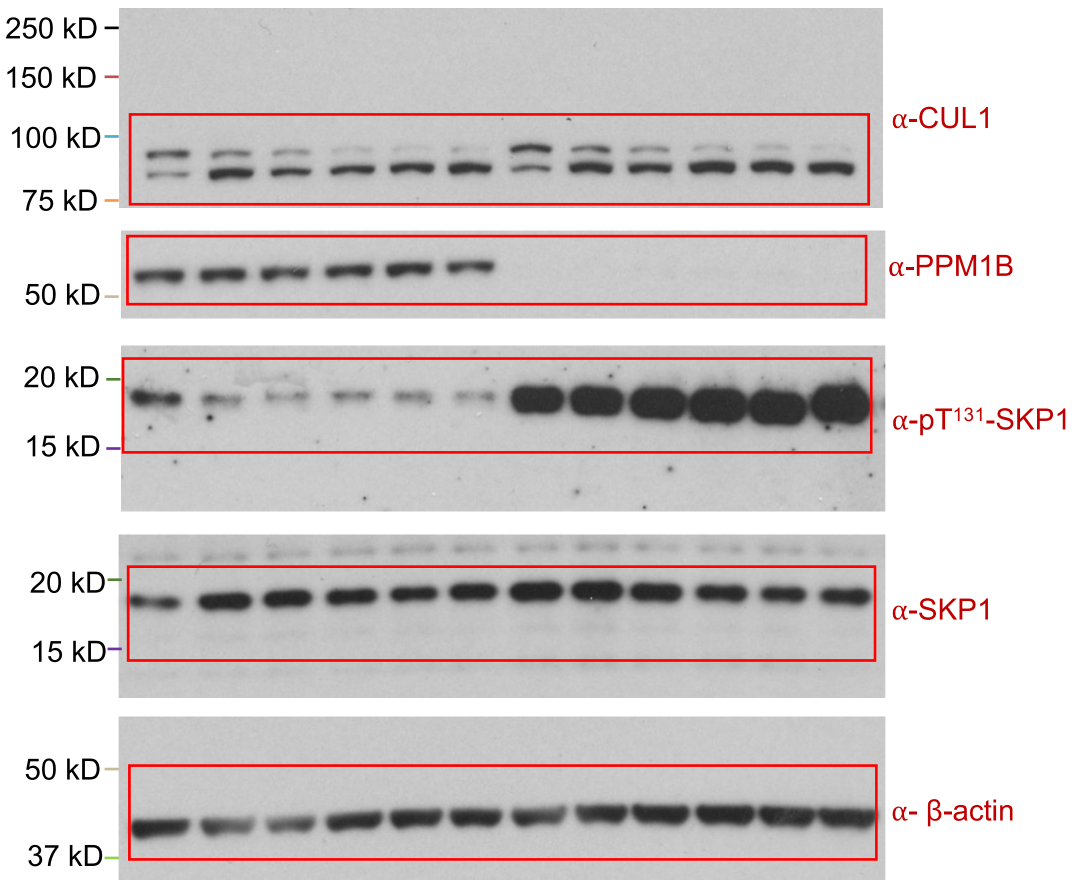
250 kD
150 kD
100 kD
75 kD
α-CUL1
α-PPM1B
50 kD
20 kD
15 kD
α-pT131-SKP1
20 kD
15 kD
α-SKP1
50 kD
37 kD
α- β-actin

S3G

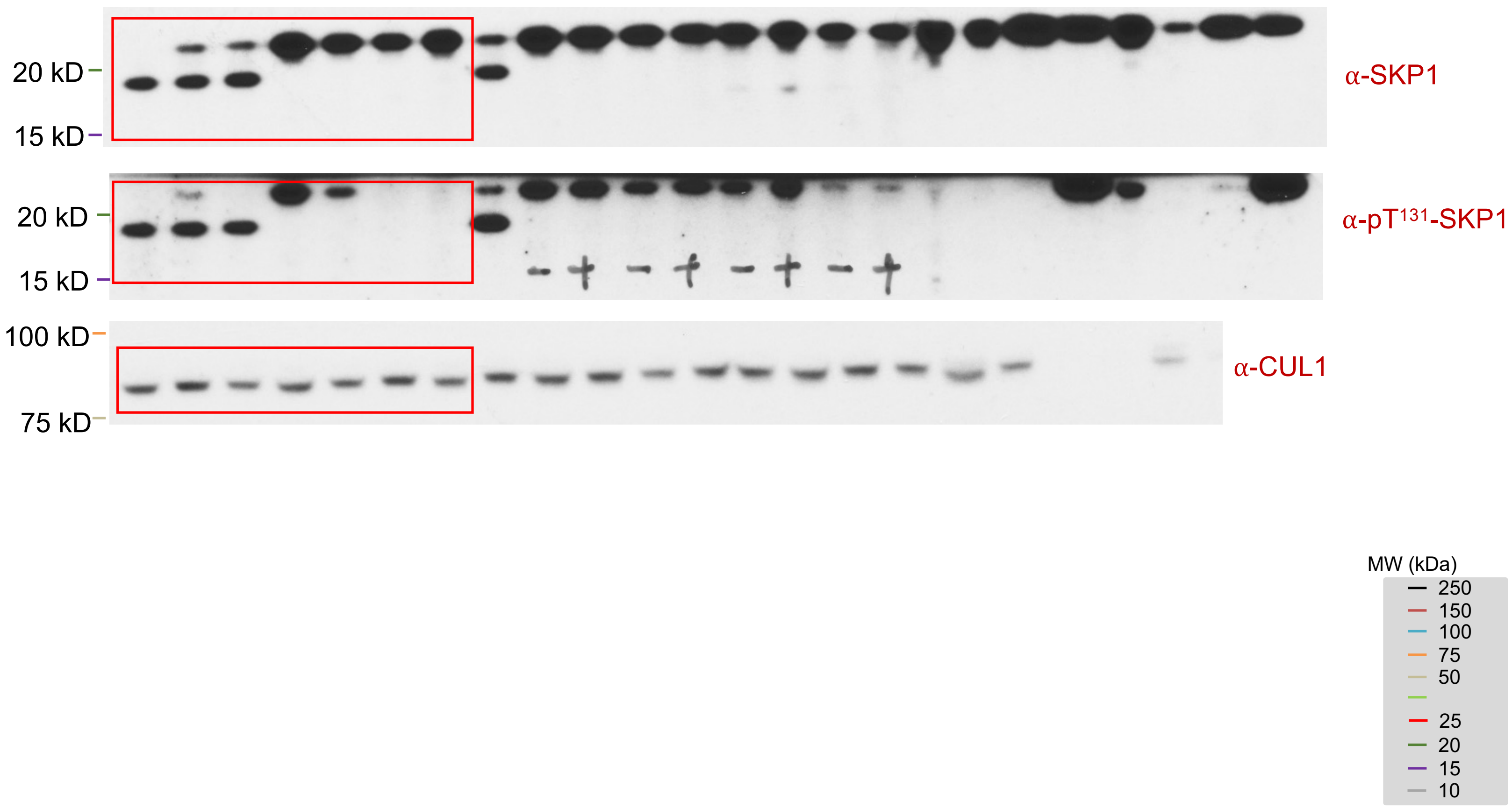
20 kD
15 kD
α-SKP1
20 kD
15 kD
α-pT131-SKP1
100 kD
75 kD
α-CUL1
MW (kDa)
250
150
100
75
50
25
20
15
10

S4A

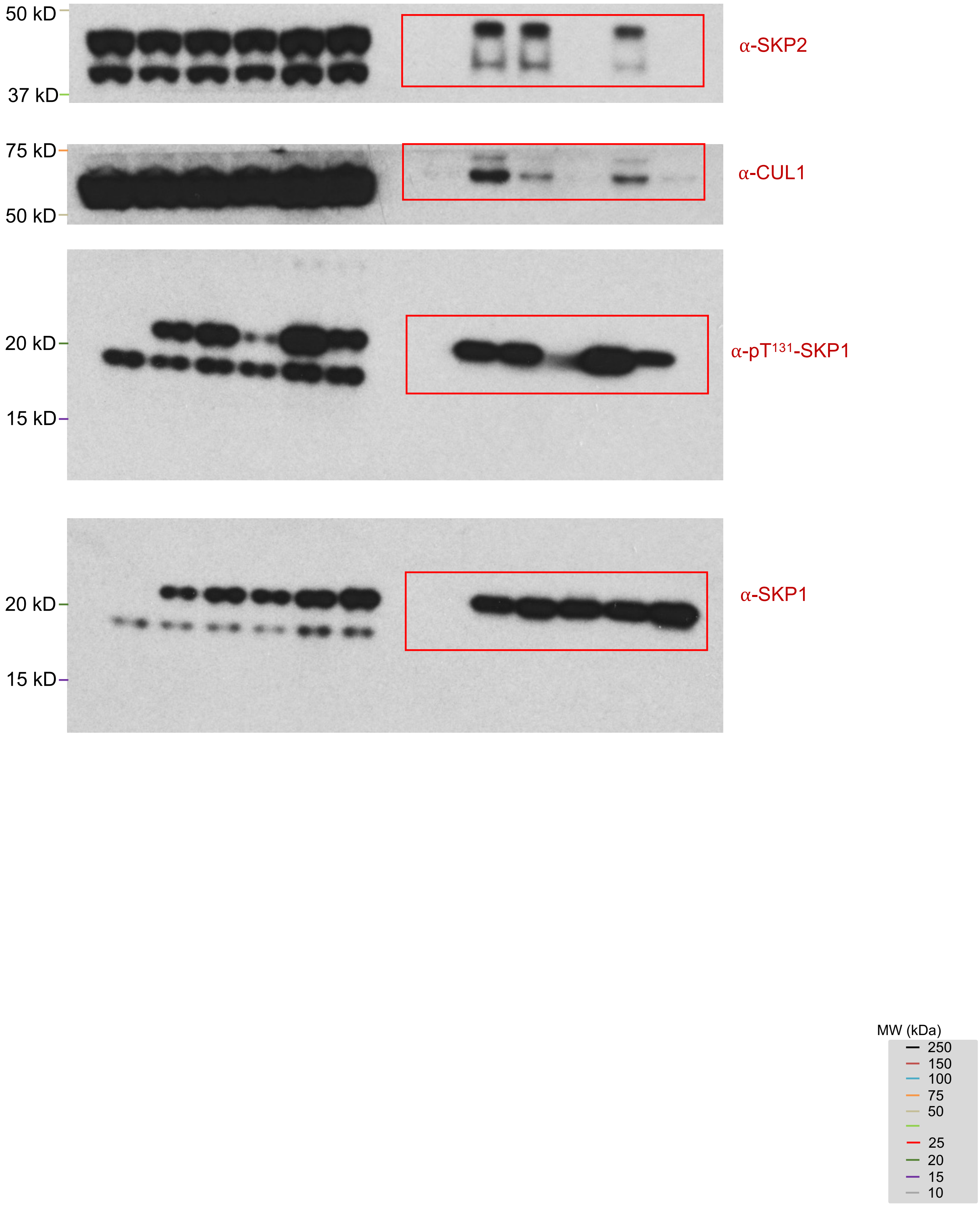

50 kD
37 kD
α-SKP2
75 kD
50 kD
α-CUL1
20 kD
15 kD
α-pT131-SKP1
20 kD
15 kD
α-SKP1
MW (kDa)
250
150
100
75
50
25
20
15
10

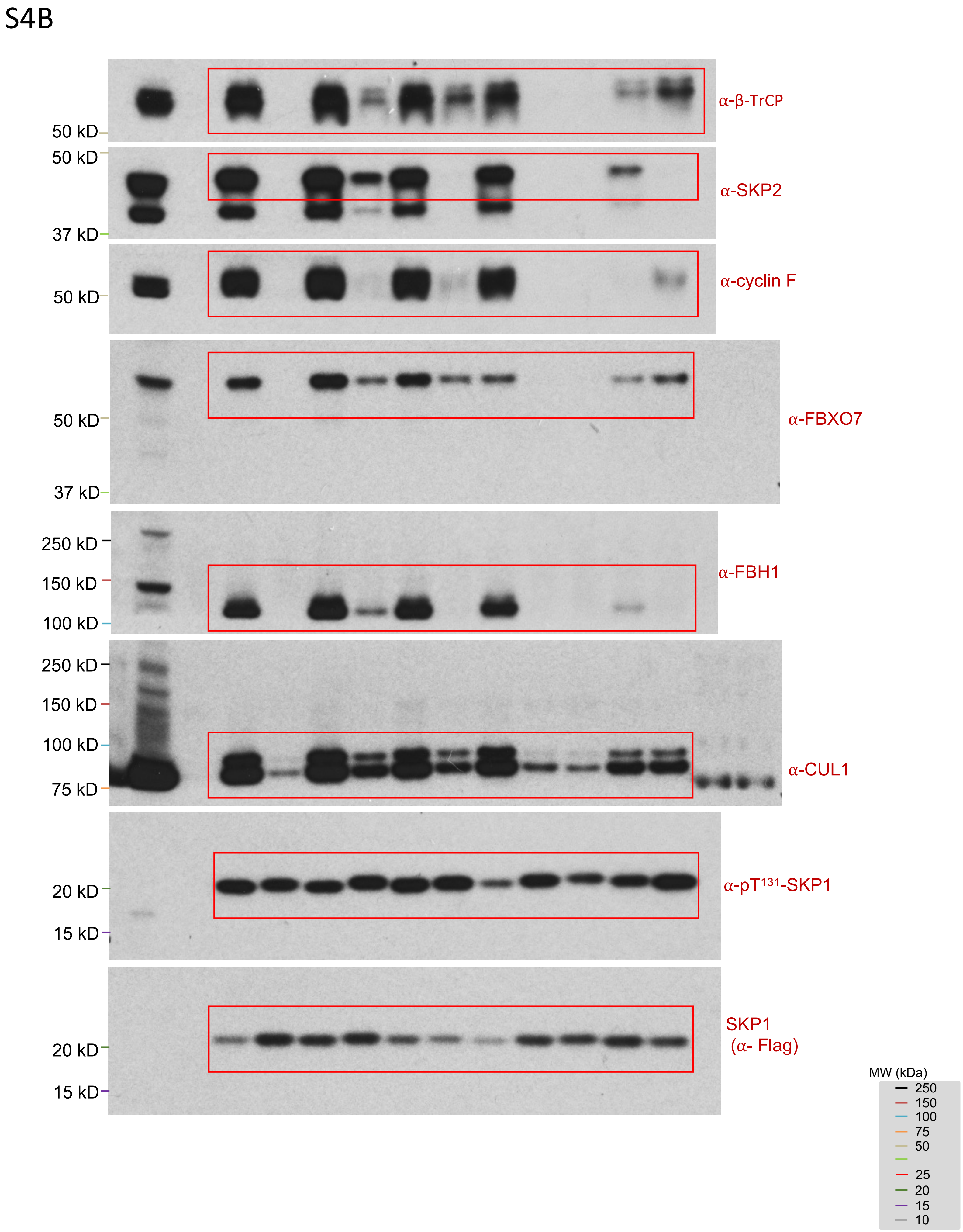

S4B
50 kD
α-β-TrCP
50 kD
37 kD
α-SKP2
50 kD
α-cyclin F
50 kD
37 kD
α-FBXO7
250 kD
150 kD
100 kD
α-FBH1
250 kD
150 kD
100 kD
75 kD
α-CUL1
20 kD
15 kD
α-pT131-SKP1
20 kD
15 kD
SKP1
(α- Flag)
MW (kDa)
250
150
100
75
50
25
20
15
10

S4C

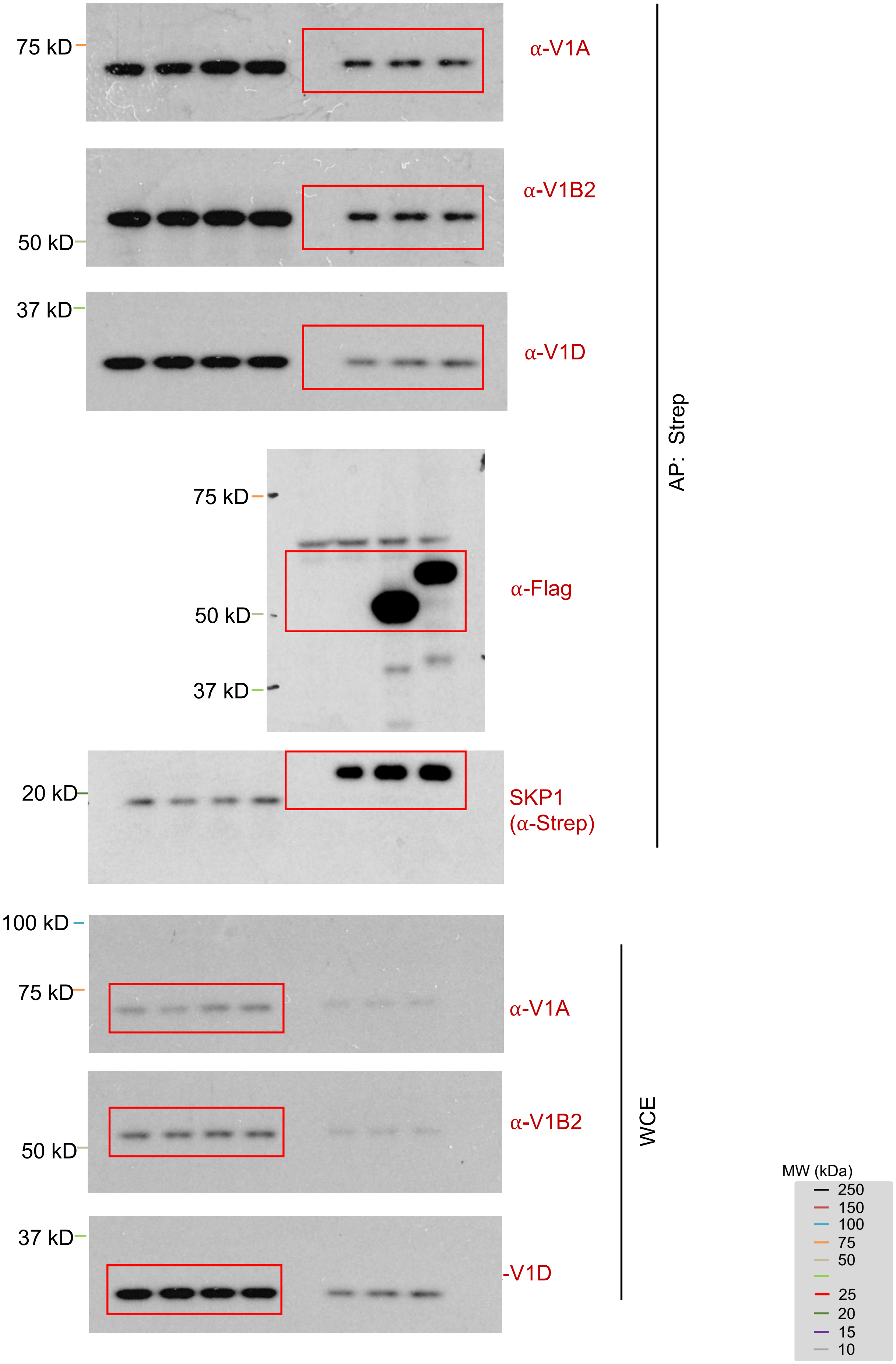
75 kD
α-V1A
α-V1B2
50 kD
37 kD
α-V1D
AP: Strep
75 kD
α-Flag
50 kD
37 kD
20 kD
SKP1
(α-Strep)
100 kD
75 kD
α-V1A
α-V1B2
50 kD
WCE
37 kD
-V1D
MW (kDa)
250
150
100
75
50
25
20
15
10

S4D

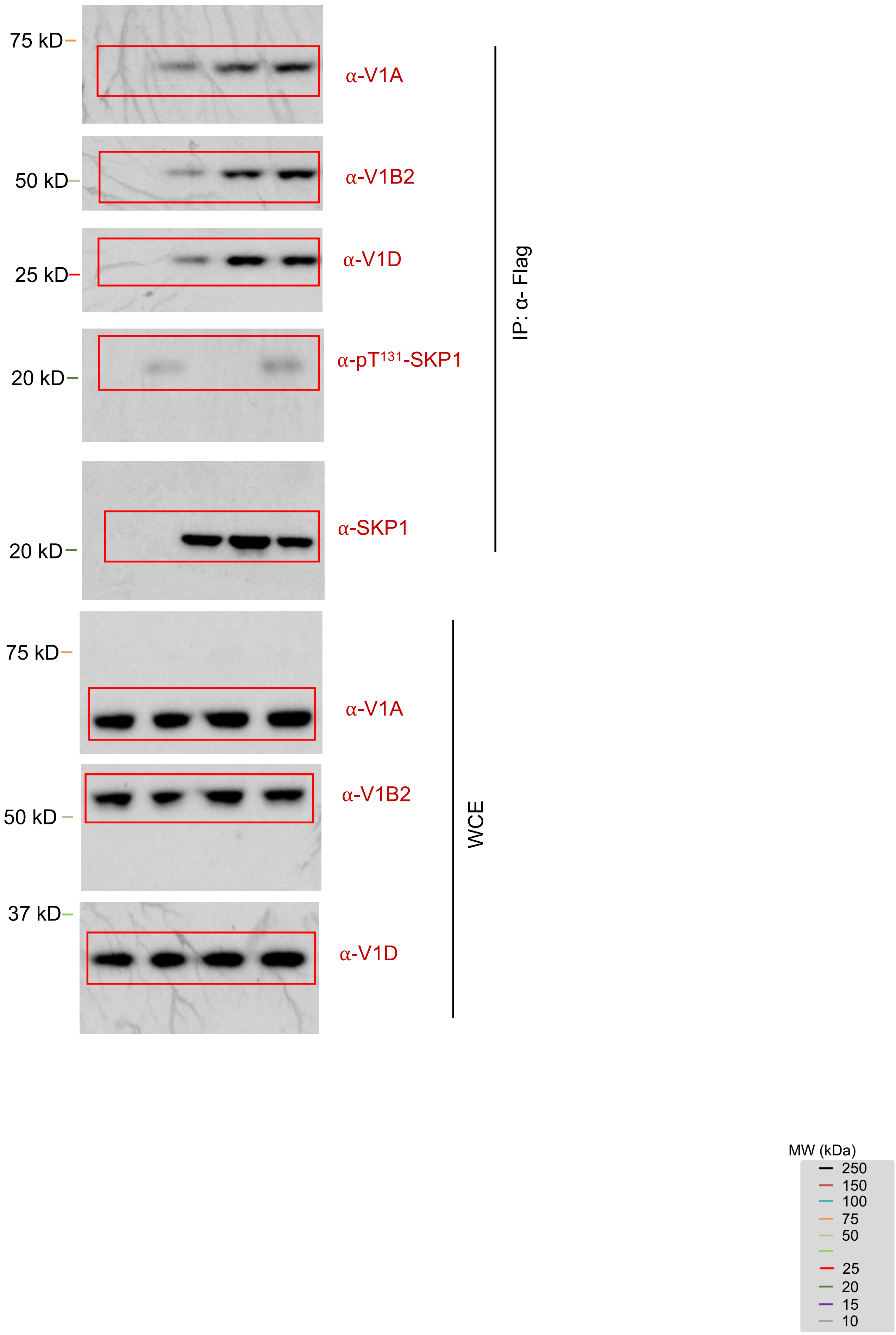
75 kD
α-V1A
50 kD
α-V1B2
25 kD
α-V1D
20 kD
α-pT131-SKP1
20 kD
α-SKP1
IP: α- Flag
75 kD
α-V1A
50 kD
α-V1B2
37 kD
α-V1D
WCE
MW (kDa)
250
150
100
75
50
25
20
15
10

S5A

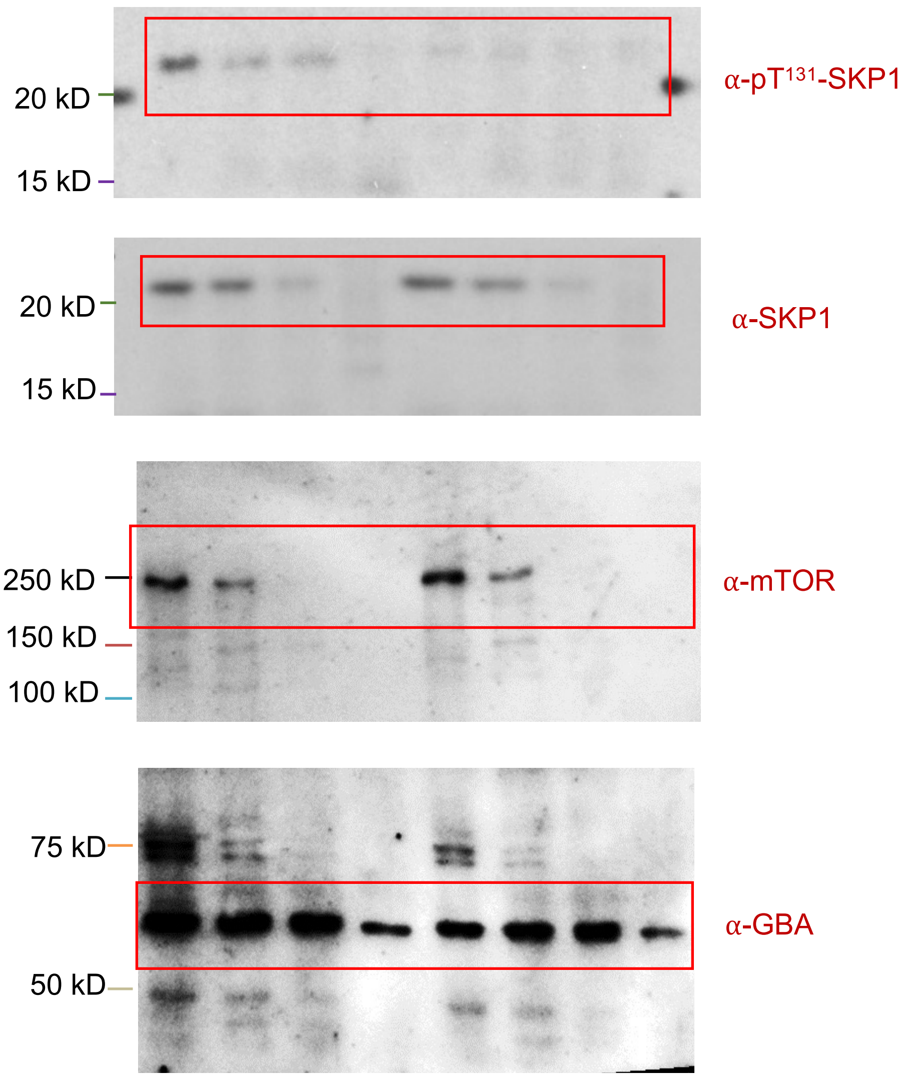

20 kD
15 kD
α-pT131-SKP1
20 kD
15 kD
α-SKP1
250 kD
150 kD
100 kD
α-mTOR
75 kD
50 kD
α-GBA

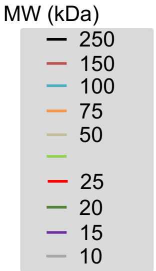

MW (kDa)
250
150
100
75
50
25
20
15
10

S5B

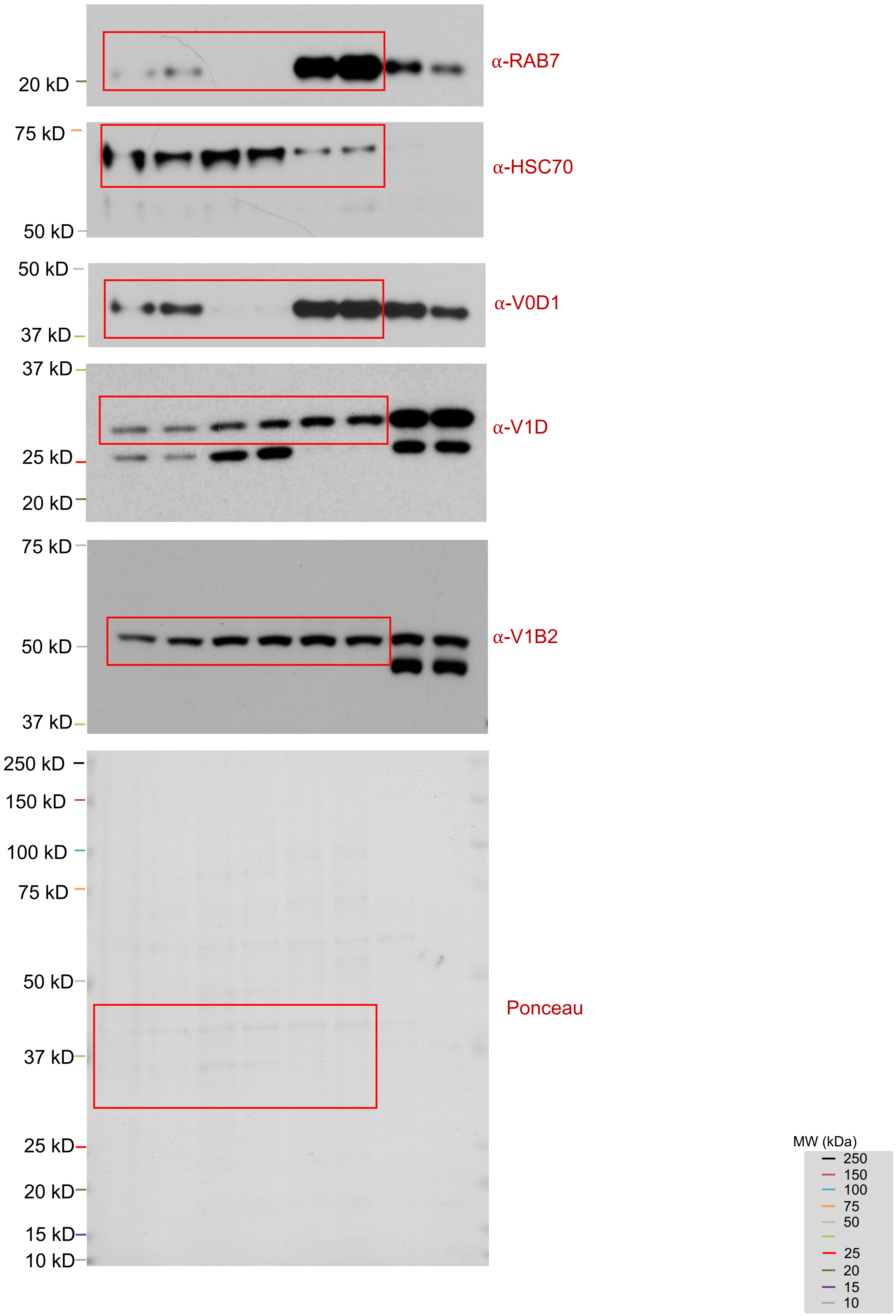
20 kD
α-RAB7
75 kD
α-HSC70
50 kD
50 kD
α-V0D1
37 kD
37 kD
α-V1D
25 kD
20 kD
75 kD
α-V1B2
50 kD
37 kD
250 kD
150 kD
100 kD
75 kD
50 kD
Ponceau
37 kD
25 kD
20 kD
15 kD
10 kD
MW (kDa)
250
150
100
75
50
25
20
15
10

S6A

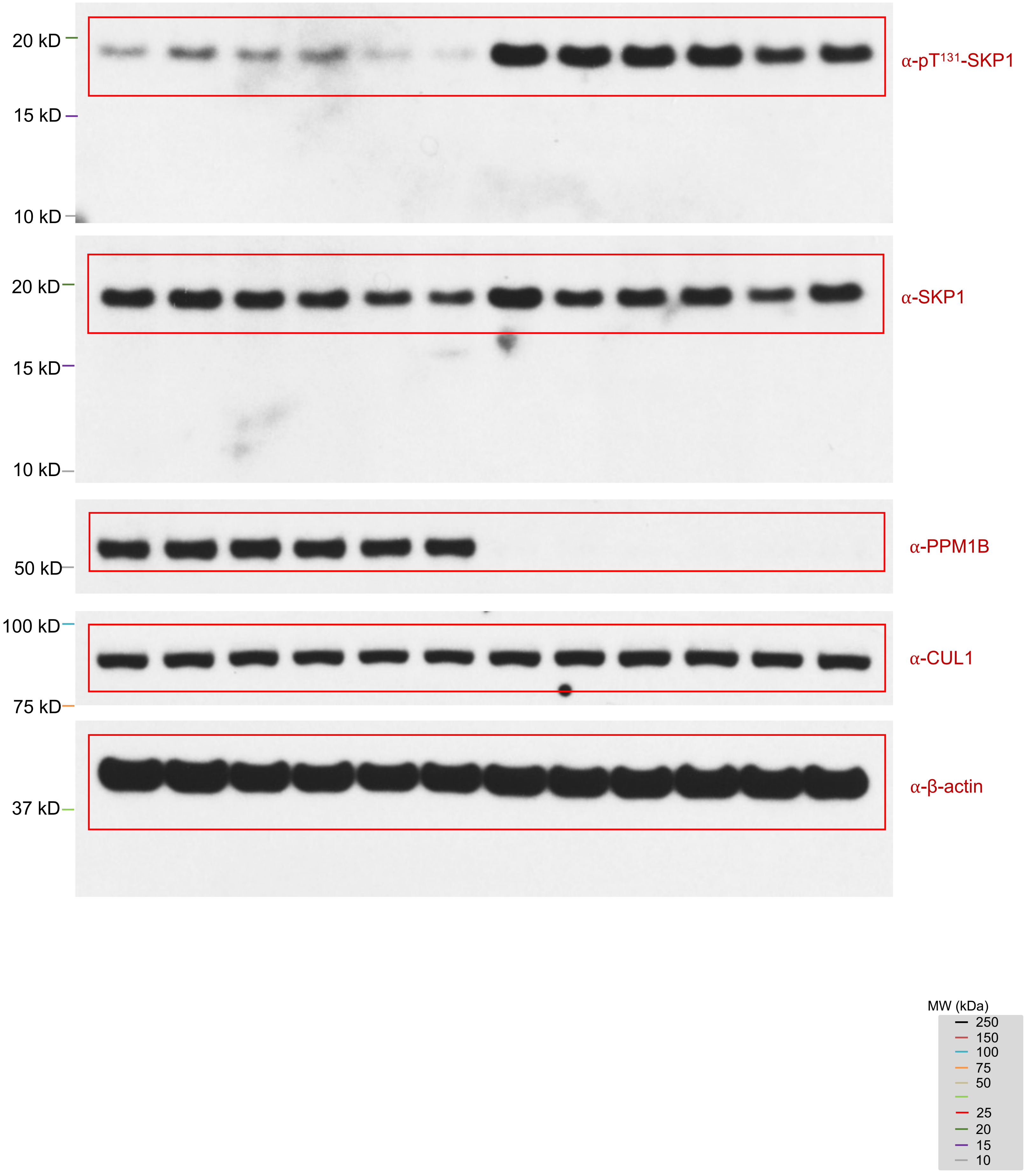
20 kD
15 kD
10 kD
α-pT131-SKP1
20 kD
15 kD
10 kD
α-SKP1
50 kD
α-PPM1B
100 kD
75 kD
α-CUL1
37 kD
α-β-actin
MW (kDa)
250
150
100
75
50
25
20
15
10

S6B

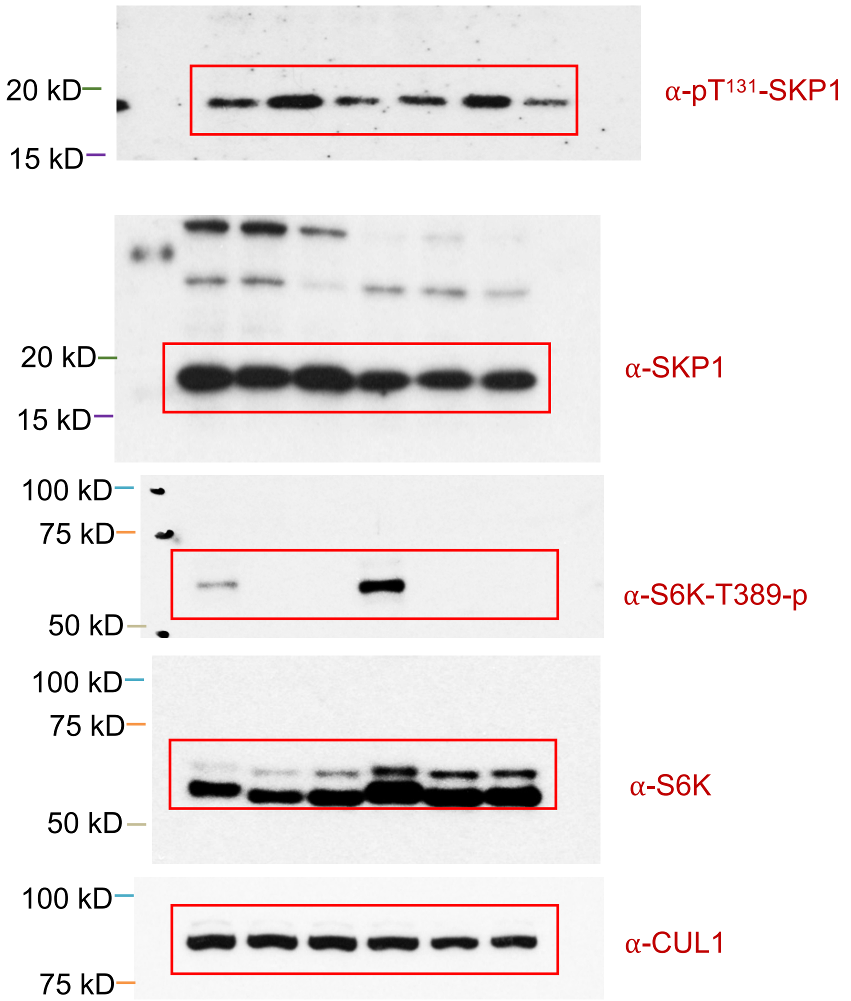

20 kD
15 kD
α-pT131-SKP1
20 kD
15 kD
α-SKP1
100 kD
75 kD
50 kD
α-S6K-T389-p
100 kD
75 kD
50 kD
α-S6K
100 kD
75 kD
α-CUL1

S6D

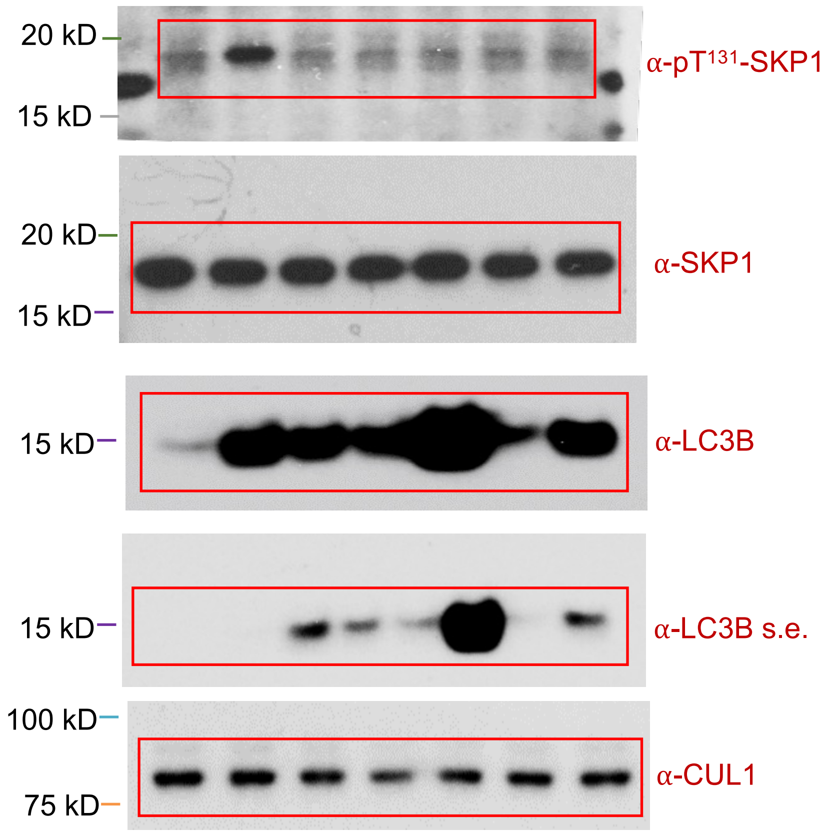

20 kD
15 kD
α-pT131-SKP1
20 kD
15 kD
α-SKP1
15 kD
α-LC3B
15 kD
α-LC3B s.e.
100 kD
75 kD
α-CUL1

S7A

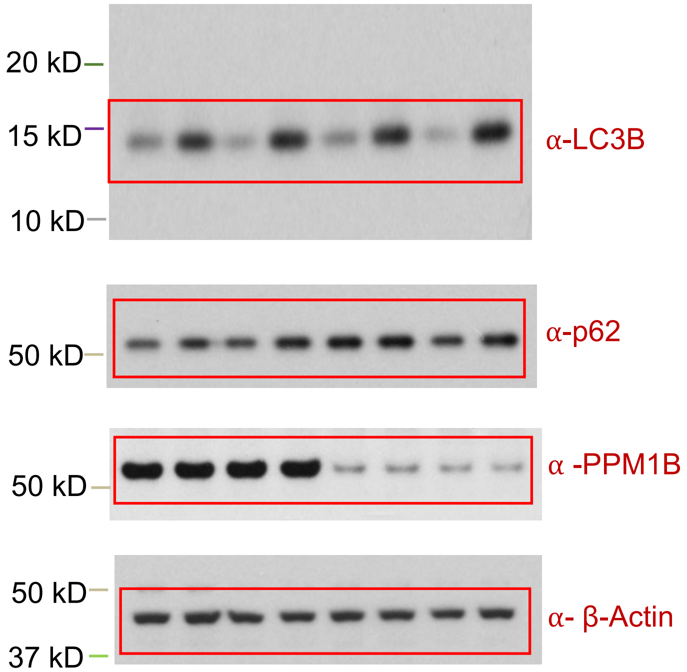

20 kD
15 kD
10 kD
α-LC3B
50 kD
α-p62
50 kD
α -PPM1B
50 kD
37 kD
α- β-Actin

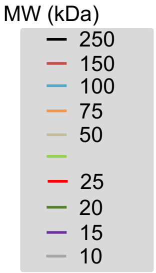

MW (kDa)
250
150
100
75
50
25
20
15
10

S10A

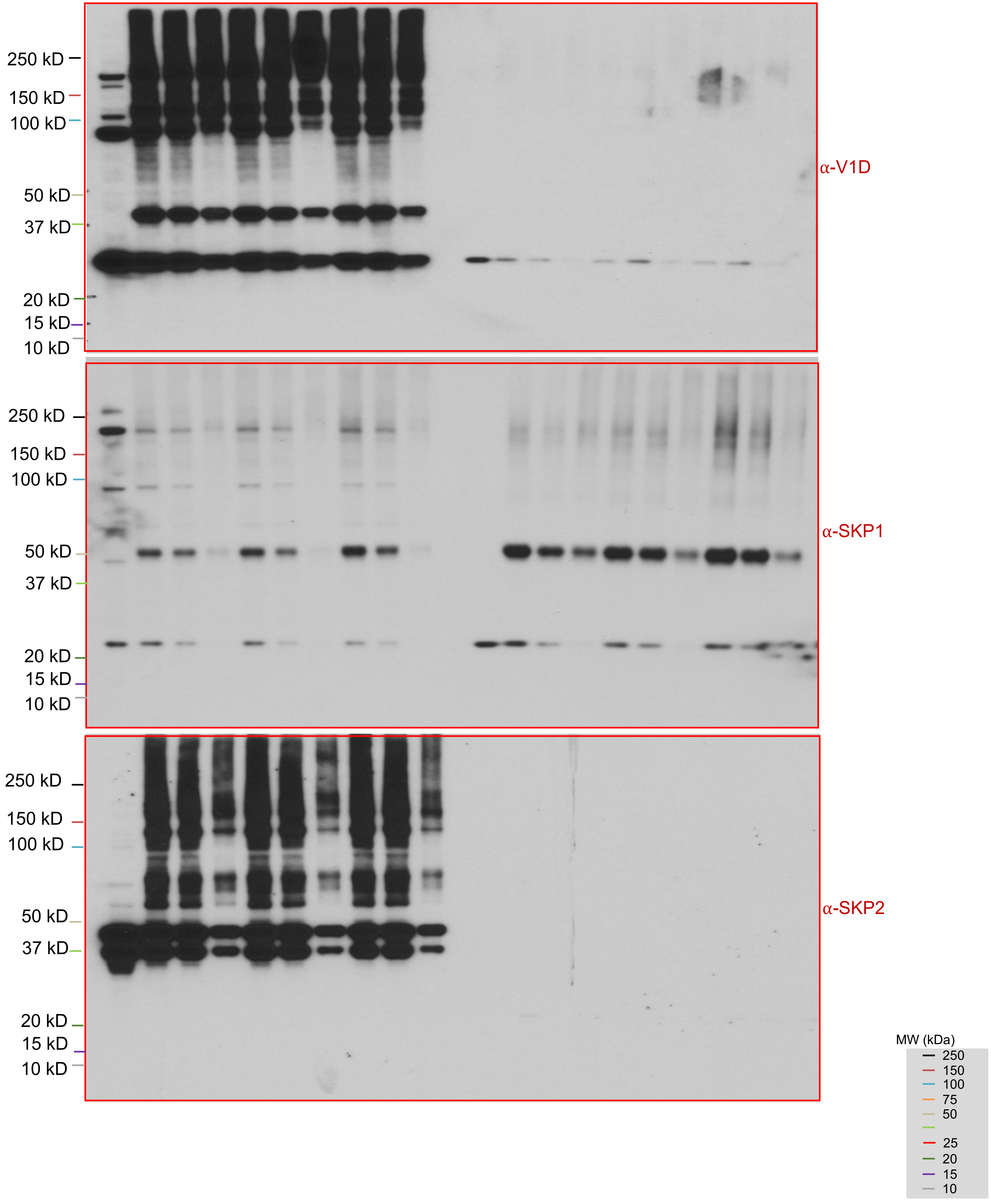
250 kD
150 kD
100 kD
50 kD
37 kD
20 kD
15 kD
10 kD
α-V1D
α-SKP1
α-SKP2
MW (kDa)
250
150
100
75
50
25
20
15
10

S11A

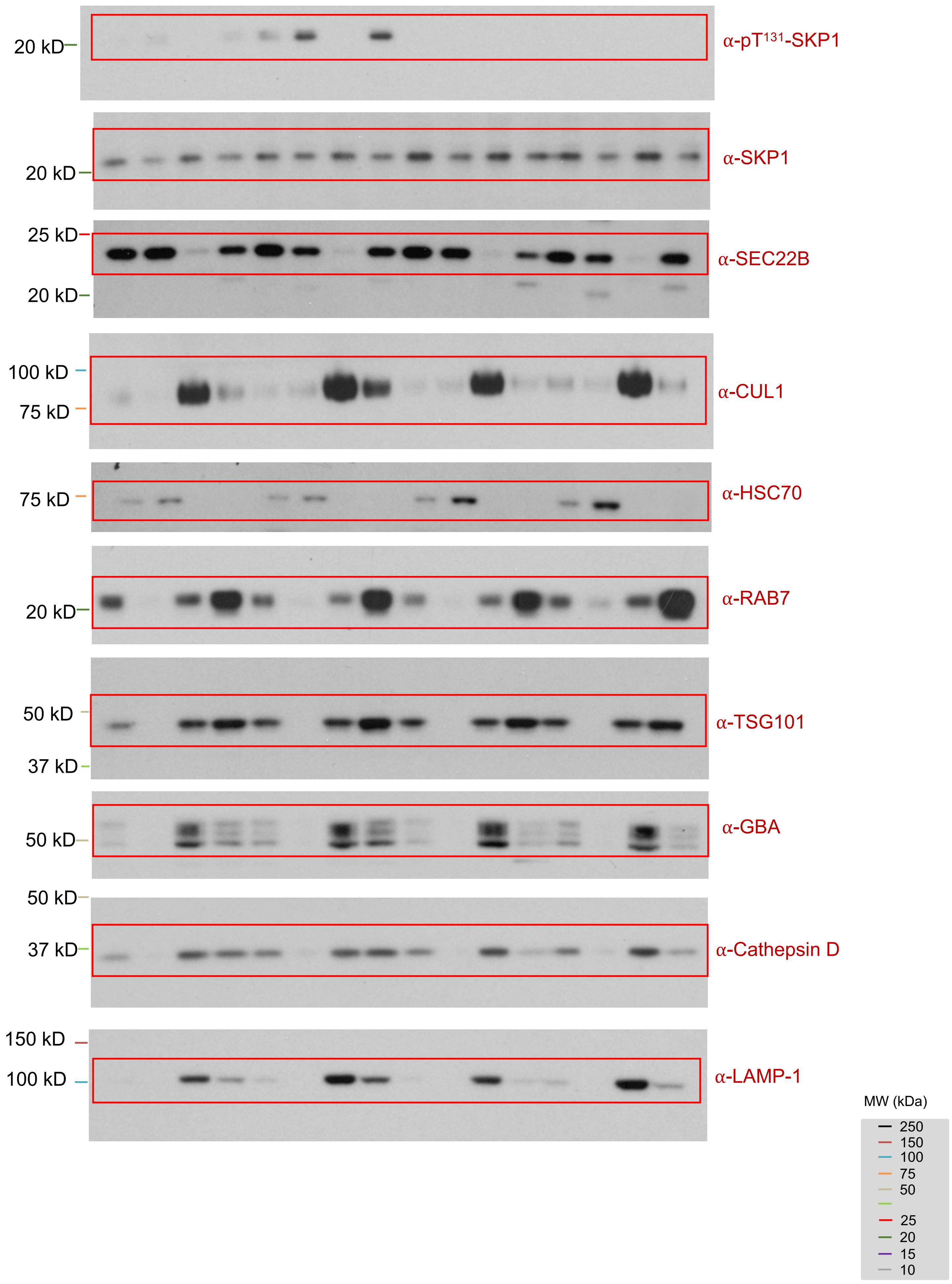

20 kD
α-pT131-SKP1
20 kD
α-SKP1
25 kD
20 kD
α-SEC22B
100 kD
75 kD
α-CUL1
75 kD
α-HSC70
20 kD
α-RAB7
50 kD
37 kD
α-TSG101
50 kD
α-GBA
50 kD
37 kD
α-Cathepsin D
150 kD
100 kD
α-LAMP-1
MW (kDa)
250
150
100
75
50
25
20
15
10

S11A

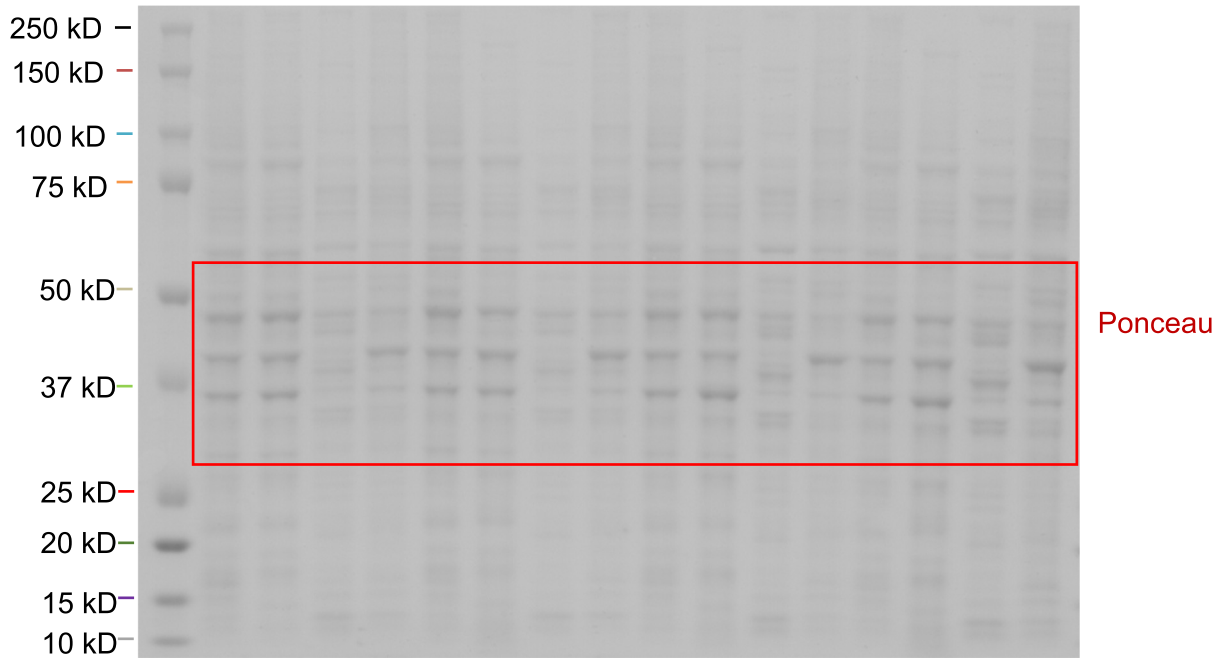

250 kD
150 kD
100 kD
75 kD
50 kD
37 kD
25 kD
20 kD
15 kD
10 kD
Ponceau

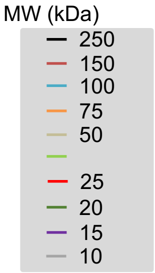

MW (kDa)
250
150
100
75
50
25
20
15
10

S11B

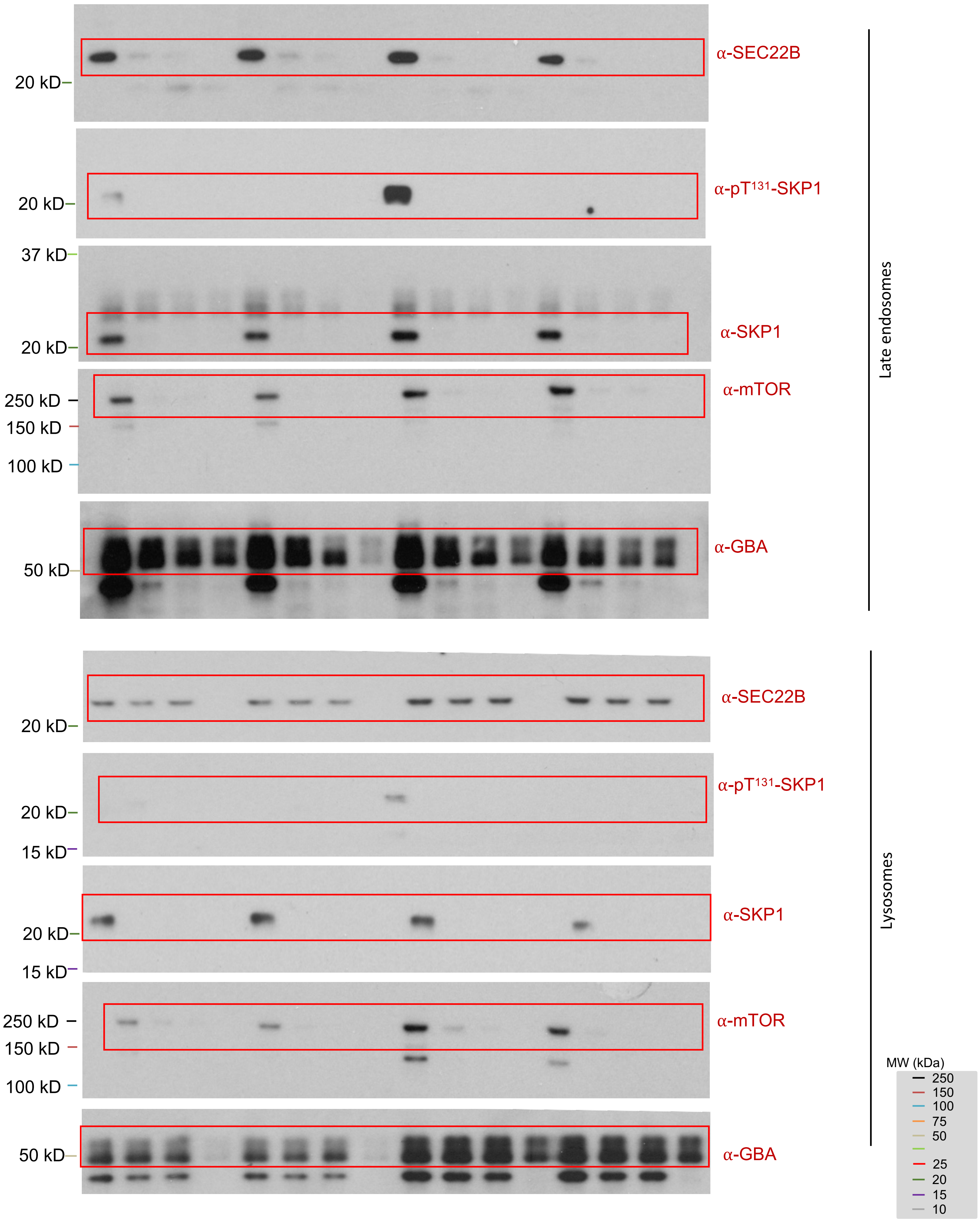
20 kD
α-SEC22B
20 kD
α-pT131-SKP1
37 kD
20 kD
α-SKP1
250 kD
150 kD
100 kD
α-mTOR
50 kD
α-GBA
Late endosomes
20 kD
α-SEC22B
20 kD
15 kD
α-pT131-SKP1
20 kD
15 kD
α-SKP1
250 kD
150 kD
100 kD
α-mTOR
50 kD
α-GBA
Lysosomes
MW (kDa)
250
150
100
75
50
25
20
15
10

S12A S12B

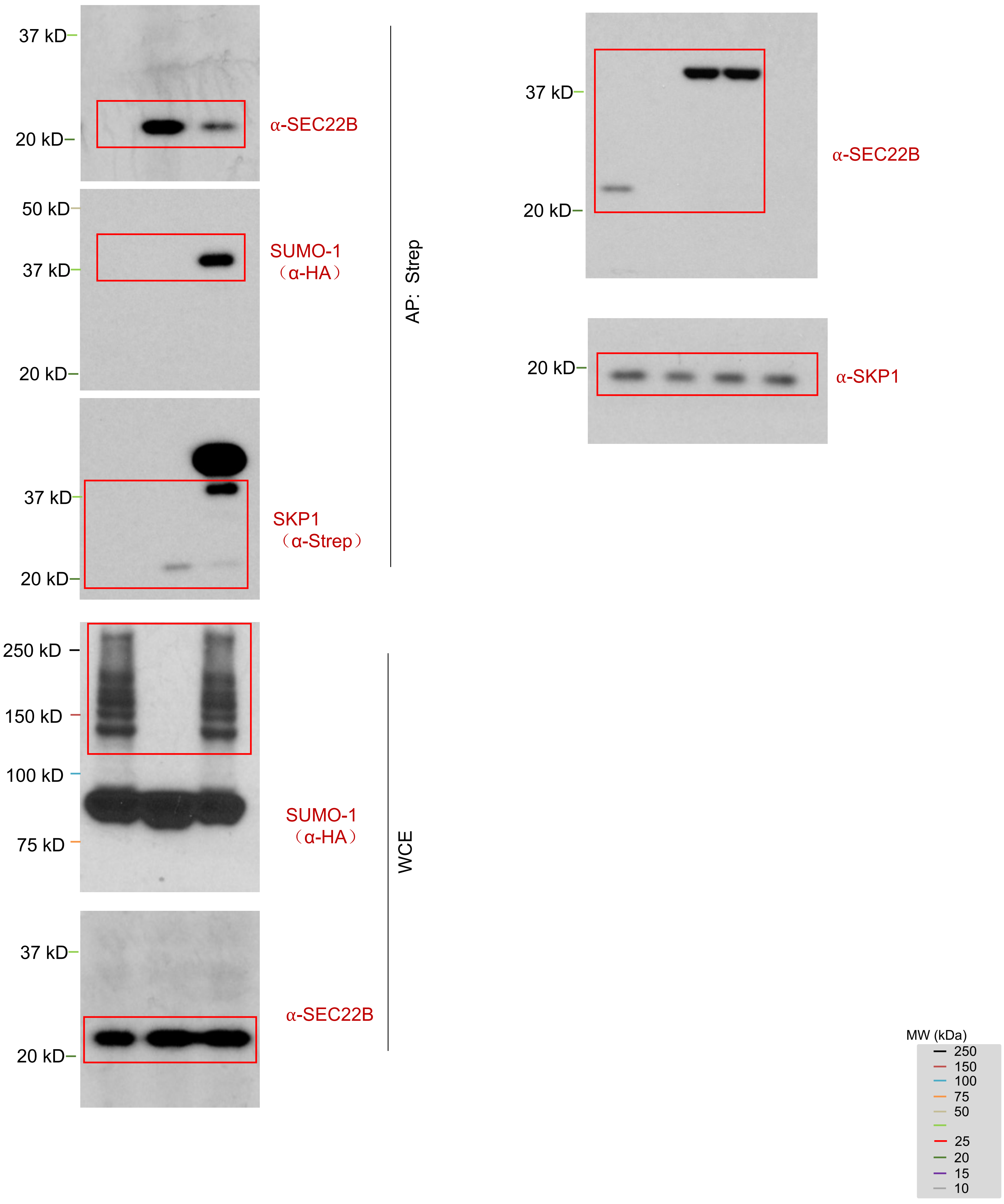
37 kD
20 kD
α-SEC22B
50 kD
37 kD
20 kD
SUMO-1
（α-HA）
37 kD
20 kD
SKP1
（α-Strep）
AP: Strep
250 kD
150 kD
100 kD
75 kD
SUMO-1
（α-HA）
37 kD
20 kD
α-SEC22B
WCE
37 kD
20 kD
α-SEC22B
20 kD
α-SKP1
MW (kDa)
250
150
100
75
50
25
20
15
10

S12C

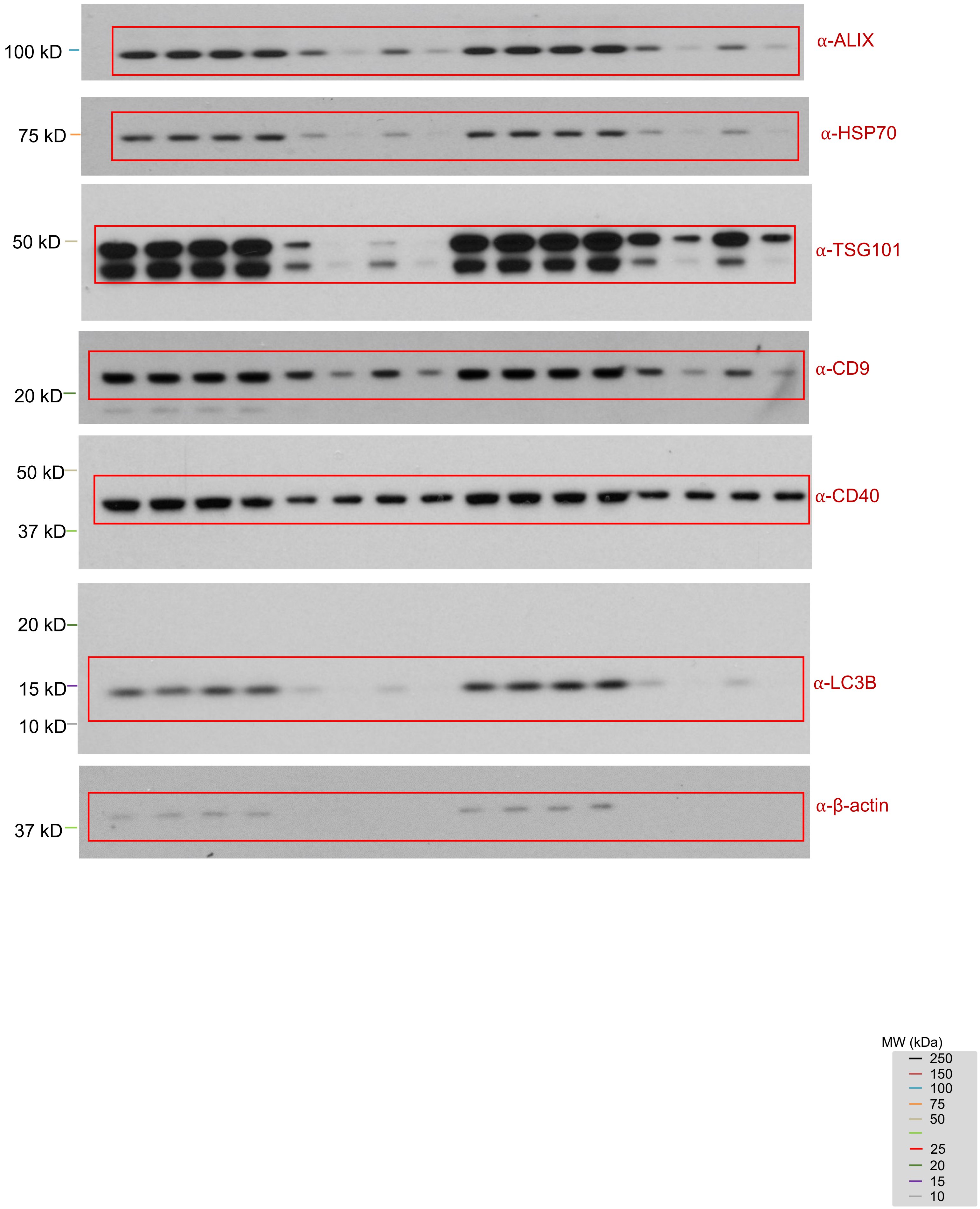
100 kD
α-ALIX
75 kD
α-HSP70
50 kD
α-TSG101
20 kD
α-CD9
50 kD
37 kD
α-CD40
20 kD
15 kD
10 kD
α-LC3B
37 kD
α-β-actin
MW (kDa)
250
150
100
75
50
25
20
15
10
